# Supplementary material for: Enantiospecificity in NMR enabled by chirality-induced spin selectivity
Source: Nat Commun. 2024 Aug 27;15:7367. doi: 10.1038/s41467-024-49966-8 (PMC11349874; doi:10.1038/s41467-024-49966-8)
Supplement: Supplementary file 1 — Supplementary Information [file 41467_2024_49966_MOESM1_ESM.pdf]

Enantiospecificity in NMR Enabled by Chirality-Induced Spin Selectivity

T. GEORGIOU, J.L. PALMA, V. MUJICA, S. VARELA, M. GALANTE, V. SANTAMARÍA GARCÍA,
L. MBONING, R.N. SCHWARTZ, G. CUNIBERTI, L.-S. BOUCHARD

TABLE OF CONTENTS

Supplementary Text
Text S1. Theory of Indirect Coupling in DNA Single Helix Model.
Text S2. Enantiospecificity in Cross-Polarization.
Text S3. J Coupling Stereochemical Deviations (DFT) for Amino Acids.
Text S4. Raw Data for J Couplings (DFT) in Amino Acids.

1. Text S1. Theory of Indirect Coupling in DNA Single Helix Model

Our theoretical model to derive the effective Hamiltonian is based on second-order perturbation theory. Effective Hamiltonians can be obtained by partitioning the Hamiltonian matrix into low energy (0) and high-energy blocks (1):

$$H = \begin{bmatrix} H_{00} & T_{01} \\ T_{10} & H_{11} \end{bmatrix}.$$

To this matrix corresponds a resolvent (Green's function):

$$G(\epsilon) = \frac{1}{\epsilon - H} = \begin{bmatrix} \epsilon - H_{00} & T_{01} \\ T_{10} & \epsilon - H_{11} \end{bmatrix}^{-1}.$$

Inversion of the block 2×2 matrix gives:

$$G_{00}(\epsilon) = \left(\epsilon - H_{00} - T_{01}(\epsilon - H_{11})^{-1}T_{10}\right)^{-1},$$

which has the form of a resolvent with effective Hamiltonian:

$$G(\epsilon) = \frac{1}{\epsilon - H_{eff}}, \qquad H_{eff} = H_{00} + T_{01}(\epsilon - H_{11})^{-1}T_{10}.$$

Because H_{eff} itself depends on the unknown energy ϵ, the Schrödinger equation is then solved self-consistently. This method was introduced by Löwdin [1] and is nowadays known as downfolding. If instead we replace the (unknown) energy ϵ by a known energy of the low-energy states, ϵ_0:

$$\approx H_{00} + T_{01}(\epsilon_0 - H_{11})^{-1}T_{10},$$

taking the matrix element yields the familiar expression for second-order perturbation theory:

$$E_0 = \epsilon_0 + \sum_{j,j'} \langle 0|T_{01}|j\rangle \langle j|(\epsilon_0 - H_{11})^{-1}|j'\rangle \langle j'|T_{10}|0\rangle = \epsilon_0 + \sum_j \frac{|\langle 0|\hat{V}|j\rangle|^2}{\epsilon_0 - \epsilon_j}$$

where $\hat{V} \equiv T_{01}$, $T_{10}^{\dagger} = T_{01}$ and H_{11} is assumed diagonalized by the unperturbed basis $\{|j\rangle\}$. A pedagogical description of the perturbation theory approach to deriving effective spin-Hamiltonians can be found in chapter 4 of Slichter [2]. Electronic wavefunctions, which encode chirality, were obtained from the following papers by Varela and co-workers [3, 4]. In the work of Santos and San Sebastian [5, 6] a mechanism for indirect nuclear spin-spin coupling was proposed based on spin-orbit coupling (SOC). SOC is indeed expected to play a crucial role. The exact mechanism for the indirect coupling, however, remains unclear. In our calculation and proposed mechanism, SOC enters through the use of the Varela wavefunctions [3, 4] as part of the perturbation theoretic calculation. We find that the magnetic interaction that can explain the existence of the effective spin-Hamiltonian is dipolar in nature. Nuclear spin 1 couples magnetically to delocalized conduction band elections, which then couple magnetically to nuclear spin 2.

1.1. **CISS & Indirect Spin-Spin Coupling.**

The magnetic dipole interaction between two spins $\mathbf{I}_1$ and $\mathbf{I}_2$ is of the form of a scalar product of irreducible rank-2 tensors, $[\mathbf{I}_1 \otimes \mathbf{I}_2]^{(2)}$ and $[\nabla \otimes \nabla]^{(2)} \frac{1}{r}$:

$$\mathcal{H} = -3\sqrt{5}\frac{\mu_0}{4\pi}\hbar^2\gamma_I\gamma_S \left[[\mathbf{I}_1 \otimes \mathbf{I}_2]^{(2)} \otimes [\nabla \otimes \nabla]^{(2)}(1/r)\right]_0^{(0)}$$

where $[\mathbf{A} \otimes \mathbf{B}]_0^{(0)}$ denotes the scalar tensor product of two irreducible tensors $\mathbf{A}$ and $\mathbf{B}$, with the convention that all vectors are expressed as rank-1 spherical tensors, i.e. for $\mathbf{r} = (x, y, z)$ we have $\mathbf{r}_{(1),\pm 1} = \mp(1/\sqrt{2})(x \pm iy)$, $r_0^{(1)} = z$. The irreducible components of the product of two tensors $\mathbf{T}_{(k_1)}$ and $\mathbf{T}_{(k_2)}$ are obtained as the linear combinations:

$$\left[\mathbf{T}_{(k_1)} \otimes \mathbf{T}_{(k_2)}\right]_{\kappa_3}^{(k_3)} = \sum_{\kappa_1,\kappa_2} (k_1\kappa_1 k_2\kappa_2 | k_1 k_2 k_3 \kappa_3)\, \mathbf{T}_{(k_1),\kappa_1} \mathbf{T}_{(k_2),\kappa_2},$$

where $(k_1\kappa_1 k_2\kappa_2 | k_1 k_2 k_3 \kappa_3)$ are Clebsch-Gordan coefficients. This formula can be used to describe any number of pairwise magnetic interactions, whose contributions to the energy are additive, provided that extra care is taken at the point $r = 0$, by adding a Dirac delta function. The latter is a result from distribution theory (see below). Two localized spins $\mathbf{I}_1$ and $\mathbf{I}_2$ centered at $\mathbf{R}_1$ and $\mathbf{R}_2$, respectively, interact with a set of electron spins $\{\mathbf{S}_l\}$ each centered at $\{\mathbf{r}_l\}$ according to the sum of Fermi contact and dipole-dipole interactions (energy units):

$$\mathcal{H}(\{\mathbf{R}_i\}, \{\mathbf{r}_l\}) = \frac{\mu_0}{4\pi}\hbar^2 \Bigg[\underbrace{\frac{8\pi}{3}\gamma_I\gamma_S \mathbf{I}_1 \cdot \sum_l \mathbf{S}_l \delta^{(3)}(\mathbf{R}_1 - \mathbf{r}_l) + \frac{8\pi}{3}\gamma_I\gamma_S \mathbf{I}_2 \cdot \sum_l \mathbf{S}_l \delta^{(3)}(\mathbf{R}_2 - \mathbf{r}_l)}_{\text{Fermi contact}, \mathcal{H}_{Fc}(\{\mathbf{R}_i\},\{\mathbf{r}_l\})}$$

$$+ \underbrace{\gamma_I\gamma_S\, p.v. \sum_l \frac{\delta_{\alpha\beta} - 3\hat{\tilde{r}}_{1l,\alpha}\hat{\tilde{r}}_{1l,\beta}}{|\mathbf{R}_1 - \mathbf{r}_l|^3} I_1^\alpha S_l^\beta + \gamma_I\gamma_S\, p.v. \sum_l \frac{\delta_{\alpha\beta} - 3\hat{\tilde{r}}_{2l,\alpha}\hat{\tilde{r}}_{2l,\beta}}{|\mathbf{R}_2 - \mathbf{r}_l|^3} I_2^\alpha S_l^\beta}_{\text{dipole-dipole}, \mathcal{H}_{DD}(\{\mathbf{R}_i\},\{\mathbf{r}_l\})} \Bigg]$$

where $p.v.$ denotes Cauchy principal value, and the component of unit vector $\hat{\tilde{r}}_{il,\alpha}$ are:

$$\hat{\tilde{r}}_{il,\alpha} = \frac{R_{i,\alpha} - r_{l,\alpha}}{\mathbf{R}_i - \mathbf{r}_l}.$$

Since $\mathbf{I}_1$ and $\mathbf{I}_2$ are assumed to be localized spins, $\mathbf{R}_1$ and $\mathbf{R}_2$ are treated as fixed parameters.

An effective Hamiltonian can be obtained through second-order perturbation theory:

$$\mathcal{H}_{eff} = \underbrace{\left(\frac{2\mu_0}{3}\right)^2 \gamma_I^2\gamma_S^2\hbar^4 \sum_j \mathbf{I}_1 \cdot \frac{\langle 0| \sum_l \mathbf{S}_l \delta^{(3)}(\mathbf{r}_l - \mathbf{R}_1)|j\rangle \langle j| \sum_l \mathbf{S}_l \delta^{(3)}(\mathbf{r}_l - \mathbf{R}_2)|0\rangle}{E_0 - E_j} \cdot \mathbf{I}_2 + c.c.}_{\mathcal{H}_{eff}^{FC}}.$$

(1)

$$+ \underbrace{\left(\frac{\mu_0}{4\pi}\right)^2 \gamma_I^2\gamma_S^2\hbar^4\, p.v. \sum_{\alpha,\alpha'}\sum_{\beta,\beta'}\sum_j I_1^\alpha \frac{\langle 0| \sum_l \frac{\delta_{\alpha\beta} - 3\hat{\tilde{r}}_{1l,\alpha}\hat{\tilde{r}}_{1l,\beta}}{|\mathbf{R}_1 - \mathbf{r}_l|^3} S_l^\beta |j\rangle \langle j| \sum_l \frac{\delta_{\alpha'\beta'} - 3\hat{\tilde{r}}_{2l,\alpha'}\hat{\tilde{r}}_{2l,\beta'}}{|\mathbf{R}_2 - \mathbf{r}_l|^3} S_l^{\beta'} |0\rangle}{E_0 - E_j} I_2^{\alpha'} + c.c.}_{\mathcal{H}_{eff}^{DD}}$$

It will be convenient for us to express the dipole interaction tensor in terms of the Hessian of $1/\tilde{r}$:

$$\nabla\nabla \frac{1}{|\mathbf{r} - \mathbf{r}'|} = p.v. \left(-\frac{\delta_{ij}}{|\mathbf{r} - \mathbf{r}'|^3} + \frac{3(r_i - r_i')(r_j - r_j')}{|\mathbf{r} - \mathbf{r}'|^5} \right) \hat{\mathbf{e}}_i \hat{\mathbf{e}}_j - \frac{4\pi}{3}\delta^{(3)}(\mathbf{r} - \mathbf{r}') I$$

where $p.v.$ denotes the Cauchy principal value and I is the identity tensor. The Dirac delta term is an artifact from distribution theory originating from the point $\mathbf{r} = \mathbf{r}'$. The Dirac

term is of no consequence here, as we show (see Section 1.3) that Fermi contact terms do not contribute significantly to the effective interaction in the context of CISS.

1.1.1. *Matrix Elements of Single-Body Operators.* In the above expression, we have matrix elements of $\sum_l \mathbf{S}_l \delta^{(3)}(\mathbf{r}_l - \mathbf{R})$, which is a sum of single-body operators. The states $|0\rangle$ and $|j\rangle$ are Slater determinants that are products of Bloch functions. In the language of second-quantization a single-body operator $\mathbf{H}$ has the form

$$\mathbf{H} = \underbrace{\sum_{i=1}^{N} \mathbf{h}(i)}_{\text{first quantization}} = \underbrace{\sum_{m,n} \langle m|\mathbf{H}|n\rangle\, a_m^\dagger a_n,}_{\text{second quantization}}$$

where $a_m^\dagger$ and a_n are fermionic creation and annihilation operators, respectively. The equal signs here are taken loosely in the sense that the different $\mathbf{H}$'s involved operate on different spaces (e.g. Hilbert vs Fock space). The N-body wavefunctions are Slater determinants:

$$|0\rangle = a_{j_1}^\dagger a_{j_2}^\dagger \dots a_{j_N}^\dagger |vac\rangle$$
$$|j\rangle = a_{j_{1'}}^\dagger a_{j_{2'}}^\dagger \dots a_{j_{N'}}^\dagger |vac\rangle\,,$$

where $|vac\rangle$ denotes the vacuum state. The matrix elements of $\mathbf{H}$ are:

$$\begin{aligned}\langle j|\mathbf{H}|0\rangle &= \langle vac|\, a_{j_{N'}} \dots a_{j_{2'}} a_{j_{1'}} \cdot \sum_{m,n} \langle m|\mathbf{H}|n\rangle\, a_m^\dagger a_n \cdot a_{j_1}^\dagger a_{j_2}^\dagger \dots a_{j_N}^\dagger |vac\rangle \\ &= \sum_{m,n} \langle m|\mathbf{H}|n\rangle \langle vac|\, a_{j_{N'}} \dots a_{j_{2'}} a_{j_{1'}} a_m^\dagger a_n a_{j_1}^\dagger a_{j_2}^\dagger \dots a_{j_N}^\dagger |vac\rangle\end{aligned}$$

Using anticommutation relations for the fermionic operators:

$$\{a_j^\dagger, a_{j'}\} = \delta_{jj'}, \qquad \{a_j, a_{j'}\} = 0, \qquad \{a_j^\dagger, a_{j'}^\dagger\} = 0,$$

we see that moving the a_n operator to the right will annihilate the vacuum state to give zero unless $n \in \{j_1, j_2, \dots, n_N\}$. If n belongs to that set, say $n = j_k$, then:

$$\begin{aligned}a_n a_{j_1}^\dagger \dots a_{j_k}^\dagger \dots a_{j_N}^\dagger |vac\rangle =& (-1)^{k-1} a_{j_1}^\dagger \dots \underbrace{(a_n a_{j_k}^\dagger)}_{1 - a_{j_k}^\dagger a_n} \dots a_{j_N}^\dagger |vac\rangle \\ =& (-1)^{k-1} a_{j_1}^\dagger \dots (1 - a_{j_k}^\dagger a_n) \dots a_{j_N}^\dagger |vac\rangle \\ =& (-1)^{k-1} a_{j_1}^\dagger \dots (1) \dots a_{j_N}^\dagger |vac\rangle \\ &- (-1)^{k-1} a_{j_1}^\dagger \dots a_{j_k}^\dagger \dots a_{j_N}^\dagger a_n (-1)^{N-k} |vac\rangle\end{aligned}$$

The second term is zero because a_n acts on the vacuum. Likewise, moving $a_m^\dagger$ to the left, we get (say $m = j_{d'}$):

$$\begin{aligned}\langle vac|\, a_{j_{N'}} \dots a_{j_{d'}} \dots a_{j_{2'}} a_{j_{1'}} a_m^\dagger &= \langle vac|\, a_{j'_N} \dots \underbrace{(a_{j_{d'}} a_m^\dagger)}_{1 - a_m^\dagger a_{j_{d'}}} \dots a_{j'_2} a_{j'_1} (-1)^{d'-1} \\ &= \langle vac|\, a_{j_{N'}} \dots (1 - a_m^\dagger a_{j_{d'}}) \dots a_{j_{2'}} a_{j_{1'}} (-1)^{d'-1} \\ &= \langle vac|\, a_{j_{N'}} \dots (1) \dots a_{j_{2'}} a_{j_{1'}} (-1)^{d'-1} \\ &\quad - \langle vac|\, a_m^\dagger a_{j_{N'}} \dots a_{j_{d'}} \dots a_{j'_2} a_{j_{1'}} (-1)^{d'-1} (-1)^{N-d'}\end{aligned}$$

The second term is zero as $a_m^\dagger$ acts on $\langle vac|$ to its left. Thus, we are left with:

$$\langle vac|\, a_{j_{N'}} \dots a_{j_{d'}} \dots a_{j_{2'}} a_{j_{1'}} (a_m^\dagger a_n) a_{j_1}^\dagger \dots a_{j_k}^\dagger \dots a_{j_N}^\dagger \,|vac\rangle$$
$$= \langle vac|\, a_{j_{N'}} \dots (1) \dots a_{j_{2'}} a_{j_{1'}} a_{j_1}^\dagger \dots (1) \dots a_{j_N}^\dagger \,|vac\rangle\, (-1)^{d'+k}$$

which is zero unless $\{j_1, \dots, \hat{j}_k, \dots, j_N\} = \{j_{1'}, \dots, \hat{j}_{d'}, \dots, j_{N'}\}$ (where the hat indicates omission of a term). Thus, the average $\langle j|a_m^\dagger a_n|0\rangle$ is zero unless $n \in \{j_1, \dots, j_N\}$, $d' \in \{j_{1'}, \dots, j_{N'}\}$ and the remaining indices are all identical $\{j_1, \dots, \hat{j}_k, \dots, j_N\} = \{j_{1'}, \dots, \hat{j}_{d'}, \dots, j_{N'}\}$. If the remaining indices are identical (but not necessarily in the same order), then

$$\langle vac|\, a_{j_{N'}} \dots (1) \dots a_{j_{2'}} a_{j_{1'}} a_{j_1}^\dagger \dots (1) \dots a_{j_N}^\dagger \,|vac\rangle$$
$$= (-1)^P \langle vac|\, a_{j_N} \dots (1) \dots a_{j_2} a_{j_1} a_{j_1}^\dagger \dots (1) \dots a_{j_N}^\dagger \,|vac\rangle = (-1)^P,$$

through repeated application of the anticommutator $a_m a_m^\dagger = 1 - a_m^\dagger a_m$ in the center term (and dropping $a_m^\dagger a_m$ because it vanishes when moving a_m all the way to the right). P is the permutation needed to bring them to the same order.

We have just proved that:

$$\begin{aligned}
\langle j|\mathbf{H}|0\rangle &= \langle vac|\, a_{j_{N'}} \dots a_{j_{2'}} a_{j_{1'}} \mathbf{H} a_{j_1}^\dagger a_{j_2}^\dagger \dots a_{j_N}^\dagger \,|vac\rangle \\
&= \langle vac|\, a_{j_{N'}} \dots a_{j_{2'}} a_{j_{1'}} \left(\sum_{m,n} \langle m|\mathbf{H}|n\rangle\, a_m^\dagger a_n \right) a_{j_1}^\dagger a_{j_2}^\dagger \dots a_{j_N}^\dagger \,|vac\rangle \\
&= \sum_{\substack{m\in\langle j| \\ n\in|0\rangle}} \langle m|\mathbf{H}|n\rangle\, \langle vac|\, a_{j_{N'}} \dots a_{j_{2'}} a_{j_{1'}} (a_m^\dagger a_n) a_{j_1}^\dagger a_{j_2}^\dagger \dots a_{j_N}^\dagger \,|vac\rangle \\
&= \begin{cases} \sum_{\substack{m\in\langle j| \\ n\in|0\rangle}} \langle vac|\, a_m^\dagger \mathbf{H} a_n \,|vac\rangle\, (-1)^{d'+k+P} & \text{if } \{j_1, \dots, \hat{j}_k, \dots, j_N\} = \{j_{1'}, \dots, \hat{j}_{d'}, \dots, j_{N'}\} \\ 0 & \text{otherwise} \end{cases}
\end{aligned}$$

where the summation runs over all

$$m \in \langle j| = \{j_{1'}, j_{2'}, \dots, j_{N'}\}, \qquad \text{and} \qquad n \in |0\rangle = \{j_1, j_2, \dots, j_N\}.$$

The states $a_n\,|vac\rangle$ are Bloch functions, leading to $\sum_{\mathbf{k}\mathbf{k}'ss'} \langle \mathbf{k}'s'|\mathbf{H}|\mathbf{k}s\rangle\, (-1)^{d'+k+P}$. The sign $(-1)^{d'+k+P}$ disappears when forming the product: $\sum_{\mathbf{k}\mathbf{k}'ss'} \langle \mathbf{k}'s'|\mathbf{H}|\mathbf{k}s\rangle \langle \mathbf{k}s|\mathbf{H}|\mathbf{k}'s'\rangle$.

1.2. **Dipole-Dipole Interaction Term.** To compute the matrix elements

$$\langle \mathbf{k}s| \frac{\delta_{\alpha\beta} - 3\hat{\tilde{r}}_{il,\alpha}\hat{\tilde{r}}_{il,\beta}}{|\mathbf{R}_i - \mathbf{r}_l|^3} S_l^\beta |\mathbf{k}'s\rangle$$

we use the spinor-valued Bloch wavefunctions derived by Varela [3, 4], which we shall abbreviate as:

$$\boldsymbol{\psi}_{n,s}^{\nu,\zeta} = \frac{\sqrt{s}}{2} \begin{bmatrix} (se^{i\theta/2} + e^{-i\theta/2})e^{-i\varphi/2} \\ \zeta(se^{i\theta/2} - e^{-i\theta/2})e^{i\varphi/2} \end{bmatrix} e^{i\nu\tilde{n}\varphi} = \begin{bmatrix} F_A e^{-i\varphi/2} \\ \zeta F_B^* e^{i\varphi/2} \end{bmatrix} e^{i\nu\tilde{n}\varphi},$$

$$F_A = \frac{\sqrt{s}}{2}(se^{i\theta/2} + e^{-i\theta/2}), \qquad F_B = \frac{\sqrt{s}}{2}(se^{-i\theta/2} - e^{i\theta/2}).$$

The symbols we will need are:

- $s = \pm 1$ is the spin label
- $\nu = \pm 1$ is the electron propagation direction

- $\zeta = \pm 1$ labels the right- and left-handedness of the helix
- $\tilde{n} = n/2M\mathcal{N}$ labels the subbands corresponding to the discrete modes due to longitudinal confinement within a helix
- n is an integer in the interval $0 < n < M$
- M is the number of bases per turn.
- φ ranges from 0 to $2\pi\mathcal{N}$
- $\mathcal{N}$ is the number of turns in the helix.

Conversion from helicoidal to Cartesian coordinates is done using the mapping:

$$\varphi \mapsto (a\cos\varphi, a\sin\varphi, b\varphi), \varphi \in [0, T].$$

This leads to the substitutions:

$$\mathbf{R}_1 = (a\cos\varphi_1, a\sin\varphi_1, b\varphi_1)$$

$$\mathbf{R}_2 = (a\cos\varphi_2, a\sin\varphi_2, b\varphi_2)$$

$$\mathbf{r}_l = (a\cos\varphi_l, a\sin\varphi_l, b\varphi_l).$$

Note: $\varphi_l = (r_l)_z/b = z_l/b$. In helicoidal coordinates, integration over the spatial coordinates involves integrals of the type:

$$\frac{1}{2\pi}\int_0^{2\pi} e^{i[\nu(\tilde{n}'-\tilde{n})+\xi]\varphi_l}\left[(a\cos\varphi_1 - a\cos\varphi_l)^2 + (a\sin\varphi_1 - a\sin\varphi_l)^2 + (b\varphi_1 - b\varphi_l)^2\right]^{-3/2} d\varphi_l$$

where $\xi = -1, 0, 1$. According to Varela and Lopéz [3, 4], $a = 2$ nm, $b = 3.38$ nm, $\Delta\varphi = 36°$, $R = 0.70$ nm (interbase distance). The geometry is shown in Fig. 1.

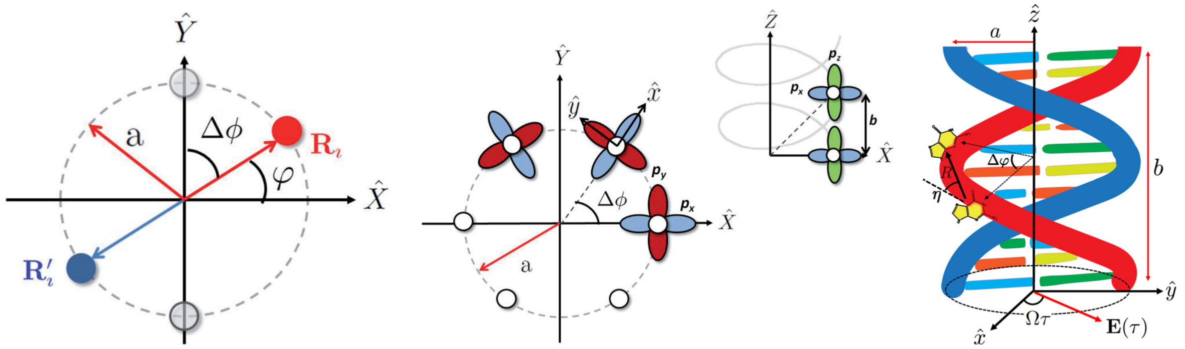

Supplementary Figure 1. Geometry of the helical DNA-like model used for computation of the electronic band structure for the CISS effect. Illustration reproduced from the work of Varela and López [3, 4].

The last term is the largest for two reasons: $b > a$ so $b^2 > a^2$ (by a factor of almost 3); also φ ranges from 0 to 2π whereas cos and sin range from -1 to 1, when $\mathcal{N} = 1$. That's a factor $((4\pi)/4)^2 \approx 9.9$. Therefore, it can be larger by almost 30×. We note that the integral of the DD tensor can be computed as:

$$\nabla_{\mathbf{R}_i}\nabla_{\mathbf{R}_i}\frac{1}{2\pi}\int_0^{2\pi} d\varphi_l \frac{e^{i[\nu(\tilde{n}'-\tilde{n})+\xi]\varphi_l}}{|\mathbf{R}_i - \mathbf{r}_l|}.$$

We therefore expand it:

$$\frac{1}{\sqrt{(a\cos\varphi_1 - a\cos\varphi_l)^2 + (a\sin\varphi_1 - a\sin\varphi_l)^2 + (b\varphi_1 - b\varphi_l)^2}} \approx \frac{1}{b|\varphi_1-\varphi_l|} - \frac{a^2}{2b^3|\varphi_1-\varphi_l|^3} + \frac{3a^4}{8b^5|\varphi_1-\varphi_l|^5} + O\left(a^6\right).$$

We shall take $M = 10$ and $\mathcal{N} = 1$. This gives:

$$\nabla_{\mathbf{R}_i}\nabla_{\mathbf{R}_i}\frac{1}{2\pi}\int_0^{2\pi} d\varphi_l e^{i[\nu(\tilde{n}'-\tilde{n})+\xi]\varphi_l}\left[\frac{1}{b|\varphi_1-\varphi_l|} - \frac{a^2}{2b^3|\varphi_1-\varphi_l|^3} + \frac{3a^4}{8b^5|\varphi_1-\varphi_l|^5} + O\left(a^6\right)\right].$$

Integrating the first term:

$$\lim_{\epsilon\to 0}\int_0^{2\pi} d\varphi_l \frac{e^{i[\nu(\tilde{n}'-\tilde{n})+\xi]\varphi_l}}{|\varphi_1-\varphi_l| - i\epsilon} = p.v.\int_0^{2\pi} d\varphi_l \frac{e^{i[\nu(\tilde{n}'-\tilde{n})+\xi]\varphi_l}}{|\varphi_1-\varphi_l|} + i\pi e^{i[\nu(\tilde{n}'-\tilde{n})+\xi]\varphi_1}$$

where $0 \le \varphi_1 \le 2\pi$. Where we made use of the Plemelj-Sokhotski theorem:

$$\lim_{\epsilon\to 0}\frac{1}{x\pm i\epsilon} = p.v.\frac{1}{x} \mp i\pi\delta(x).$$

Furthermore making use of the Cauchy principal value

$$p.v.\int_{-\infty}^{\infty}\frac{e^{ix}}{x}dx = i\pi, \qquad p.v.\int_{-\infty}^{\infty}\frac{e^{i(x-x_0)}}{x}dx = i\pi e^{ix_0},$$

we conclude that

$$\int_0^{2\pi} d\varphi_l \frac{e^{i[\nu(\tilde{n}'-\tilde{n})+\xi]\varphi_l}}{|\varphi_1-\varphi_l| - i0} = i\pi e^{i[\nu(\tilde{n}'-\tilde{n})+\xi]\varphi_1} + i\pi e^{i[\nu(\tilde{n}'-\tilde{n})+\xi]\varphi_1} = 2i\pi e^{i[\nu(\tilde{n}'-\tilde{n})+\xi]\varphi_1}.$$

We then take the gradient (twice). But we must make use of the chain rule: $\partial_{R_j} = \frac{\partial\varphi_1}{\partial R_j}\frac{\partial}{\partial\varphi_1}$, where

$$\frac{\partial x}{\partial\varphi_1} = -a\sin\varphi, \quad \frac{\partial y}{\partial\varphi_1} = a\cos\varphi, \quad \frac{\partial z}{\partial\varphi_1} = b.$$

Inverting

$$\frac{\partial\varphi_1}{\partial x} = -\frac{1}{a\sin\varphi}, \quad \frac{\partial\varphi_1}{\partial y} = \frac{1}{a\cos\varphi}, \quad \frac{\partial\varphi_1}{\partial z} = \frac{1}{b}.$$

The transformation is not 1-1. Therefore,

$$\nabla_{\mathbf{R}_1} = \left(-\frac{1}{a\sin\varphi_1}\partial_{\varphi_1}, \frac{1}{a\cos\varphi_1}\partial_{\varphi_1}, \frac{1}{b}\partial_{\varphi_1}\right).$$

The gradient is defined on $[0,2\pi]\setminus\{0,\pi/2,\pi,3\pi/2,2\pi\}$. Care will be needed around singularities. Taking the gradient once:

$$\nabla_{\mathbf{R}_1}\int_0^{2\pi} d\varphi_l \frac{e^{i[\nu(\tilde{n}'-\tilde{n})+\xi]\varphi_l}}{b|\varphi_1-\varphi_l|} = 2i\pi b^{-1}\nabla_{\mathbf{R}_1} e^{i[\nu(\tilde{n}'-\tilde{n})+\xi]\varphi_1}$$

$$= \frac{2\pi i}{b}\left(-\frac{1}{a\sin\varphi_1}, \frac{1}{a\cos\varphi_1}, \frac{1}{b}\right) i[\nu(\tilde{n}'-\tilde{n})+\xi] e^{i[\nu(\tilde{n}'-\tilde{n})+\xi]\varphi_1}.$$

Taking another gradient we obtain the matrix:

$$\nabla_{\mathbf{R}_1}\nabla_{\mathbf{R}_1}\int_0^{2\pi} d\varphi_l \frac{e^{i[\nu(\tilde{n}'-\tilde{n})+\xi]\varphi_l}}{b|\varphi_1-\varphi_l|} = -2\pi[\nu(\tilde{n}'-\tilde{n})+\xi]e^{i[\nu(\tilde{n}'-\tilde{n})+\xi]\varphi_1}b^{-1}$$
$$\times\begin{bmatrix} i\frac{1}{a^2\sin\varphi_1}\csc(\varphi_1)([\nu(\tilde{n}'-\tilde{n})+\xi]+i\cot(\varphi_1)) & -\frac{1}{a^2\sin\varphi_1}\sec(\varphi_1)(\tan(\varphi_1)+i[\nu(\tilde{n}'-\tilde{n})+\xi]) & i\frac{[\nu(\tilde{n}'-\tilde{n})+\xi]}{a^2\sin\varphi_1} \\ -i\frac{1}{a^2\cos\varphi_1}\csc(\varphi_1)([\nu(\tilde{n}'-\tilde{n})+\xi]+i\cot(\varphi_1)) & \frac{1}{a^2\cos\varphi_1}\sec(\varphi_1)(\tan(\varphi_1)+i[\nu(\tilde{n}'-\tilde{n})+\xi]) & -i\frac{[\nu(\tilde{n}'-\tilde{n})+\xi]}{a^2\cos\varphi_1} \\ -i\frac{1}{b^2}\csc(\varphi_1)([\nu(\tilde{n}'-\tilde{n})+\xi]-i\cot(\varphi_1)) & -\frac{1}{b^2}\sec(\varphi_1)(\tan(\varphi_1)+i[\nu(\tilde{n}'-\tilde{n})+\xi]) & -i\frac{[\nu(\tilde{n}'-\tilde{n})+\xi]}{b^2} \end{bmatrix}.$$

In fact, we need matrix elements of the form

$$\nabla_{\mathbf{R}_i}\nabla_{\mathbf{R}_i}\frac{1}{2\pi}\int_0^{2\pi} d\varphi_l \frac{e^{-i[\nu(\tilde{n}'-\tilde{n})+\xi]\varphi_l}}{|\mathbf{R}_i-\mathbf{r}_l|}\langle\tilde{n}'|S_l^\beta|\tilde{n}\rangle\,,$$

where $\beta = x, y, z$. Using the matrix elements of Pauli matrices $\langle\boldsymbol{\psi}^{\nu,\zeta}_{n',s}|\sigma_i|\boldsymbol{\psi}^{\nu,\zeta}_{n,s}\rangle$, $i = x, y, z$, we obtain:

$$M_{1,x}(\tilde{n}',\tilde{n}) \equiv 2\pi b^{-1}\zeta F_A^* F_B^*[\nu(\tilde{n}'-\tilde{n})+1]e^{i[\nu(\tilde{n}'-\tilde{n})+1]\varphi_1}$$
$$\times\begin{bmatrix} i\frac{1}{a^2\sin\varphi_1}\csc(\varphi_1)([\nu(\tilde{n}'-\tilde{n})+1]+i\cot(\varphi_1)) & -\frac{1}{a^2\sin\varphi_1}\sec(\varphi_1)(\tan(\varphi_1)+i[\nu(\tilde{n}'-\tilde{n})+1]) & i\frac{[\nu(\tilde{n}'-\tilde{n})+1]}{a^2\sin\varphi_1} \\ -i\frac{1}{a^2\cos\varphi_1}\csc(\varphi_1)([\nu(\tilde{n}'-\tilde{n})+1]+i\cot(\varphi_1)) & \frac{1}{a^2\cos\varphi_1}\sec(\varphi_1)(\tan(\varphi_1)+i[\nu(\tilde{n}'-\tilde{n})+1]) & -i\frac{[\nu(\tilde{n}'-\tilde{n})+1]}{a^2\cos\varphi_1} \\ i\frac{1}{a^2}\csc(\varphi_1)([\nu(\tilde{n}'-\tilde{n})+1]+i\cot(\varphi_1)) & -\frac{1}{a^2}\sec(\varphi_1)(\tan(\varphi_1)+i[\nu(\tilde{n}'-\tilde{n})+1]) & i\frac{[\nu(\tilde{n}'-\tilde{n})+1]}{a^2} \end{bmatrix}$$
$$+\,2\pi\zeta b^{-1}F_A F_B[\nu(\tilde{n}'-\tilde{n})-1]e^{i[\nu(\tilde{n}'-\tilde{n})-1]\varphi_1}$$
$$\times\begin{bmatrix} i\frac{1}{a^2\sin\varphi_1}\csc(\varphi_1)([\nu(\tilde{n}'-\tilde{n})-1]+i\cot(\varphi_1)) & -\frac{1}{a^2\sin\varphi_1}\sec(\varphi_1)(\tan(\varphi_1)+i[\nu(\tilde{n}'-\tilde{n})-1]) & i\frac{[\nu(\tilde{n}'-\tilde{n})-1]}{a^2\sin\varphi_1} \\ -i\frac{1}{a^2\cos\varphi_1}\csc(\varphi_1)([\nu(\tilde{n}'-\tilde{n})-1]+i\cot(\varphi_1)) & \frac{1}{a^2\cos\varphi_1}\sec(\varphi_1)(\tan(\varphi_l)+i[\nu(\tilde{n}'-\tilde{n})-1]) & -i\frac{[\nu(\tilde{n}'-\tilde{n})-1]}{a^2\cos\varphi_1} \\ -i\frac{1}{b^2}\csc(\varphi_1)([\nu(\tilde{n}'-\tilde{n})-1]+i\cot(\varphi_1)) & \frac{1}{b^2}\sec(\varphi_1)(\tan(\varphi_1)+i[\nu(\tilde{n}'-\tilde{n})-1]) & -i\frac{[\nu(\tilde{n}'-\tilde{n})-1]}{b^2} \end{bmatrix} \tag{2}$$

$$M_{1,y}(\tilde{n}',\tilde{n}) \equiv -2\pi i\zeta b^{-1}F_A^* F_B^*[\nu(\tilde{n}'-\tilde{n})+1]e^{i[\nu(\tilde{n}'-\tilde{n})+1]\varphi_1}$$
$$\times\begin{bmatrix} i\frac{1}{a^2\sin\varphi_1}\csc(\varphi_1)([\nu(\tilde{n}'-\tilde{n})+1]+i\cot(\varphi_1)) & -\frac{1}{a^2\sin\varphi_1}\sec(\varphi_1)(\tan(\varphi_1)+i[\nu(\tilde{n}'-\tilde{n})+1]) & i\frac{[\nu(\tilde{n}'-\tilde{n})+1]}{a^2\sin\varphi_1} \\ -i\frac{1}{a^2\cos\varphi_1}\csc(\varphi_1)([\nu(\tilde{n}'-\tilde{n})+1]+i\cot(\varphi_1)) & \frac{1}{a^2\cos\varphi_1}\sec(\varphi_1)(\tan(\varphi_1)+i[\nu(\tilde{n}'-\tilde{n})+1]) & -i\frac{[\nu(\tilde{n}'-\tilde{n})+1]}{a^2\cos\varphi_1} \\ i\frac{1}{a^2}\csc(\varphi_1)([\nu(\tilde{n}'-\tilde{n})+1]+i\cot(\varphi_1)) & -\frac{1}{a^2}\sec(\varphi_1)(\tan(\varphi_1)+i[\nu(\tilde{n}'-\tilde{n})+1]) & i\frac{[\nu(\tilde{n}'-\tilde{n})+1]}{a^2} \end{bmatrix}$$
$$+\,2\pi i\zeta b^{-1}F_A F_B[\nu(\tilde{n}'-\tilde{n})-1]e^{i[\nu(\tilde{n}'-\tilde{n})-1]\varphi_1}$$
$$\times\begin{bmatrix} i\frac{1}{a^2\sin\varphi_1}\csc(\varphi_1)([\nu(\tilde{n}'-\tilde{n})-1]+i\cot(\varphi_1)) & -\frac{1}{a^2\sin\varphi_1}\sec(\varphi_1)(\tan(\varphi_1)+i[\nu(\tilde{n}'-\tilde{n})-1]) & i\frac{[\nu(\tilde{n}'-\tilde{n})-1]}{a^2\sin\varphi_1} \\ -i\frac{1}{a^2\cos\varphi_1}\csc(\varphi_1)([\nu(\tilde{n}'-\tilde{n})-1]+i\cot(\varphi_1)) & \frac{1}{a^2\cos\varphi_1}\sec(\varphi_1)(\tan(\varphi_1)+i[\nu(\tilde{n}'-\tilde{n})-1]) & -i\frac{[\nu(\tilde{n}'-\tilde{n})-1]}{a^2\cos\varphi_1} \\ -i\frac{1}{b^2}\csc(\varphi_1)([\nu(\tilde{n}'-\tilde{n})-1]-i\cot(\varphi_1)) & -\frac{1}{b^2}\sec(\varphi_1)(\tan(\varphi_1)+i[\nu(\tilde{n}'-\tilde{n})-1]) & -i\frac{[\nu(\tilde{n}'-\tilde{n})-1]}{b^2} \end{bmatrix} \tag{3}$$

$$M_{1,z}(\tilde{n}',\tilde{n}) \equiv 2\pi b^{-1}(|F_A|^2-\zeta^2|F_B|^2)\nu(\tilde{n}-\tilde{n}')e^{i\nu(\tilde{n}-\tilde{n}')\varphi_1}$$
$$\times\begin{bmatrix} i\frac{1}{a^2\sin\varphi_1}\csc(\varphi_1)(\nu(\tilde{n}-\tilde{n}')+i\cot(\varphi_1)) & -\frac{1}{a^2\sin\varphi_1}\sec(\varphi_1)(\tan(\varphi_1)+i\nu(\tilde{n}-\tilde{n}')) & i\frac{\nu(\tilde{n}-\tilde{n}')}{a^2\sin\varphi_1} \\ -i\frac{1}{a^2\cos\varphi_1}\csc(\varphi_1)(\nu(\tilde{n}-\tilde{n}')+i\cot(\varphi_1)) & \frac{1}{a^2\cos\varphi_1}\sec(\varphi_1)(\tan(\varphi_1)+i\nu(\tilde{n}-\tilde{n}')) & -i\frac{\nu(\tilde{n}-\tilde{n}')}{a^2\cos\varphi_1} \\ -i\frac{1}{b^2}\csc(\varphi_1)(\nu(\tilde{n}-\tilde{n}')+i\cot(\varphi_1)) & -\frac{1}{b^2}\sec(\varphi_1)(\tan(\varphi_1)+i\nu(\tilde{n}-\tilde{n})) & -i\frac{\nu(\tilde{n}-\tilde{n}')}{b^2} \end{bmatrix} \tag{4}$$

and similarly for $M_{2,x}$, $M_{2,y}$ and $M_{2,z}$. For the energy denominator of our perturbation expression, we start from the linear energy dispersion relation (see Eq. 109, Ref. [3]):

$$E^{\nu,\zeta}_{n,s} = |T|\tilde{n} - s\nu\frac{\sqrt{T^2+(4\lambda^{in}_{SO})^2}}{2}$$

with $\tilde{n} = n/2M\mathcal{N}$ according to Ref. [3]. In their follow-up work, Ref. [4], the quantity that multiplies $|T|$ is n, not $\tilde{n}$ (see Eq. 13 in Ref. [4]). The spin dependence of the denominator introduces degeneracies in the system, which manifest themselves as singularities in the non-degenerate perturbation expansion. To circumvent this, one may justify the approximation $E^{\nu,\zeta}_{n,s} \approx |T|n$ at large n. Secondly, we may invoke degenerate perturbation theory, which excludes singular terms from the perturbation summations. On a practical level, some singularities can be excluded from the denominator by taking $s = s'$, which could be justified physically based on the spin-momentum locking property of electron transport in CISS, which ultimately originates from the spin-orbit coupling interaction. This leads to:

$$(5) \qquad \mathcal{H}^{DD}_{eff} = \left(\frac{\mu_0}{4\pi}\right)^2 \gamma_I^2\gamma_S^2 \sum_{n,n'}\sum_{\alpha,\beta}\sum_{\alpha',\beta'} I_1^\alpha \frac{M^{\alpha\beta}_{1,\beta}(\tilde{n}',\tilde{n})M^{\alpha'\beta'}_{2,\beta'}(\tilde{n},\tilde{n}')}{|T|(n'-n)} I_2^{\alpha'} f(\tilde{n})[1-f(\tilde{n}')] + c.c.$$

We will take the limit of low temperature. There are many terms. All can be obtained analytically in closed form and pose no difficulty. We will therefore not work out all terms except to illustrate the fate of different functional forms encountered. Setting $\xi = 0$ for simplicity, we find terms of the form

$$\sum_{n,n'=0}^{M} (\tilde{n}-\tilde{n}')e^{i\nu(\tilde{n}-\tilde{n}')\varphi_1}e^{i\nu(\tilde{n}-\tilde{n}')\varphi_2} f(\tilde{n})[1-f(\tilde{n}')]\frac{1}{\tilde{n}-\tilde{n}'},$$

which, in the low temperature limit, becomes

$$= \sum_{\tilde{n}=0}^{\tilde{n}_F}\sum_{\tilde{n}'=\tilde{n}_F}^{M} e^{i\nu(\tilde{n}-\tilde{n}')(\varphi_1-\varphi_2)} = \frac{e^{-\frac{i(\tilde{n}_F-1)\nu(\varphi_1-\varphi_2)}{2M\mathcal{N}}}\left(e^{\frac{i(\tilde{n}_F+1)\nu(\varphi_1-\varphi_2)}{2M\mathcal{N}}}-1\right)\left(e^{\frac{i\nu(\varphi_1-\varphi_2)(\tilde{n}_F-M-1)}{2M\mathcal{N}}}-1\right)}{\left(e^{\frac{i\nu(\varphi_1-\varphi_2)}{2M\mathcal{N}}}-1\right)^2}.$$

This term does not become singular unless $\varphi_1 = \varphi_2$ (two localized spins at the same exact position), which goes against the idea of coupling two distinct spins localized in different positions.

Also, we have terms of the type:

$$\sum_{n,n'=0}^{M} (\tilde{n}-\tilde{n}')^2 e^{i\nu(\tilde{n}-\tilde{n}')\varphi_1}e^{i\nu(\tilde{n}-\tilde{n}')\varphi_2} f(\tilde{n})[1-f(\tilde{n}')]\frac{1}{\tilde{n}-\tilde{n}'} = \sum_{\tilde{n}=0}^{\tilde{n}_F}\sum_{\tilde{n}'=\tilde{n}_F}^{M}(\tilde{n}-\tilde{n}')e^{i\nu(\tilde{n}-\tilde{n}')(\varphi_1-\varphi_2)}$$

which can be obtained by differentiating the previous expression with respect to $(\varphi_1 - \varphi_2)$ and dividing by $i\nu$. The result is:

$$= \frac{e^{-\frac{i(\tilde{n}_F-1)\nu(\varphi_1-\varphi_2)}{2M\mathcal{N}}}}{2M\mathcal{N}\left(e^{\frac{i\nu(\varphi_1-\varphi_2)}{2M\mathcal{N}}}-1\right)^3}\Big(-2e^{\frac{i(\tilde{n}_F+1)\nu(\varphi_1-\varphi_2)}{2M\mathcal{N}}}+(\tilde{n}_F+1)e^{\frac{i\nu(\varphi_1-\varphi_2)}{2M\mathcal{N}}}+(\tilde{n}_F-M+1)e^{\frac{i\nu(\varphi_1-\varphi_2)(2\tilde{n}_F-M)}{2M\mathcal{N}}}$$

$$+Me^{\frac{i\nu(\varphi_1-\varphi_2)(\tilde{n}_F-M-1)}{2M\mathcal{N}}} - (M+2)e^{\frac{i\nu(\varphi_1-\varphi_2)(\tilde{n}_F-M)}{2M\mathcal{N}}} + (M+1-\tilde{n}_F)e^{\frac{i\nu(\varphi_1-\varphi_2)(2\tilde{n}_F-M+1)}{2M\mathcal{N}}} - \tilde{n}_F + 1\Big).$$

1.2.1. *Enantiospecific NMR Response.* It should now be clear that the effective dipole-dipole coupling tensor is enantiospecific upon inspection of Eq. (5), which contains a product

$M^{\alpha\beta}_{1,\beta}(\tilde{n}',\tilde{n})M^{\alpha'\beta'}_{2,\beta'}(\tilde{n},\tilde{n}')$. Explicitly, this term is:

$$\sum_{\beta,\beta'} M^{\alpha\beta}_{1,\beta}(\tilde{n}',\tilde{n})M^{\alpha'\beta'}_{2,\beta'}(\tilde{n},\tilde{n}') = \left[M^{\alpha,1}_{1,x}(\tilde{n}',\tilde{n}) + M^{\alpha,2}_{1,y}(\tilde{n}',\tilde{n}) + M^{\alpha,3}_{1,z}(\tilde{n}',\tilde{n})\right] \times \left[M^{\alpha',1}_{2,x}(\tilde{n},\tilde{n}') + M^{\alpha',2}_{2,y}(\tilde{n},\tilde{n}') + M^{\alpha',3}_{2,z}(\tilde{n},\tilde{n}')\right].$$

While $M_{i,z}$ is independent of ζ, both $M_{i,x}$ and $M_{i,y}$ depend linearly on ζ. The tensor (indices α,α') contains terms such as $M_{1,z}M_{2,x}$, which depend linearly on ζ. The effect of enantiomer handedness is to flip the sign of this term, leading to a change in the magnitude of the dipole-dipole interaction. The term $M^{\alpha,3}_{1,z}(\tilde{n}',\tilde{n})M^{\alpha',3}_{2,z}(\tilde{n},\tilde{n}')$ does not depend on ζ, since neither factor depend on ζ. Neither do $M^{\alpha,1}_{1,x}(\tilde{n}',\tilde{n})M^{\alpha',1}_{2,x}(\tilde{n},\tilde{n}')$ and $M^{\alpha,2}_{1,y}(\tilde{n}',\tilde{n})M^{\alpha',2}_{2,y}(\tilde{n},\tilde{n}')$ since $\zeta^2 = 1$.

1.2.2. *Order of magnitude estimate.* From this equation:

$$\mathcal{H}^{DD}_{eff} = \left(\frac{\mu_0}{4\pi}\right)^2 \gamma_I^2\gamma_S^2\hbar^4 \sum_{n,n'}\sum_{\alpha,\beta}\sum_{\alpha',\beta'} I_1^\alpha \frac{M^{\alpha\beta}_{1,\beta}(\tilde{n}',\tilde{n})M^{\alpha'\beta'}_{2,\beta'}(\tilde{n},\tilde{n}')}{|T|(n'-n)} I_2^{\alpha'} f(\tilde{n})[1-f(\tilde{n}')] + c.c.$$

Taking a typical term from the matrix M the magnitude is

$$\left|\mathcal{H}^{DD}_{eff}\right| \sim \left(\frac{\mu_0}{4\pi}\right)^2 \gamma_I^2\gamma_S^2\hbar^4 \sum_{\alpha,\beta}\sum_{\alpha',\beta'} \left|\frac{(2\pi(|F_A|^2 - \zeta^2|F_B|^2))^2}{|T|}\right| \frac{1}{b^2a^4}$$

where $|F_A|, |F_B| \leq 1$. So,

$$\left|\mathcal{H}^{DD}_{eff}\right| \sim \left(\frac{\mu_0}{4\pi}\right)^2 \gamma_I^2\gamma_S^2\hbar^4 16 \left|\frac{(4\pi)^2}{T(2M\mathcal{N})}\right| \frac{1}{b^2a^4} \sim (\mu_0)^2\, \gamma_I^2\gamma_S^2\hbar^4 16 \left|\frac{1}{T(2M\mathcal{N})}\right| \frac{1}{b^2a^4} \sim \frac{2\mu_0^2\gamma_I^2\gamma_S^2\hbar^4}{|T|(2M\mathcal{N})}\frac{1}{b^2a^4}.$$

According to Varela, $T = 2t_{in}$, $t_{in} = -10$ meV $= 1.6\times10^{-21}$ J. Plugging numbers:

$$\left|\mathcal{H}^{DD}_{eff}\right| \sim \frac{2\mu_0^2(\gamma_I\hbar)^2(\gamma_S\hbar)^2}{|T|(2M\mathcal{N})}\frac{1}{b^2a^4}$$

$$\approx \frac{2(1.257\times10^{-6}\mathrm{N}\cdot\mathrm{A}^{-2})^2(-1.76\times10^{11}\,\mathrm{rad{\cdot}s^{-1}{\cdot}T^{-1}})^2(267.5\times10^6\,\mathrm{rad{\cdot}s^{-1}{\cdot}T^{-1}})^2}{(1.6\times10^{-21}\ \mathrm{J})(2\times10^{-9}\ \mathrm{m})^2(3.38\times10^{-9}\ \mathrm{m})^4(2M\mathcal{N})}\cdot(1.05\times10^{-34}\ \mathrm{J.s})^4$$

$$\approx \frac{1.02\times10^{-36}\ \mathrm{J}}{2M\mathcal{N}} \approx \frac{0.01\ \mathrm{Hz}}{2M\mathcal{N}}.$$

The reader can verify that the units are Joules. But 0.01 Hz$/(2M\mathcal{N})$ is not the full story. We also have multiplicative factors of the form:

$$\frac{e^{-\frac{i(\tilde{n}_F-1)\nu(\varphi_1-\varphi_2)}{2M\mathcal{N}}}\left(e^{\frac{i(\tilde{n}_F+1)\nu(\varphi_1-\varphi_2)}{2M\mathcal{N}}}-1\right)\left(e^{\frac{i\nu(\varphi_1-\varphi_2)(\tilde{n}_F-M-1)}{2M\mathcal{N}}}-1\right)}{\left(e^{\frac{i\nu(\varphi_1-\varphi_2)}{2M\mathcal{N}}}-1\right)^2},$$

which blow up when $|\varphi_1-\varphi_2|/(2M\mathcal{N})$ is an integer multiple of 2π, i.e. when $|\varphi_1-\varphi_2| = 4\pi M\mathcal{N}m$, $m\in\mathbb{N}$. We note that when the denominator blows up the numerator does not, because it depends on the Fermi energy $\tilde{n}_F$. As to the denominator, we mentioned earlier that it blows up when $\varphi_1 = \varphi_2$, a situation that is not allowed (or worst, when $M\mathcal{N}\to\infty$).

Finally, we also have factors of $\tan(\varphi_1)$, $\csc(\varphi_1)$, $\cot(\varphi_1)$ and $\sec(\varphi_1)$, all of which can "blow up" depending on the argument to the function (φ_1 or φ_2 here). For example, tan

and sec blow up at $(n+\frac{1}{2})\pi$, $n \in \mathbb{Z}$ whereas csc and cot blow up at $n\pi$. Recall that $\csc(\varphi_1) = 1/\sin(\varphi_1)$ and $\sec(\varphi_1) = 1/\cos(\varphi_1)$. Some of the terms are:

1) $\sec(\varphi_1)\tan(\varphi_1)/\sin(\varphi_1) = \sec^2(\varphi_1)$. Similar to sec, this functions blows up at the points $(n+\frac{1}{2})\pi$.

2) $\csc(\varphi_1)/\sin(\varphi_1) = \csc^2(\varphi_1)$, which blows up at $n\pi/2$.

3) $\sec(\varphi_1)/\sin(\varphi_1) = \csc(\varphi_1)\sec(\varphi_1)$, which blows up at $(n+\frac{1}{2})\pi$.

4) $\csc(\varphi_1)\cot(\varphi_1)/\sin(\varphi_1) = \csc^2(\varphi_1)\cot(\varphi_1)$, which blows up at the points $n\pi$. We rule out this scenario, as the two nuclear spins are not likely to be located at the center of the molecule.

Let's plot one such matrix element. Let's take the upper left hand corner element. Recall that $\tilde{n} = n/2M\mathcal{N}$ and $0 \le n \le M$. Let's choose Fermi level to be in the middle ($n = M/2$ so that $\tilde{n} = 1/4\mathcal{N}$, so that the levels are half-filled). We will stick with $\mathcal{N} = 1$, $M = 10$ so that $\tilde{n}_F = 1/40$. The amplitude of the $I_1^z \mathsf{F}_{zz} I_2^z$ term is plotted as a function of $\varphi_1, \varphi_2 \in [0, 2\pi]$ in Fig. 2a of the main text.

1.3. **Fermi Contact Term.** The Fermi contact term:

$$\mathcal{H}_{eff}^{FC} = \left(\frac{2\mu_0}{3}\right)^2 \gamma_I^2\gamma_S^2\hbar^4 \sum_{\mathbf{k},s}\sum_{\mathbf{k}',s'} \mathbf{I}_1 \cdot \frac{\langle \mathbf{k}s| \sum_l \mathbf{S}_l \delta^{(3)}(\mathbf{r}_l - \mathbf{R}_1)|\mathbf{k}'s'\rangle \langle \mathbf{k}'s'| \sum_l \mathbf{S}_l \delta^{(3)}(\mathbf{r}_l - \mathbf{R}_2)|\mathbf{k}s\rangle}{E_{\mathbf{k}s} - E_{\mathbf{k}'s'}} \cdot \mathbf{I}_2 + c.c.$$

involves computing integrals of the form

$$\langle \tilde{n}'s| \sum_l \mathbf{S}_l \delta^{(3)}(\mathbf{r}_l - \mathbf{R}_1)|\tilde{n}s\rangle \equiv \langle \boldsymbol{\psi}_{n',s}^{\nu,\zeta}| \sum_l \mathbf{S}_l \delta^{(3)}(\mathbf{r}_l - \mathbf{R}_1)|\boldsymbol{\psi}_{n,s}^{\nu,\zeta}\rangle$$

using the Varela spinors

$$\boldsymbol{\psi}_{n,s}^{\nu,\zeta} = \begin{bmatrix} F_A e^{-i\varphi/2} \\ \zeta F_B^* e^{i\varphi/2} \end{bmatrix} e^{i\nu\tilde{n}\varphi}, \quad F_A = \frac{\sqrt{s}}{2}(se^{i\theta/2} + e^{-i\theta/2}), \quad F_B = \frac{\sqrt{s}}{2}(se^{-i\theta/2} - e^{i\theta/2}).$$

There are three such terms, one for S_x, S_y and S_z:

$$\langle \boldsymbol{\psi}_{n',s}^{\nu,\zeta}| \sum_l \frac{\sigma_x}{2}\delta^{(3)}(\mathbf{r}_l - \mathbf{R}_1)|\boldsymbol{\psi}_{n,s}^{\nu,\zeta}\rangle, \ \langle \boldsymbol{\psi}_{n',s}^{\nu,\zeta}| \sum_l \frac{\sigma_y}{2}\delta^{(3)}(\mathbf{r}_l - \mathbf{R}_1)|\boldsymbol{\psi}_{n,s}^{\nu,\zeta}\rangle\rangle, \ \langle \boldsymbol{\psi}_{n',s}^{\nu,\zeta}| \sum_l \frac{\sigma_z}{2}\delta^{(3)}(\mathbf{r}_l - \mathbf{R}_1)|\boldsymbol{\psi}_{n,s}^{\nu,\zeta}\rangle.$$

We may temporarily denote these terms as s_x, s_y and s_z, respectively. See Section 1.4.2 for their computation. They give rise to an effective spin-spin coupling tensor:

$$\begin{bmatrix} s_x s_x & s_x s_y & s_x s_z \\ s_y s_x & s_y s_y & s_y s_z \\ s_z s_x & s_z s_y & s_z s_z \end{bmatrix} = \ldots$$

Taking care of ensuring that the s on the left involves a braket of the form $\langle n'| \cdot |n\rangle$ while the s on the right is of the form $\langle n| \cdot |n'\rangle$. Because the Fermi contact term does not contain any singularities we expect that its magnitude may be too weak for cross-polarization. For indirect electron-electron coupling, we gain a factor 10^6 from the stronger gyromagnetic ratios.

Our Fermi contact contribution becomes

$$(6) \qquad \mathcal{H}_{eff}^{FC} = \left(\frac{2\mu_0}{3}\right)^2 \frac{4^2\gamma_I^2\gamma_S^2\hbar^4}{(2\pi s)a^4b^2|\varphi_1||\varphi_2|} \sum_{n,n'} \mathbf{I}_1 \cdot \frac{\mathsf{F}_c(\tilde{n}',\tilde{n})}{|T|(n'-n)} \cdot \mathbf{I}_2 f(\tilde{n})[1 - f(\tilde{n}')] + c.c.$$

where the Fermi-contact coupling tensor $\mathsf{F}_c(\tilde{n}', \tilde{n})$ is given by the outer product (dyadic):

$$\mathsf{F}_c(\tilde{n}', \tilde{n}) = \begin{bmatrix} \zeta(F_A^* F_B^* e^{i\varphi_1} + F_A F_B e^{-i\varphi_1}) e^{-i\nu(\tilde{n}'-\tilde{n})\varphi_1} \\ i\zeta(F_A F_B e^{-i\varphi_1} - F_A^* F_B^* e^{i\varphi_1}) e^{-i\nu(\tilde{n}'-\tilde{n})\varphi_1} \\ (|F_A|^2 - |F_B|^2) e^{-i\nu(\tilde{n}'-\tilde{n})\varphi_1} \end{bmatrix} \otimes \begin{bmatrix} \zeta(F_A^* F_B^* e^{i\varphi_2} + F_A F_B e^{-i\varphi_2}) e^{-i\nu(\tilde{n}-\tilde{n}')\varphi_2} \\ i\zeta(F_A F_B e^{-i\varphi_2} - F_A^* F_B^* e^{i\varphi_2}) e^{-i\nu(\tilde{n}-\tilde{n}')\varphi_2} \\ (|F_A|^2 - |F_B|^2) e^{-i\nu(\tilde{n}-\tilde{n}')\varphi_2} \end{bmatrix}^T.$$

Summation over n, n', i.e. $\mathsf{F} \equiv \sum_{n,n'} \mathsf{F}_c(\tilde{n}', \tilde{n}) f(\tilde{n})[1 - f(\tilde{n}')]$, is straightforward, as explained previously. This yields the tensor components F_{xx}, F_{xy}, F_{xz}, F_{yx}, F_{yy}, F_{yz}, F_{zx}, F_{zy} and F_{zz}.

In high-field NMR the coefficient of the $I_z^1 I_z^2$ term, F_{zz} matters most. Here, F_{zz} does not depend on ζ. Consequently, in high-field NMR we do not expect the Fermi contact contribution to the indirect coupling to be enantioselective for this DNA toy model. We note that along the line $\varphi_1 = \varphi_2$ we have 0 times ∞, which is undefined. This is not a problem since $\varphi_1 \neq \varphi_2$ is a requirement. The interaction strength is plotted as a function of $\varphi_1, \varphi_2 \in [0, 2\pi]$ in Fig. 2b of the main text. Outside of the $\varphi_1 = \varphi_2$ region ($\varphi_1 \neq \varphi_2$) the magnitude is at most 10, which is a rather weak mechanism for CP. We note that the factor $\frac{1}{|\varphi_1||\varphi_2|}$ in Eq. (6) could in principle "blow up" if $|\varphi_1| = 0$, $|\varphi_2| = 0$ or both. Firstly, we cannot have both nuclear spins in the same location ($|\varphi_1| = |\varphi_2| = 0$). Also, even if one spin is close to the edge of the molecule, it is unlikely to be exactly at the position $|\varphi_1| = 0$ since the electronic wavefunction always extends beyond the nucleus. The distance of closest approach would be bounded from below by (at least) the Bohr radius ($\sim 0.5 \times 10^{-10}$ m). For a helix pitch of 3.38 nm, this would correspond to a lower bound of ~ 0.1 radians, which is still at least 6 orders of magnitude weaker than the dipole interaction contribution.

1.4. **Is an Applied Current Needed to Drive this Effective Interaction?** Intuitively, a current is needed to mediate the coupling between two localized spins. One may ask whether or not a voltage must be applied across the molecule. In quantum mechanics we may use the concept of probability current where an electron in state $\psi(x)$ gives rise to a current density

$$\vec{j}(x) = -\frac{e\hbar}{2m_e i}(\psi^*(x)\nabla\psi(x) - \psi(x)\nabla\psi^*(x)).$$

For spinors, the expression is

$$\vec{j}(x) = -\frac{e\hbar}{2m_e i}(\boldsymbol{\psi}^\dagger \nabla \boldsymbol{\psi} - (\nabla \boldsymbol{\psi}^\dagger)\boldsymbol{\psi}).$$

In order to compute this current, we need the derivative operator along the arc length of the curve. The metric tensor in local coordinates for the parametrized curve $\varphi \mapsto (a\cos\varphi, a\sin\varphi, b\varphi), \varphi \in [0, T]$ is:

$$g_{\varphi,\varphi} = \langle \frac{\partial}{\partial\varphi}, \frac{\partial}{\partial\varphi} \rangle = (-a\sin\varphi, a\cos\varphi, b)\cdot(-a\sin\varphi, a\cos\varphi, b) = a^2\sin^2\varphi + a^2\cos^2\varphi + b^2 = a^2 + b^2.$$

Denoting the length by ℓ, the element of length is:

$$d\ell^2 = g_{\varphi,\varphi} d\varphi \otimes d\varphi = (a^2 + b^2) d\varphi^2, \qquad d\ell = \sqrt{a^2 + b^2} d\varphi.$$

We will also need to make a slight adjustment to the Varela spinors, which are defined over the interval $[0, 2\pi]$, and do not have units of inverse square root length. Normally, quantum mechanical wavefunctions $|\psi(x)|$ have dimensions of $1/\sqrt{x}$ and we have the normalization

$\int_0^L |\psi(x)|^2 dx = 1$ whereas here we have $\frac{1}{2\pi}\int_0^{2\pi} |\psi(\varphi)|^2 d\varphi = 1$. Consider the following mapping and associated change of variables:

$$\varphi \mapsto \ell = \sqrt{a^2+b^2}\varphi, \qquad \varphi = \frac{\ell}{\sqrt{a^2+b^2}}, \qquad d\varphi = \frac{d\ell}{\sqrt{a^2+b^2}}.$$

The integral becomes:

$$\frac{1}{2\pi}\int_0^{2\pi\sqrt{a^2+b^2}} \frac{1}{\sqrt{a^2+b^2}} |\psi(\frac{\ell}{\sqrt{a^2+b^2}})|^2 d\ell = 1.$$

or (with $L = 2\pi\sqrt{a^2+b^2}$):

$$\int_0^{2\pi\sqrt{a^2+b^2}} \left(\frac{\left|\psi\left(\frac{\ell}{\sqrt{a^2+b^2}}\right)\right|^2}{2\pi\sqrt{a^2+b^2}} \right) d\ell = 1.$$

This means that the wavefunction with proper units is obtained by rescaling:

$$\psi \mapsto \frac{\psi\left(\frac{\ell}{\sqrt{a^2+b^2}}\right)}{\sqrt{2\pi}(a^2+b^2)^{1/4}}.$$

Taking the derivative of the spinor:

$$\boldsymbol{\psi}_{n,s}^{\nu,\zeta} = \begin{bmatrix} F_A e^{-i\varphi/2} \\ \zeta F_B^* e^{i\varphi/2} \end{bmatrix} e^{i\nu\tilde{n}\varphi},$$

we get:

$$\nabla\boldsymbol{\psi} = \frac{d}{d\ell}\frac{\boldsymbol{\psi}(\frac{\ell}{\sqrt{a^2+b^2}})}{(a^2+b^2)^{1/4}} = \frac{i}{\sqrt{a^2+b^2}(a^2+b^2)^{1/4}} \begin{bmatrix} F_A e^{-i\varphi/2}(\nu\tilde{n} - \frac{1}{2}) \\ \zeta F_B^* e^{i\varphi/2}(\nu\tilde{n} + \frac{1}{2}) \end{bmatrix} e^{i\nu\tilde{n}\varphi}$$

and

$$(\nabla\boldsymbol{\psi}^\dagger) = \frac{-i}{\sqrt{a^2+b^2}(a^2+b^2)^{1/4}} \begin{bmatrix} F_A^* e^{i\varphi/2}(\nu\tilde{n} - \frac{1}{2}) & \zeta F_B e^{-i\varphi/2}(\nu\tilde{n} + \frac{1}{2}) \end{bmatrix} e^{-i\nu\tilde{n}\varphi}.$$

Computing the products of spinors:

$$\boldsymbol{\psi}^\dagger\nabla\boldsymbol{\psi} = \begin{bmatrix} F_A^* e^{i\varphi/2} & \zeta F_B e^{-i\varphi/2} \end{bmatrix} e^{-i\nu\tilde{n}\varphi} \frac{i}{\sqrt{a^2+b^2}(a^2+b^2)^{1/2}} \begin{bmatrix} F_A e^{-i\varphi/2}(\nu\tilde{n} - \frac{1}{2}) \\ \zeta F_B^* e^{i\varphi/2}(\nu\tilde{n} + \frac{1}{2}) \end{bmatrix} e^{i\nu\tilde{n}\varphi}$$

$$= \frac{i}{\sqrt{a^2+b^2}(a^2+b^2)^{1/2}}(|F_A|^2(\nu\tilde{n} - \tfrac{1}{2}) + |F_B|^2(\nu\tilde{n} + \tfrac{1}{2})).$$

Similarly,

$$(\nabla\boldsymbol{\psi}^\dagger)\boldsymbol{\psi} = \frac{-i}{\sqrt{a^2+b^2}(a^2+b^2)^{1/2}}(|F_A|^2(\nu\tilde{n} - \tfrac{1}{2}) + |F_B|^2(\nu\tilde{n} + \tfrac{1}{2})).$$

Noting[1] that $|F_A|^2 + |F_B|^2 = 1$, and

$$|F_A|^2(\nu\tilde{n} - \tfrac{1}{2}) + |F_B|^2(\nu\tilde{n} + \tfrac{1}{2}) = \nu\tilde{n}(|F_A|^2 + |F_B|^2) + \tfrac{1}{2}(|F_B|^2 - |F_A|^2) = \nu\tilde{n} - \tfrac{s}{2}\cos\theta$$

we obtain the formula

$$j = \frac{2e\hbar}{m_e(a^2+b^2)}(\nu\tilde{n} - \tfrac{s}{2}\cos\theta).$$

This is because

$$\psi^\dagger \frac{d}{d\ell}\psi \to \frac{\psi^\dagger}{(a^2+b^2)^{1/4}}\frac{d}{d\ell}\left(\frac{\psi}{(a^2+b^2)^{1/4}}\right).$$

Taking the derivative $d/d\ell$ brings down a factor $1/\sqrt{a^2+b^2}$. The overall length scaling factor is:

$$\frac{1}{(a^2+b^2)^{1/4}}\frac{1}{\sqrt{a^2+b^2}}\frac{1}{(a^2+b^2)^{1/4}} = \frac{1}{a^2+b^2}.$$

Plugging some numbers in the multiplicative coefficient:

$$j = \frac{(2)(1.6\times 10^{-19}\ \mathrm{C})(1.05\times 10^{-34}\ \mathrm{J.s})}{(9.1\times 10^{-31}\ \mathrm{kg})[(2\times 10^{-9}\ \mathrm{m})^2 + (3.38\times 10^{-9}\ \mathrm{m})^2]}\left(\frac{n}{2M\mathcal{N}} + \tfrac{s}{2}\cos\theta\right),$$

or

$$j = 2\left(\frac{n}{2M\mathcal{N}} + \tfrac{s}{2}\cos\theta\right)(1.2\times 10^{-6})\ \mathrm{A}.$$

We get the correct units (Ampère) after having rescaled the wavefunction. Let's check the magnitude. For $\theta = \frac{\pi}{2}$, $n = M = 10$ and $\mathcal{N} = 1$, the current j is 2.4 μA. Since $b = 3.38$ nm, the length of one helix is 33.8 nm. (Similarly, $\mathcal{N} = 3$, $n = M$ gives a current of 800 nA whereas $n = 1$, $M = 10$, $\mathcal{N} = 1$, gives 80 nA.) For comparison, the I-V curves presented in the paper by Wang [7] (see Fig. 4) show currents up to 220 nA.

1.4.1. *Embedding of 1D Spinor Wavefunction in 3D.* The normalized Varela spinors (see Section 1.4.4)

$$\boldsymbol{\psi}(\varphi) = \frac{1}{\sqrt{2\pi s}}\begin{bmatrix} F_A e^{-i\varphi/2} \\ \zeta F_B^* e^{i\varphi/2}\end{bmatrix} e^{i\nu\tilde{n}\varphi}\chi_{[0,2\pi]}(\varphi)$$

one-parameter mappings $\boldsymbol{\psi} : \varphi \in [0, 2\pi] \mapsto \mathbb{C}^2$. However, computation of the Fermi contact interaction requires three-dimensional wavefunctions because the Dirac delta distribution in the Hamiltonian, $\delta^{(3)}(\mathbf{r} - \mathbf{r}_0)$, must be integrated over three dimensional space. It is unclear how to embed a 1D function into a 3D space because there are an infinite number of possibilities.

We can limit the options by invoking a simple argument from probability theory. Let's say we have a random variable X and its associated probability density function, $p_X(x)$. By definition, it must be normalized:

$$\int_{-\infty}^{\infty} p_X(x)dx = 1.$$

[1]

$$|F_A|^2 = F_A^*F_A = \frac{|s|}{4}(se^{-i\theta/2} + e^{i\theta/2})(se^{i\theta/2} + e^{-i\theta/2}) = \frac{1}{4}(s^2 + s(e^{-i\theta} + e^{i\theta}) + 1) = \frac{1}{2}(1 + s\cos\theta)$$

$$|F_B|^2 = F_B^*F_B = \frac{|s|}{4}(se^{i\theta/2} - e^{-i\theta/2})(se^{-i\theta/2} - e^{i\theta/2}) = \frac{1}{4}(s^2 - s(e^{-i\theta} + e^{i\theta}) + 1) = \frac{1}{2}(1 - s\cos\theta)$$

$$|F_A|^2 + |F_B|^2 = 1, \qquad |F_A|^2 - |F_B|^2 = s\cos\theta$$

If we also want it to be a function of y and z in order to describe the behavior of two additional random variables, Y and Z. If X, Y and Z are statistically independent, the joint probability density is a product. It must be normalized as well:

$$\int_{-\infty}^{\infty}\int_{-\infty}^{\infty}\int_{-\infty}^{\infty} p_X(x)p_Y(y)p_Z(z)\,dx\,dy\,dz = 1.$$

We must then have:

$$\int_{-\infty}^{\infty} p_Y(y)dy = 1, \qquad \int_{-\infty}^{\infty} p_Z(z)dz = 1.$$

What are the requirements on p_Y and p_Z? In the context of quantum mechanics, the norm of the wavefunction must have dimension of inverse length since α and β have dimensions of length. Let's call these functions f and g:

$$\int_{-\infty}^{\infty} f(\alpha)d\alpha = 1, \qquad \int_{-\infty}^{\infty} g(\beta)d\beta = 1.$$

When averaging a distance, we would like to fix the values of the averages, i.e. $\langle\alpha\rangle = a$, $\langle\beta\rangle = a$,

$$\langle\alpha\rangle = \int_{-\infty}^{\infty} \alpha f(\alpha)d\alpha := a, \qquad \langle b\rangle = \int_{-\infty}^{\infty} \beta g(\beta)d\beta := b.$$

The choice of distribution is not so important[2] as long as the correct averages are obtained when computing the moments of interest. We choose the uniform distribution for simplicity:

$$f(\alpha) = \frac{2}{a} \text{ for } 0 \le \alpha \le a.$$

In the context of a quantum mechanical wavefunction, we would take a one-dimensional wavefunction $\psi(\varphi)$ and construct a three-dimensional function

$$\Psi(\alpha,\beta,\varphi) = \psi(\varphi)\sqrt{\frac{2}{a}}\chi_{[0,a]}(\alpha)\sqrt{\frac{2}{b}}\chi_{[0,b]}(\beta)$$

such that the probability gives us the correct average position:

$$\begin{aligned}\langle\alpha\rangle &= \int_{-\infty}^{\infty}\int_{-\infty}^{\infty}\int_{-\infty}^{\infty} |\Psi(\alpha,\beta,\varphi)|^2\alpha\,d\beta\,d\alpha\,d\varphi \\ &= \int_{-\infty}^{\infty}\int_{-\infty}^{\infty}\int_{-\infty}^{\infty} |\psi(\varphi)|^2\frac{2}{a}\chi_{[0,a]}(\alpha)\frac{2}{b}\chi_{[0,b]}(\beta)\alpha\,d\beta\,d\alpha\,d\varphi = a\end{aligned}$$

$$\begin{aligned}\langle\beta\rangle &= \int_{-\infty}^{\infty}\int_{-\infty}^{\infty}\int_{-\infty}^{\infty} |\Psi(\alpha,\beta,\varphi)|^2\beta\,d\beta\,d\alpha\,d\varphi \\ &= \int_{-\infty}^{\infty}\int_{-\infty}^{\infty}\int_{-\infty}^{\infty} |\psi(\varphi)|^2\frac{2}{a}\chi_{[0,a]}(\alpha)\frac{2}{b}\chi_{[0,b]}(\beta)\beta\,d\beta\,d\alpha\,d\varphi = b.\end{aligned}$$

[2]Choosing a different distribution will affect the overall scaling slightly, depending on the moments of the distribution. For example, if a random variable X follows a Rayleigh distribution with PDF $f(x) = \frac{x}{\sigma^2}e^{-x^2/(2\sigma^2)}$, $x \ge 0$ we would fulfill the requirements of a continuous wavefunction and first derivative across space. However, the mean is $\langle X\rangle = \sigma\sqrt{\pi/2}$. One may argue that the extra factor $\sqrt{\pi/2}$ does significantly alter the result. We could also impose additional conditions on the moments if needed.

We therefore redefine the Varela spinors as follows:

$$\boldsymbol{\psi}(\alpha,\beta,\varphi)=\frac{1}{\sqrt{2\pi s}}\begin{bmatrix}F_A e^{-i\varphi/2}\\ \zeta F_B^* e^{i\varphi/2}\end{bmatrix} e^{i\nu\tilde{n}\varphi}\chi_{[0,2\pi]}(\varphi)\sqrt{\frac{2}{a}}\chi_{[0,a]}(\alpha)\sqrt{\frac{2}{b}}\chi_{[0,b]}(\beta).$$

In this manner, integration over $\mathbb{R}^3$ can be performed. When computing averages of α and β, integration over all space of the norm of this wavefunction will return a and b, the correct radius and pitch of the helix.

1.4.2. *Matrix Elements of the 3D Dirac Delta Function.* Let us return to the Fermi contact interaction and start by checking that sensible results are obtained upon integration. In helicoidal coordinates the 3D Dirac distribution is (see Section 1.4.3):

$$\delta^{(3)}(\mathbf{r}-\mathbf{r}_0)=\frac{1}{|a\varphi|}\delta(a-a_0)\delta(b-b_0)\delta(\varphi-\varphi_0).$$

The three averages needed are ($a>0$, $b>0$, $\varphi>0$ so the absolute values are unimportant):

$$\begin{aligned}&\langle\boldsymbol{\psi}^{\nu,\zeta}_{n',s}|\sigma_x\delta^{(3)}(\mathbf{r}-\mathbf{R}_1)|\boldsymbol{\psi}^{\nu,\zeta}_{n,s}\rangle\\ &\quad=\lim_{\epsilon\to0}\int_{-\infty}^{\infty}\int_{-\infty}^{\infty}\int_{-\infty}^{\infty}\frac{1}{2\pi s}(F_A^*F_B^*e^{i\varphi/2}\zeta e^{i\varphi/2}+\zeta F_AF_Be^{-i\varphi/2}e^{-i\varphi/2})e^{i\nu(\tilde{n}-\tilde{n}')\varphi}\\ &\quad\times\frac{1}{|\alpha\varphi|}\delta(\alpha-a+\epsilon)\delta(\beta-b+\epsilon)\delta(\varphi-\varphi_1+\epsilon)\frac{2}{a}\chi_{[0,a]}(\alpha)\frac{2}{b}\chi_{[0,b]}(\beta)\chi_{[0,2\pi]}(\varphi)\,d\beta\,d\alpha\,d\varphi\\ &\quad=\frac{2}{\pi s}\frac{1}{a^2b}\zeta(F_AF_Be^{-i\varphi_1}+c.c.)\frac{e^{i\nu(\tilde{n}-\tilde{n}')\varphi_1}}{|\varphi_1|}\end{aligned}$$

where the limiting procedure $\lim_{\epsilon\to0}$ was introduced to ensure that the Dirac delta function is concentrated inside the domain of integration, as defined by the indicator functions. Next,

$$\begin{aligned}&\langle\boldsymbol{\psi}^{\nu,\zeta}_{n',s}|\sigma_y\delta^{(3)}(\mathbf{r}-\mathbf{R}_1)|\boldsymbol{\psi}^{\nu,\zeta}_{n,s}\rangle\\ &\quad=\lim_{\epsilon\to0}\int_{-\infty}^{\infty}\int_{-\infty}^{\infty}\int_{-\infty}^{\infty}\frac{i}{2\pi s}(F_AF_Be^{-i\varphi/2}\zeta e^{-i\varphi/2}-\zeta F_A^*F_B^*e^{i\varphi/2}e^{i\varphi/2})e^{i\nu(\tilde{n}-\tilde{n}')\varphi}\\ &\quad\times\frac{1}{|\alpha\varphi|}\delta(\alpha-a+\epsilon)\delta(\beta-b+\epsilon)\delta(\varphi-\varphi_1+\epsilon)\frac{2}{a}\chi_{[0,a]}(\alpha)\frac{2}{b}\chi_{[0,b]}(\beta)\chi_{[0,2\pi]}(\varphi)\,d\beta\,d\alpha\,d\varphi\\ &\quad=\frac{2i}{\pi s}\frac{1}{a^2b}\zeta(F_AF_Be^{-i\varphi_1}-c.c.)\frac{e^{i\nu(\tilde{n}-\tilde{n}')\varphi_1}}{|\varphi_1|}.\end{aligned}$$

Finally,

$$\begin{aligned}\langle\boldsymbol{\psi}^{\nu,\zeta}_{n',s}|\sigma_z\delta^{(3)}(\mathbf{r}-\mathbf{R}_1)|\boldsymbol{\psi}^{\nu,\zeta}_{n,s}\rangle&=\lim_{\epsilon\to0}\int_{-\infty}^{\infty}\int_{-\infty}^{\infty}\int_{-\infty}^{\infty}(|F_A|^2-|F_B|^2)e^{i\nu(\tilde{n}-\tilde{n}')\varphi}\\ &\times\frac{1}{|\alpha\varphi|}\delta(\alpha-a+\epsilon)\delta(\beta-b+\epsilon)\delta(\varphi-\varphi_1+\epsilon)\frac{2}{a}\chi_{[0,a]}(\alpha)\frac{2}{b}\chi_{[0,b]}(\beta)\chi_{[0,2\pi]}(\varphi)\,d\beta\,d\alpha\,d\varphi\\ &=\frac{2}{\pi s}\frac{1}{a^2b}(|F_A|^2-|F_B|^2)\frac{e^{i\nu(\tilde{n}-\tilde{n}')\varphi_1}}{|\varphi_1|}.\end{aligned}$$

1.4.3. *Pull-back of the Delta Function in Curvilinear Coordinates.* Consider the mapping:

$$(a, b, \varphi) \mapsto (x, y, z) = (a\cos\varphi, a\sin\varphi, b\varphi).$$

The Jacobian of this transformation is:

$$\frac{\partial(x,y,z)}{\partial(a,b,\varphi)} = \frac{\partial(a\cos\varphi, a\sin\varphi, b\varphi)}{\partial(a,b,\varphi)} = \begin{bmatrix} \cos\varphi & 0 & -a\sin\varphi \\ \sin\varphi & 0 & a\cos\varphi \\ 0 & \varphi & b \end{bmatrix}.$$

The determinant of which is $-a\varphi\sin^2\varphi - a\varphi\cos^2\varphi = -a\varphi$. Therefore, the 3D Dirac delta function is

$$\delta^{(3)}(\mathbf{r}-\mathbf{r}_0) = \frac{1}{|a\varphi|}\delta(a-a_0)\delta(b-b_0)\delta(\varphi-\varphi_0).$$

Let $\Phi : A \mapsto B$. This expression has the following meaning:

$$\int_B f(\mathbf{r}) \boxed{\delta^{(3)}(\mathbf{r}-\mathbf{r}_0)} d^3\mathbf{r} = f(\mathbf{r}_0) = \int_A (f\circ\Phi)(a,b,\varphi) \boxed{\frac{1}{|a\varphi|}\delta(a-a_0)\delta(b-b_0)\delta(\varphi-\varphi_0)} |a\varphi|\, da\, db\, d\varphi$$

$$= \int_A (f\circ\Phi)(a,b,\varphi)\delta(a-a_0)\delta(b-b_0)\delta(\varphi-\varphi_0)\, da\, db\, d\varphi = (f\circ\Phi)(a_0,b_0,\varphi_0),$$

where $(f\circ\Phi)(a,b,\varphi)$ denotes the pull-back of the scalar-valued function f, i.e. $(\Phi^* f)(a,b,\varphi)$.

1.4.4. *Normalization of Spinors.* In the 2016 paper the Varela spinors are defined as:

$$\boldsymbol{\psi}^{\nu,\zeta}_{n,s}(\varphi) = \begin{bmatrix} F_A e^{-i\varphi/2} \\ \zeta F_B^* e^{i\varphi/2} \end{bmatrix} e^{i\nu\tilde{n}\varphi}.$$

For convenience, we abbreviate $\boldsymbol{\psi}^{\nu,\zeta}_{n,s}(\varphi)$ as $\boldsymbol{\psi}(\varphi)$ or simply, $\boldsymbol{\psi}$. The inner product is:

$$\boldsymbol{\psi}^\dagger\boldsymbol{\psi} = \begin{bmatrix} F_A^* e^{i\varphi/2} & \zeta F_B e^{-i\varphi/2} \end{bmatrix} e^{-i\nu\tilde{n}\varphi} \begin{bmatrix} F_A e^{-i\varphi/2} \\ \zeta F_B^* e^{i\varphi/2} \end{bmatrix} e^{i\nu\tilde{n}\varphi} = |F_A|^2 + \zeta^2|F_B|^2 = |F_A|^2 + |F_B|^2 = s$$

since $|F_A|^2 + |F_B|^2 = s$. When normalizing, we integrate over all space:

$$\langle\boldsymbol{\psi}^\dagger|\boldsymbol{\psi}\rangle = \int_0^{2\pi} \boldsymbol{\psi}^\dagger(\varphi)\boldsymbol{\psi}(\varphi) d\varphi = 2\pi s.$$

Therefore, proper normalization of the probability amplitudes requires a factor of $\frac{1}{\sqrt{2\pi s}}$. Also, for convenience we will multiply them with an indicator function:

$$\chi_{[0,2\pi]}(\varphi) = \begin{cases} 1 & \varphi \in [0, 2\pi] \\ 0 & \text{otherwise} \end{cases}.$$

This enables us to extend the integration limits to infinity. We define them as:

$$\boldsymbol{\psi}^{\nu,\zeta}_{n,s}(\varphi) = \frac{1}{\sqrt{2\pi s}} \begin{bmatrix} F_A e^{-i\varphi/2} \\ \zeta F_B^* e^{i\varphi/2} \end{bmatrix} e^{i\nu\tilde{n}\varphi}\chi_{[0,2\pi]}(\varphi).$$

Then, the factor of 2π is not needed. We can also integrate over all space:

$$\langle\boldsymbol{\psi}^\dagger|\boldsymbol{\psi}\rangle = \int_{-\infty}^{\infty} \boldsymbol{\psi}^\dagger(\varphi)\boldsymbol{\psi}(\varphi)\chi_{[0,2\pi]}(\varphi) d\varphi = 1.$$

2. Text S2. Enantiospecificity in Cross-Polarization

The dependence of the bilinear coupling $\mathbf{I}_1 \cdot \mathsf{F} \cdot \mathbf{I}_2$ on chirality implies that spin-relaxation may also depend on chirality. Because the kinetics of the cross-polarization experiment depend on spin relaxation times, we look at the impact of the indirect coupling on spin-lattice relaxation in the rotating frame. Consider a general Hamiltonian of the form $\mathcal{H} = \mathcal{H}_Z + \mathcal{H}_{SB} + \mathcal{H}_B$, where $\mathcal{H}_Z$ is the unperturbed spin part (e.g. Zeeman interaction), $\mathcal{H}_{SB}$ is the spin-bath interaction including any spin-spin interactions and $\mathcal{H}_B$ is the bath Hamiltonian. We denote the corresponding Liouvillian superoperator as $\mathsf{L} = \mathsf{L}_Z + \mathsf{L}_{SB} + \mathsf{L}_B$. The Liouvillian superoperator is defined as the commutation superoperator, $\mathsf{L}\rho \equiv i[\mathcal{H}, \rho]$. Consider a set of operators $\{F_k\}_{k=1}^m$ and an inner product $(A, B) = \mathrm{Tr}(A^\dagger B)$, often denoted $\langle\langle A|B\rangle\rangle$. The operators F_k are orthogonal, $\langle\langle F_i|F_j\rangle\rangle = 0$ for $i \neq j$. A projection superoperator can be defined as $\pi = \sum_{k=1}^m \frac{|F_k\rangle\rangle\langle\langle F_k|}{\langle\langle F_k|F_k\rangle\rangle}$. It is customary to transform the Liouvillian to the interaction representation (see Section 2.4) generated by L_Z and write the resulting Liouvillian as $\mathsf{L}^*(t) \equiv \mathsf{L}_B + \mathsf{L}_{SB}(t)$, where $*$ denotes the interaction representation and $\mathsf{L}_{SB}(t) \equiv e^{\mathsf{L}_z t}\mathsf{L}_{SB}$. It will be convenient to study irreversible processes using projection operator methods, which yield a quantum master equation for an observable F_k:

$$\begin{aligned}\frac{d}{dt}\langle\langle F_k|\rho(t)\rangle\rangle = &- i\langle\langle F_k|\mathsf{L}^*(t)\pi|\rho(t)\rangle\rangle - i\langle\langle F_k|\mathsf{L}^*(t)\vec{T}e^{-i\int_0^t d\tau(1-\pi)\mathsf{L}^*(\tau)}(1-\pi)|\rho(0)\rangle\rangle \\ &- \sum_{j=1}^m \int_0^t dt' \frac{\langle\langle F_k|\mathsf{L}^*(t)\vec{T}e^{-i\int_{t'}^t d\tau(1-\pi)\mathsf{L}^*(\tau)}(1-\pi)\mathsf{L}^*(t')|F_j\rangle\rangle}{\langle\langle F_j|F_j\rangle\rangle}\langle\langle F_j|\rho(t')\rangle\rangle\end{aligned}$$

where $\vec{T}$ denotes Dyson time-ordering of the exponential [8]. Insertion of the expression for the projection operator π into the first term shows that it causes coherent evolution (Bloch term). The second term can be made to vanish with a proper choice of initial conditions. The last term is the dissipative term we are interested in. Under some assumptions (weak collision limit, short correlation time limit, average Liouvillian, stationarity of the memory kernel), $\vec{T}e^{-i\int_{t'}^t d\tau(1-\pi)\mathsf{L}^*(\tau)}$ is approximated by $e^{-i(t-t')(1-\pi)\mathsf{L}^*}$, where L^* is a time-averaged Liouvillian in the interaction representation. It is customary to drop the Zeeman and spin-bath parts ($\|\mathsf{L}_B\| \gg \|\mathsf{L}_Z + \mathsf{L}_{SB}\|$), leaving only the bath part, i.e. $\mathsf{L}^* \approx \mathsf{L}_B$, in the exponential. Since there is no spin part left, the projector term is dropped leaving $e^{-i(t-t')\mathsf{L}_B}$. Finally, the term $(1-\pi)\mathsf{L}^*(t')|F_j\rangle\rangle$ gives two terms: $\mathsf{L}^*(t')|F_j\rangle\rangle$ and $-\pi\mathsf{L}^*(t')|F_j\rangle\rangle$. The second term is not of interest in the derivation of dissipation rates because it describes a product of average frequencies; we are instead interested in deviations from averages. We therefore consider the first term. The dissipation term in this wide-sense stationary Redfield limit reads:

$$\sum_{j=1}^m \int_0^\infty d\tau \frac{\langle\langle F_k|\mathsf{L}^*(0)e^{i\tau\mathsf{L}_B}\mathsf{L}^*(\tau)|F_j\rangle\rangle}{\langle\langle F_j|F_j\rangle\rangle}\langle\langle F_j|\rho(t)\rangle\rangle \equiv \sum_{j=1}^m \langle\langle F_j|\rho(t)\rangle\rangle \int_0^\infty \mathcal{K}_{kj}(\tau)d\tau, \tag{7}$$

where $W(F_k) \equiv \int_0^\infty \mathcal{K}_{kj}(\tau)d\tau$ is a dissipation rate for the state F_k. In the study of cross-polarization we shall be interested in the quantity $T_{1\rho}$, the spin-lattice relaxation time in the rotating frame. For $\{F_k\}$ we will take the set $\{F_x, F_y, F_z\}$, where $F_j \equiv I_j^u$, $u = 1, 2$.

2.1. **Homonuclear Case.** We begin with the homonuclear case. While homonuclear spin systems are not commonplace in cross-polarization, such experiments are possible, involving different interacting subensembles. Two interacting nuclear spins of the same type (homonuclear case), $\mathbf{I}_1$ and $\mathbf{I}_2$ are coupled to a bath of electron spins $\{\mathbf{S}_l\}$. The spin-bath interaction

is taken to be a sum of pairwise couplings. In a spin-lock experiment where $T_{1\rho}$ is normally measured, the Zeeman Hamiltonian $\mathcal{H}_Z$ is the sum of two components, the Zeeman and RF parts (in frequency units) are: $\mathcal{H}_Z = \omega_0(I_z^1 + I_z^2) + \omega_S \sum_l S_z^l + \omega_1(I_x^1 + I_x^2)\cos(\omega_0 t)$. For simplicity: 1) the two nuclear spins are assumed to be chemically and magnetically equivalent; 2) we also neglect off-resonance effects for simplicity. (Those assumptions can easily be dropped, posing no difficulties, but here we'd like to keep our expressions simple.) The Hermitian operator $\omega_0(I_z^1+I_z^2)+\omega_S\sum_l S_z^l$ can be used to effect a transformation to the "doubly rotating frame". In the secular (rotating-wave) approximation this leaves $\mathcal{H}_Z^* = \omega_1(I_x^1+I_x^2)$. The nuclear-nuclear term of the spin-bath Hamiltonian in the rotating frame, $\mathcal{H}_{SB}^*$, acquires a phase factor $e^{-i\omega_0 qt}$:

$$\mathcal{H}_{SB}^*(t) = \sum_{q=-2}^{2}(-1)^q \underbrace{A_{2,q}(\mathbf{I}^1,\mathbf{I}^2)T_{2,-q}(\mathbf{I}^1,\mathbf{I}^2)e^{-i\omega_0 qt}}_{\text{nuclear-nuclear}}$$
$$+\sum_{j=1}^{m}\underbrace{\left[A_{2,0}(\mathbf{I}^1,\mathbf{S}^j)T_{2,0}^*(\mathbf{I}^1,\mathbf{S}^j) + A_{2,0}(\mathbf{I}^2,\mathbf{S}^j)T_{2,0}^*(\mathbf{I}^2,\mathbf{S}^j)\right]}_{\text{electron-nuclear}}$$

whereas the electron-nuclear term does not. It is also truncated to longitudinal order. Instead of describing an "effective" interaction, the components $A_{2,0}(\mathbf{I}^2,\mathbf{S}^j)$ of the electron-nuclear tensor would typically be the components of a direct hyperfine interaction. On the other hand, the $k=2$ components of the spherical tensor A coupling nuclear spins $\mathbf{I}^1$ to $\mathbf{I}^2$ given in terms of the Cartesian components of the indirect coupling tensor F are:

$$\begin{aligned}
A_{2,0}(\mathbf{I}^1,\mathbf{I}^2) &= \frac{-\mathsf{F}_{xx}-\mathsf{F}_{yy}+2\mathsf{F}_{zz}}{\sqrt{6}}\\
A_{2,\pm1}(\mathbf{I}^1,\mathbf{I}^2) &= \mp\,\frac{\mathsf{F}_{zx}+\mathsf{F}_{xz}\pm i(\mathsf{F}_{zy}+\mathsf{F}_{yz})}{2}\\
A_{2,\pm2}(\mathbf{I}^1,\mathbf{I}^2) &= \frac{\mathsf{F}_{xx}-\mathsf{F}_{yy}\pm i(\mathsf{F}_{xy}+\mathsf{F}_{yx})}{2}.
\end{aligned}$$

For the case of the dipole interaction, the tensor components can be inferred from Eq. (5). Explicitly, they are:

$$\text{(8)}\qquad \mathsf{F}_{\alpha\alpha'} = \left(\frac{\mu_0}{4\pi}\right)^2\gamma_I^2\gamma_S^2\sum_{n,n'}\sum_{\beta,\beta'}\frac{M_{1,\beta}^{\alpha\beta}(\tilde{n}',\tilde{n})M_{2,\beta'}^{\alpha'\beta'}(\tilde{n},\tilde{n}')}{|T|(\tilde{n}'-\tilde{n})}f(\tilde{n})[1-f(\tilde{n}')] + c.c.$$

where the M matrices are given by Eqs. (2), (3) and (4). As discussed in Section 1.2.1 the tensor $\sum_{\beta,\beta'} M_{1,\beta}^{\alpha\beta}(\tilde{n}',\tilde{n})M_{2,\beta'}^{\alpha'\beta'}(\tilde{n},\tilde{n}')$ is enantiospecific. Therefore, the tensor components $\mathsf{F}_{\alpha\alpha'}$ will be enantiospecific. And as we will see later, the relaxation rate $(T_{1\rho})^{-1}$ will be enantiospecific. The irreducible tensors for the homonuclear interaction are:

$$T_{2,0}(\mathbf{I}^1,\mathbf{I}^2) = \frac{1}{\sqrt{6}}(3I_z^1I_z^2 - \mathbf{I}^1\cdot\mathbf{I}^2)\quad T_{2,\pm1}(\mathbf{I}^1,\mathbf{I}^2) = \mp\frac{1}{2}(I_z^1I_\pm^2 + I_\pm^1I_z^2)\quad T_{2,\pm2}(\mathbf{I}^1,\mathbf{I}^2) = \frac{1}{2}I_\pm^1I_\pm^2$$

whereas tensor components for the electron-nuclear interactions with $q=\pm1,\pm2$ average to zero except for $q=0$, which becomes

$$T_{2,0}^*(\mathbf{I}^1,\mathbf{S}^j) = \lim_{T\to\infty}\frac{1}{T}\int_0^T e^{-i\omega_0(I_z^1+I_z^2)t}T_{2,0}(\mathbf{I}^1,\mathbf{S}^j)e^{i\omega_0(I_z^1+I_z^2)t}dt = \sqrt{2/3}\,I_zS_z.$$

Next, to eliminate the remaining Zeeman term, $\mathcal{H}_Z^* = \omega_1(I_x^1 + I_x^2)$ from the Hamiltonian we effect another transformation to a second interaction frame using this residual Zeeman term (see Section 2.4). This involves rotating the spin-bath Hamiltonian $\mathcal{H}_{SB}^*(\tau)$ by an angle $\omega_1 t$ about the X axis. This can be accomplished with the help of Wigner matrices. The tensors $T_{2,-q}(\mathbf{I}^1, \mathbf{I}^2)$ transform as:

$$T_{2,-q}(\mathbf{I}^1, \mathbf{I}^2) \rightarrow \mathbf{R}(\Omega) T_{k,-q} = \sum_{q'=-2}^{2} T_{2,-q'}(\mathbf{I}^1, \mathbf{I}^2) \mathscr{D}_{q',-q}^{(2)}(\Omega)$$

where $\Omega = \alpha\,\beta\,\gamma$ is a set of Euler angles, $\mathbf{R}(\Omega)$ is a rotation operator and $\mathscr{D}_{m'm}^{(j)}(\alpha\,\beta\,\gamma) = e^{-i(m'\alpha+m\gamma)} d_{m'm}^{(j)}(\beta)$. From the definition of Euler rotation, $\mathbf{R}(\alpha\,\beta\,\gamma) = \mathbf{R}_z(\alpha)\mathbf{R}_y(\beta)\mathbf{R}_z(\gamma)$, a rotation about the X axis by an angle β can be effected using the sequence $\mathbf{R}(\frac{\pi}{2}, \beta, -\frac{\pi}{2}) = \mathbf{R}_z(\frac{\pi}{2})\mathbf{R}_y(\beta)\mathbf{R}_z(-\frac{\pi}{2})$. The elements of the Wigner matrix are therefore

$$\mathscr{D}_{m'm}^{(j)}(\tfrac{\pi}{2}, \beta, -\tfrac{\pi}{2}) = e^{-i(m'\pi/2 - m\pi/2)} d_{m'm}^{(j)}(\beta),$$

where $\beta = \omega_1 t$. Thus, $\mathscr{D}_{q',-q}^{(2)}(\frac{\pi}{2}, \omega_1 t, -\frac{\pi}{2}) = e^{-i(q'\pi/2 + q\pi/2)} d_{q',-q}^{(2)}(\omega_1 t)$.

Finally, the electron-nuclear tensor component $T_{2,0}^*(\mathbf{I}^1, \mathbf{S}^j)$ transforms according to the rule:

$$T_{2,0}^*(\mathbf{I}^1, \mathbf{S}^j) \rightarrow \sqrt{2/3} S_z^j \left(I_z^1 \cos(\omega_1 t) - I_y^1 \sin(\omega_1 t) \right).$$

The spin-bath Hamiltonian in this second interaction representation is therefore:

$$\begin{aligned}\mathcal{H}_{SB}^{**}(t) = &\sum_{q'=-2}^{2} \sum_{q=-2}^{2} (-1)^q A_{2,q}(\mathbf{I}^1, \mathbf{I}^2) T_{2,-q}(\mathbf{I}^1, \mathbf{I}^2) e^{-i\omega_0 q t} e^{-i(q'\pi/2 + q\pi/2)} d_{q',-q}^{(2)}(\omega_1 t) \\ &+ \sqrt{2/3} \sum_{j=1}^{m} \Big[A_{2,0}(\mathbf{I}^1, \mathbf{S}^j) S_z^j \left(I_z^1 \cos(\omega_1 t) - I_y^1 \sin(\omega_1 t) \right) \\ &\qquad + A_{2,0}(\mathbf{I}^2, \mathbf{S}^j) S_z^j \left(I_z^2 \cos(\omega_1 t) - I_y^2 \sin(\omega_1 t) \right) \Big].\end{aligned}$$

We could of course carry on with the calculation including all terms. While this poses no extra difficulties other than additional bookkeeping, it carries no pedagogical value. Since, our goal is to provide an intuitive understanding of the link between chirality and nuclear spin relaxation, we simplify the expression by dropping all terms where $q \neq 0$. This is equivalent to assuming that motion is slower than the Larmor period $(2\pi/\omega_1)$. Using the middle column $(q = 0)$ of the reduced Wigner matrix

(9)

$$d^{(2)}(\beta) = \begin{pmatrix} \cos^4(\beta/2) & -\frac{1}{2}\sin\beta(1+\cos\beta) & \sqrt{\frac{3}{8}}\sin^2\beta & \frac{1}{2}\sin\beta(\cos\beta-1) & \sin^4(\beta/2) \\ \frac{1}{2}\sin\beta(1+\cos\beta) & \frac{1}{2}(2\cos\beta-1)(\cos\beta+1) & -\sqrt{\frac{3}{2}}\sin\beta\cos\beta & \frac{1}{2}(2\cos\beta+1)(1-\cos\beta) & \frac{1}{2}\sin\beta(\cos\beta-1) \\ \sqrt{\frac{3}{8}}\sin^2\beta & \sqrt{\frac{3}{2}}\sin\beta\cos\beta & \frac{1}{2}(3\cos^2\beta-1) & -\sqrt{\frac{3}{2}}\sin\beta\cos\beta & \sqrt{\frac{3}{8}}\sin^2\beta \\ -\frac{1}{2}\sin\beta(\cos\beta-1) & \frac{1}{2}(2\cos\beta+1)(1-\cos\beta) & \sqrt{\frac{3}{2}}\sin\beta\cos\beta & \frac{1}{2}(2\cos\beta-1)(\cos\beta+1) & -\frac{1}{2}\sin\beta(1+\cos\beta) \\ \sin^4(\beta/2) & -\frac{1}{2}\sin\beta(\cos\beta-1) & \sqrt{\frac{3}{8}}\sin^2\beta & \frac{1}{2}\sin\beta(1+\cos\beta) & \cos^4(\beta/2) \end{pmatrix}$$

the sum over q' is:

$$\sum_{q'=-2}^{2} e^{-iq'\pi/2} d^{(2)}_{q',0}(\omega_1 t) = -\sqrt{\frac{3}{8}}\sin^2(\omega_1 t) + i\sqrt{\frac{3}{2}}\sin(\omega_1 t)\cos(\omega_1 t) + \frac{1}{2}(3\cos^2(\omega_1 t) - 1)$$
$$+ (-i)\sqrt{\frac{3}{2}}\sin\beta\cos(\omega_1 t) - \sqrt{\frac{3}{8}}\sin^2(\omega_1 t)$$
$$= \frac{1}{8}\left(3e^{-2i\omega_1 t} + \left(2\sqrt{6}+3\right)e^{2i\omega_1 t} - 2\sqrt{6} + 2\right)$$

The spin-bath Hamiltonian becomes:

$$\mathcal{H}^{**}_{SB}(t) = A_{2,0}(\mathbf{I}^1, \mathbf{I}^2) T_{2,0}(\mathbf{I}^1, \mathbf{I}^2) \frac{1}{8}\left(3e^{-2i\omega_1 t} + \left(2\sqrt{6}+3\right)e^{2i\omega_1 t} - 2\sqrt{6} + 2\right)$$
$$+ \sqrt{2/3}\sum_{u=1,2}\sum_{j=1}^{m} A_{2,0}(\mathbf{I}^u, \mathbf{S}^j) S^j_z \left(I^u_z \cos(\omega_1 t) - I^u_y \sin(\omega_1 t)\right)$$

We are interested in how nuclear spin 1 (or 2) relaxes under conditions of spin-lock. Denote by u the spin of interest ($u = 1, 2$). Write down the operator corresponding to its state in terms of the basis

$$|F_u\rangle\rangle = \mathscr{Y}^{(1)q}(\mathbf{I}^u)$$

where $q = -1, 0, 1$. We now compute the numerator of the memory function,

$$\langle\langle F_k | \mathbf{L}^{**}(0) e^{i\tau \mathbf{L}_B} \mathbf{L}^{**}(\tau) | F_j \rangle\rangle.$$

According to Eq. (7), when a state is prepared along X, spin-locking is concerned with the evolution of the operator F_x. Therefore, three terms will contribute to the rate of thermalization of F_x:

$$\frac{\langle\langle F_x | \mathbf{L}^*(0) e^{i\tau \mathbf{L}_B} \mathbf{L}^*(\tau) | F_x \rangle\rangle}{\langle\langle F_x | F_x \rangle\rangle}, \quad \frac{\langle\langle F_x | \mathbf{L}^*(0) e^{i\tau \mathbf{L}_B} \mathbf{L}^*(\tau) | F_y \rangle\rangle}{\langle\langle F_y | F_y \rangle\rangle}, \quad \frac{\langle\langle F_x | \mathbf{L}^*(0) e^{i\tau \mathbf{L}_B} \mathbf{L}^*(\tau) | F_z \rangle\rangle}{\langle\langle F_z | F_z \rangle\rangle}$$

In order to illustrate the rate dependence on chirality, it will be sufficient to compute the first term only, with $F_x = I^u_x$, $u = 1, 2$. Starting from the commutator $\mathbf{L}^{**}(\tau) F_u \equiv [\mathcal{H}^{**}_{SB}(\tau), I^u_x]$. The nuclear-nuclear term has a commutator $[T_{2,0}(\mathbf{I}^1, \mathbf{I}^2), I^u_x)]$. Using the relation $[I_x, I_y] = iI_z$ (and cyclic permutations), we find

$$[T_{2,0}(\mathbf{I}^1, \mathbf{I}^2), I^1_x] = \frac{1}{\sqrt{6}}[3I^1_z I^2_z - \mathbf{I}^1 \cdot \mathbf{I}^2, I^1_x] = \frac{i}{\sqrt{6}}(2I^1_y I^2_z + I^1_z I^2_y)$$

and a similar expression exists for the commutator with I^2_x (by flipping 1 and 2). The electron-nuclear term has the commutator

$$[S^j_z(I^1_z \cos(\omega_1 t) - I^1_y \sin(\omega_1 t), I^1_x] = iS^j_z(I^1_y \cos(\omega_1 t) + I^1_z \sin(\omega_1 t)),$$

Acting on $\mathbf{L}^*(\tau)F$ with the bath propagator $e^{\mathbf{L}_B\tau}$ introduces a time dependence $A_{kq}(\mathbf{I}^1,\mathbf{I}^2)\to A_{kq}(\mathbf{I}^1,\mathbf{I}^2)(\tau)$. The final expression is:

$$e^{\mathbf{L}_B\tau}\mathbf{L}_{SB}^{**}(\tau)F_u = A_{2,0}(\mathbf{I}^1,\mathbf{I}^2)(\tau)\frac{i}{8\sqrt{6}}\left[\delta_{u,1}(2I_y^1I_z^2+I_z^1I_y^2)+\delta_{u,2}(2I_y^2I_z^1+I_z^2I_y^1)\right]$$
$$\times\left(3e^{-2i\omega_1\tau}+\left(2\sqrt{6}+3\right)e^{2i\omega_1\tau}-2\sqrt{6}+2\right)$$
$$+i\sqrt{2/3}(I_y^u\cos(\omega_1\tau)+I_z^u\sin(\omega_1\tau))\sum_{j=1}^{m}A_{2,0}(\mathbf{I}^u,\mathbf{S}^j)(\tau)S_z^j.$$

Next, we compute the inner product:

$$\frac{\langle\langle\mathbf{L}_{SB}^{**}(0)F_u|e^{\mathbf{L}_B\tau}\mathbf{L}_{SB}^{**}(\tau)F_u\rangle\rangle}{\langle\langle F_u|F_u\rangle\rangle}\equiv\frac{\mathrm{Tr}\left[(\mathbf{L}_{SB}^{**}(0)F_u)^\dagger e^{\mathbf{L}_B\tau}\mathbf{L}_{SB}^{**}(\tau)F_u\right]}{\mathrm{Tr}[F_u^\dagger F_u]},$$

for $F_u=I_x^u$. Upon multiplying the "bra" $\langle\langle\mathbf{L}_{SB}^{**}(0)I_x^u|$ and the "ket" $|e^{i\mathbf{L}_B\tau}\mathbf{L}_{SB}^{**}(\tau)I_x^u\rangle\rangle$, which involves taking a trace over all space, we get:

$$=C\cdot\langle A_{2,0}^\dagger(\mathbf{I}^1,\mathbf{I}^2)(0)A_{2,0}(\mathbf{I}^1,\mathbf{I}^2)(\tau)\rangle\left(3e^{-2i\omega_1\tau}+\left(2\sqrt{6}+3\right)e^{2i\omega_1\tau}-2\sqrt{6}+2\right)$$
$$+\frac{2}{3}C'\cdot\cos(\omega_1\tau)\sum_{j=1}^{m}\langle A_{2,0}^\dagger(\mathbf{I}^u,\mathbf{S}^j)(0)A_{2,0}(\mathbf{I}^u,\mathbf{S}^j)(\tau)\rangle_B.$$

where $C=\frac{1}{192}\frac{I^1(I^1+1)(2I^1+1)I^2(I^2+1)(2I^2+1)}{I^u(I^u+1)(2I^u+1)(2I^{\tilde{u}}+1)}=\frac{1}{192}I^{\tilde{u}}(I^{\tilde{u}}+1)$ and $C'=\frac{\mathrm{Tr}[(I_y^u)^2](S_z^j)^2]}{\mathrm{Tr}[(I_x^u)^2]}=\frac{1}{3}S^j(S^j+1)(2S^j+1)=\frac{1}{2}$ since $S^j=S=\frac{1}{2}$. We have used Tr $\left[(I_y^1I_z^2+I_z^1I_y^2)^2\right]=2\cdot\frac{1}{3}I^1(I^1+1)(2I^1+1)\cdot\frac{1}{3}I^2(I^2+1)(2I^2+1)$ and $\mathrm{Tr}_{I_1,I_2}[(I_x^1)^\dagger I_x^1]=\mathrm{Tr}_{I_1}[I_x^\dagger I_x]\cdot\mathrm{Tr}_{I_2}[\mathbf{1}]=\frac{1}{3}I^1(I^1+1)(2I^1+1)\cdot(2I^2+1)$. According to Abragam [9] the bath autocorrelation functions, $\langle A_{2,0}^\dagger(\mathbf{I}^1,\mathbf{I}^2)(0)A_{2,0}(\mathbf{I}^1,\mathbf{I}^2)(\tau)\rangle$ are computed as thermal averages, i.e.

$$\langle AA(t)\rangle_B\equiv\mathrm{tr}[\rho AA(t)],\quad\rho(\mathcal{H}_B)=\frac{\exp(-\beta_L\mathcal{H}_B)}{\mathrm{Tr}[\exp(-\beta_L\mathcal{H}_B)]},$$

which is the Boltzmann density matrix and tr denotes the partial trace over the bath degrees of freedom. β_L is the (inverse) temperature of the bath (lattice).

The spin-lock relaxation rate, $\frac{1}{T_{1\rho}}$, is therefore equal to:

$$\frac{1}{T_{1\rho}}=\sum_j\int_0^\infty\frac{\langle\langle F_k|\mathbf{L}^*(0)e^{i\tau\mathbf{L}_B}\mathbf{L}^*(\tau)|F_j\rangle\rangle}{\langle\langle F_j|F_j\rangle\rangle}d\tau$$
$$=\underbrace{3C\left[3J_{II}(-2\omega_1)+(2\sqrt{6}+3)J_{II}(2\omega_1)+2(1-\sqrt{6})J_{II}(0)\right]+\frac{3}{2}C'J_{IS}^c(\omega_1)}_{XX}+XY+XZ.$$

where XY corresponds to the $\langle\langle F_x^u|\cdot|F_y^u\rangle\rangle$ term and XZ, to the $\langle\langle F_x^u|\cdot|F_z^u\rangle\rangle$ term. The spectral density functions are:

$$J_{II}(\omega)=\int_0^\infty\langle A_{2,0}^\dagger(\mathbf{I}^1,\mathbf{I}^2)(0)A_{2,0}(\mathbf{I}^1,\mathbf{I}^2)(\tau)\rangle_B\,e^{i\omega\tau}\,d\tau,$$

$$J_{IS}^c(\omega)=\int_0^\infty\langle A_{2,0}^\dagger(\mathbf{I}^u,\mathbf{S}^j)(0)A_{2,0}(\mathbf{I}^u,\mathbf{S}^j)(\tau)\rangle_B\,\cos(\omega\tau)\,d\tau,$$

which was assumed, for simplicity, to be independent of u and j. The homonuclear spectral density function $J_{II}(\omega)$, and by extension, $T_{1\rho}$, is enantiospecific, since the $A_{2,0}(\mathbf{I}^1, \mathbf{I}^2)(\tau)$'s depend on the tensor components $\sum_{\beta,\beta'} M_{1,\beta}^{\alpha\beta}(\tilde{n}', \tilde{n}) M_{2,\beta'}^{\alpha'\beta'}(\tilde{n}, \tilde{n}')$, which are themselves enantiospecific.

2.2. **Heteronuclear Case.** The heteronuclear case is widely used in NMR to transfer polarization from high-gamma abundant nuclei to low-gamma dilute spins. The spin operators $\mathbf{I}_1$ and $\mathbf{I}_2$ now refer to different nuclei (e.g. ^{1}H and ^{13}C). The Zeeman Hamiltonian is now $\mathcal{H}_Z = \omega_{0,1} I_z^1 + \omega_{0,2} I_z^2 + \omega_S \sum_l S_z^l + \omega_{1,1} I_x^1 \cos(\omega_{0,1} t) + \omega_{1,2} I_x^2 \cos(\omega_{0,2} t)$. The Hartmann-Hahn condition corresponds to $\omega_{1,1} = \omega_{1,2}$, where $\omega_{1,1} = \gamma_{I_1} B_{1,1}$ and $\omega_{1,2} = \gamma_{I_2} B_{1,2}$. The operator $\omega_{0,1} I_z^1 + \omega_{0,2} I_z^2 + \omega_S \sum_l S_z^l$ is used to effect a transformation to the "interaction representation". In the secular (rotating-wave) approximation this leaves $\mathcal{H}_Z^* = \omega_{1,1} I_x^1 + \omega_{1,2} I_x^2$. We will limit our discussion to the heteronuclear $I - I$ term. The spin-bath Hamiltonian in the rotating frame, $\mathcal{H}_{SB}^*$ is:

$$\mathcal{H}_{SB}^*(t) = A_{2,0}(\mathbf{I}^1, \mathbf{I}^2) T_{2,0}^*(\mathbf{I}^1, \mathbf{I}^2)$$

where $T_{2,0}^*(\mathbf{I}^1, \mathbf{I}^2) = \sqrt{2/3} I_z^1 I_z^2$. Terms oscillating at $\omega_{0,1} - \omega_{0,2}$ or higher frequency have been dropped.

To eliminate the remaining Zeeman term, $\mathcal{H}_Z^* = \omega_{1,1} I_x^1 + \omega_{1,2} I_x^2$ from the Hamiltonian we effect another transformation to a second interaction frame using this residual Zeeman term (see Section 2.4). Using the rule

$$T_{2,0}^*(\mathbf{I}^1, \mathbf{I}^2) \to \sqrt{2/3} \left(I_z^1 \cos(\omega_{1,1}\tau) - I_y^1 \sin(\omega_{1,1}\tau)\right) \left(I_z^2 \cos(\omega_{1,2}\tau) - I_y^2 \sin(\omega_{1,2}\tau)\right),$$

the spin-bath Hamiltonian in this double interaction representation is therefore:

$$\mathcal{H}_{SB}^{**}(\tau) = \sqrt{2/3} A_{2,0}(\mathbf{I}^1, \mathbf{I}^2) \left(I_z^1 \cos(\omega_{1,1}\tau) - I_y^1 \sin(\omega_{1,1}\tau)\right) \left(I_z^2 \cos(\omega_{1,2}\tau) - I_y^2 \sin(\omega_{1,2}\tau)\right).$$

Commuting this with I_x^u, $u = 1, 2$:

$$\begin{aligned}\mathbf{L}_{SB}^{**}(\tau) I_x^u &= \sqrt{2/3} A_{2,0}(\mathbf{I}^1, \mathbf{I}^2)(\tau) [\left(I_z^1 \cos(\omega_{1,1}\tau) - I_y^1 \sin(\omega_{1,1}\tau)\right) \left(I_z^2 \cos(\omega_{1,2}\tau) - I_y^2 \sin(\omega_{1,2}\tau)\right), I_x^u] \\ &= i\sqrt{2/3} A_{2,0}(\mathbf{I}^1, \mathbf{I}^2)(\tau) \left(I_y^u \cos(\omega_{1,u}\tau) + I_z^u \sin(\omega_{1,u}\tau)\right) \left(I_z^{\tilde{u}} \cos(\omega_{1,\tilde{u}}\tau) - I_y^{\tilde{u}} \sin(\omega_{1,\tilde{u}}\tau)\right).\end{aligned}$$

where $\tilde{u} = 3 - u$ (i.e. $u = 1 \to \tilde{u} = 2$ and $u = 2 \to \tilde{u} = 1$). Acting on $\mathbf{L}_{SB}^{**}(\tau) I_x^u$ with the bath propagator $e^{\mathbf{L}_B \tau}$ introduces a time dependence $A_{kq}(\mathbf{I}^1, \mathbf{I}^2) \to A_{kq}(\mathbf{I}^1, \mathbf{I}^2)(\tau)$. The final expression is:

$$\begin{aligned}&e^{\mathbf{L}_B \tau} \mathbf{L}_{SB}^{**}(\tau) I_x^u \\ &\quad = i\sqrt{2/3} A_{2,0}(\mathbf{I}^1, \mathbf{I}^2)(\tau) \left(I_y^u \cos(\omega_{1,u}\tau) + I_z^u \sin(\omega_{1,u}\tau)\right) \left(I_z^{\tilde{u}} \cos(\omega_{1,\tilde{u}}\tau) - I_y^{\tilde{u}} \sin(\omega_{1,\tilde{u}}\tau)\right).\end{aligned}$$

Next, we compute the inner product by multiplying the "bra" $\langle\langle (\mathbf{L}_{SB}^{**}(0) I_x^u)^\dagger |$ and the "ket" $| e^{i\mathbf{L}_B \tau} \mathbf{L}_{SB}^{**}(\tau) I_x^u \rangle\rangle$,

$$\begin{aligned}\frac{\langle\langle \mathbf{L}_{SB}^{**}(0) I_x^u, e^{\mathbf{L}_B \tau} \mathbf{L}_{SB}^{**}(\tau) I_x^u \rangle\rangle}{\langle\langle I_x^u | I_x^u \rangle\rangle} &= \frac{\mathrm{Tr}\left[(\mathbf{L}_{SB}^{**}(0) I_x^u)^\dagger e^{\mathbf{L}_B \tau} \mathbf{L}_{SB}^{**}(\tau) I_x^u\right]}{\mathrm{Tr}[(I_x^u)^\dagger U_x^u]} \\ &= \frac{2}{3} \langle A_{2,0}(\mathbf{I}^1, \mathbf{I}^2)(0) A_{2,0}(\mathbf{I}^1, \mathbf{I}^2)(\tau) \rangle_B \cos(\omega_{1,u}\tau) \cos(\omega_{1,\tilde{u}}\tau) \cdot \frac{1}{3} I^{\tilde{u}}(I^{\tilde{u}} + 1) \\ &= \frac{1}{3} \langle A_{2,0}(\mathbf{I}^1, \mathbf{I}^2)(0) A_{2,0}(\mathbf{I}^1, \mathbf{I}^2)(\tau) \rangle_B [1 + \cos(2\omega_1 \tau)] \cdot \frac{1}{3} I^{\tilde{u}}(I^{\tilde{u}} + 1),\end{aligned}$$

where we used $\frac{\mathrm{Tr}[(I_y^u)^2(I_z^{\tilde{u}})^2]}{\mathrm{Tr}[(I_x^u)^2]} = \frac{\frac{1}{3}I^u(I^u+1)(2I^u+1)\cdot\frac{1}{3}I^{\tilde{u}}(I^{\tilde{u}}+1)(2I^{\tilde{u}}+1)}{\frac{1}{3}I^u(I^u+1)(2I^u+1)\cdot(2I^{\tilde{u}}+1)} = \frac{1}{3}I^{\tilde{u}}(I^{\tilde{u}}+1)$. The last line follows from the Hartmann-Hahn condition. Commuting with I_y^u, $u = 1, 2$:

$$\begin{aligned}\mathsf{L}_{SB}^{**}(\tau)I_y^u &= \left[\left(I_z^1\cos(\omega_{1,1}\tau) - I_y^1\sin(\omega_{1,1}\tau)\right)\left(I_z^2\cos(\omega_{1,2}\tau) - I_y^2\sin(\omega_{1,2}\tau)\right), I_y^u\right] \\ &= -iI_x^u\cos(\omega_{1,u}\tau)\left(I_z^{\tilde{u}}\cos(\omega_{1,\tilde{u}}\tau) - I_y^{\tilde{u}}\sin(\omega_{1,\tilde{u}}\tau)\right).\end{aligned}$$

Similarly, we find:

$$\frac{\langle\langle \mathsf{L}_{SB}^{**}(0)I_y^u, e^{\mathsf{L}_B\tau}\mathsf{L}_{SB}^{**}(\tau)I_x^u\rangle\rangle}{\langle\langle I_y^u|I_y^u\rangle\rangle} = \frac{1}{3}\langle A_{2,0}(\mathbf{I}^1,\mathbf{I}^2)(0)A_{2,0}(\mathbf{I}^1,\mathbf{I}^2)(\tau)\rangle_B[1+\cos(2\omega_1\tau)]\cdot\frac{1}{3}I^{\tilde{u}}(I^{\tilde{u}}+1).$$

Finally, commutation with I_z^u, $u = 1, 2$:

$$\begin{aligned}\mathsf{L}_{SB}^{**}(\tau)I_z^u &= \left[\left(I_z^1\cos(\omega_{1,1}\tau) - I_y^1\sin(\omega_{1,1}\tau)\right)\left(I_z^2\cos(\omega_{1,2}\tau) - I_y^2\sin(\omega_{1,2}\tau)\right), I_z^u\right] \\ &= -iI_x^u\sin(\omega_{1,u}\tau)\left(I_z^{\tilde{u}}\cos(\omega_{1,\tilde{u}}\tau) - I_y^{\tilde{u}}\sin(\omega_{1,\tilde{u}}\tau)\right)\end{aligned}$$

whereas

$$\frac{\langle\langle \mathsf{L}_{SB}^{**}(0)I_z^u| e^{\mathsf{L}_B\tau}\mathsf{L}_{SB}^{**}(\tau)I_x^u\rangle\rangle}{\langle\langle I_z^u|I_z^u\rangle\rangle)} = 0,$$

because $\sin(0) = 0$. The heteronuclear spin-lock relaxation rate for spin u is:

$$\begin{aligned}\frac{1}{T_{1\rho}^u} &= \sum_j\int_0^\infty \frac{\langle\langle F_k|\mathsf{L}^*(0)e^{i\tau\mathsf{L}_B}\mathsf{L}^*(\tau)|F_j\rangle\rangle}{\langle\langle F_j|F_j\rangle\rangle}d\tau \\ &= \frac{1}{3}\cdot\frac{2}{3}I^{\tilde{u}}(I^{\tilde{u}}+1)\int_0^\infty\langle A_{2,0}(\mathbf{I}^1,\mathbf{I}^2)(0)A_{2,0}(\mathbf{I}^1,\mathbf{I}^2)(\tau)\rangle_B[1+\cos(2\omega_1\tau)]d\tau \\ &= \frac{2}{9}I^{\tilde{u}}(I^{\tilde{u}}+1)[J_{II}(0) + J_{II}^c(2\omega_1)],\end{aligned}$$

which is enantiospecific through the dependence of spectral density functions on chirality.

2.3. **Relationship to the Cross-Polarization Experiment.** We have demonstrated the existence of an effective nuclear spin-spin indirect coupling of the type $\mathbf{I}^1\cdot\mathsf{F}\cdot\mathbf{I}^2$, where the coupling tensor F is enantiospecific. This has consequences for the cross-polarization (CP) experiment. For a more detailed exposé of the theory of CP in solids, the reader can consult Ref. ([10]). We will limit our discussion to the case of cross-polarization in non-rotating solids. In the CP experiment, one considers two nuclei $\mathbf{I}^1$ and $\mathbf{I}^2$, each with their inverse spin temperature (β_{I1}, β_{I2} respectively). The spin temperatures evolve according to the sum of cross-polarization rate (T_{I1-I2}^{-1}) and rotating-frame relaxation ($T_{1\rho}^{-1}$) terms:

$$\dot{\beta}_{I2} = -\frac{1}{T_{I1-I2}}(\beta_{I2}-\beta_{I1}) - \frac{1}{T_{1\rho,I2}}\beta_{I2}$$

$$\dot{\beta}_{I1} = -\frac{1}{T_{I1-I2}}(\beta_{I1}-\beta_{I2}) - \frac{1}{T_{1\rho,I1}}\beta_{I1}.$$

When the initial conditions are $\beta_{I1}(0) = \beta_{I1,0}$ and $\beta_{I2}(0) = 0$, the spin systems evolve according to

$$\beta_{I1}(t) = \beta_{I1,0}\exp(-t/T_{1\rho,I1})$$

$$\beta_{I2}(t) = (\beta_{I1,0}/\lambda)\exp(-t/T_{1\rho,I1})[1-\exp(-t/T_{I1-I2})]$$

where

$$\lambda = 1 + \frac{T_{I^1-I^2}}{T_{1\rho,I2}} - \frac{T_{I1-I2}}{T_{1\rho,I1}}.$$

The I^2 spin magnetization reaches a maximum at time t_m:

$$t_m = \frac{T_{I1-I2} \cdot T_{1\rho,I1}}{T_{1\rho,I1} - T_{I1-I2}} \log \frac{T_{1\rho,I1}}{T_{I1-I2}}.$$

The asymptotic value of I_2 spin magnetization the asymptotic value is

$$\frac{M_{I2}(t \to \infty)}{M_{I2,0}} \cong \frac{\gamma_{I1}}{\gamma_{I2}}.$$

It can be shown that at the Harmann-Hahn condition the CP rate, $(T_{I1-I2})^{-1}$, is:

$$\frac{1}{T_{I1-I2}} = \frac{3}{2} M_2^{I1-I2} \left(\frac{2\pi}{5M_2^{I1}}\right)^{1/2} \cong 1.681 \cdot \frac{M_2^{I1-I2}}{(M_2^{I1})^{1/2}}$$

where M_2^{I1} and M_2^{I1-I2} are the Van Vleck second moments (in units of s^{-2}) for the I^1 and $I^1 - I^2$ spin systems, respectively (see Abragam [9] and Stejskal & Memory [11]). The I^1 spin system generally refers to I^1 spins interacting with the lattice. For example, it could be due to homonuclear I^1- I^1 spin-spin interactions. The $I^1 - I^2$ spin system, on the other hand, refers to the $I^1 - I^2$ heteronuclear interaction, which occurs through the tensor F. The second moment M_2^{I1-I2} is enantiospecific because it is proportional to the trace, $\mathrm{tr}\{[\mathbf{I}^1 \cdot \mathsf{F} \cdot \mathbf{I}^{2**}, I_x]^2\}$, where $I_x = I_x^1 + I_x^2$ and $\mathbf{I}^1 \cdot \mathsf{F} \cdot \mathbf{I}^{2**}$ is the indirect nuclear spin-spin interaction in the spin lock frame ($**$) and the components of the tensor F are enantiospecific (see Section 1.2.1). Therefore, the CP rate $(T_{I^1-I^2})^{-1}$ and time at maximum t_m both depend on chirality through the components of the F tensor.

2.4. **Appendix: Interaction Representation for Spin-Lock Experiment (Review).** This is a review of a well known procedure in quantum mechanics. The usual transformation to the interaction representation proceeds by decomposing the Liouvillian as: $\mathsf{L}(t) = \mathsf{L}_0(t) + \mathsf{L}_V(t)$ (with $'0'$ referring to the unperturbed part and $'V'$ to the perturbation), the Heisenberg equation of motion is

$$\frac{dA(t)}{dt} = \left[\mathsf{L}_0(t) + \mathsf{L}_V(t)\right] A(t).$$

The transformed operators are:

$$\tilde{A} = \overrightarrow{T} \exp\left(-\int_0^t d\tau \mathsf{L}_0(\tau)\right) A, \qquad \tilde{\mathsf{L}} = \overrightarrow{T} \exp\left(-\int_0^t d\tau \mathsf{L}_0(\tau)\right) \mathsf{L},$$

where $\overrightarrow{T}$ is the Dyson time-ordering operator [8] going from left to right, with the most recent (in time) Liouvillian to the right. These transformations are anti-causal. The (total) time-derivative evaluated at the most recent time point is:

$$\begin{aligned}
\frac{d\tilde{A}}{dt} =& \frac{d}{dt} \overrightarrow{T} \exp\left(-\int_0^t d\tau \mathsf{L}_0(\tau)\right) A \\
=& -\overrightarrow{T} \exp\left(-\int_0^t d\tau \mathsf{L}_0(\tau)\right) \mathsf{L}_0(t) A + \overrightarrow{T} \exp\left(-\int_0^t d\tau \mathsf{L}_0(\tau)\right) \left[\mathsf{L}_0(t) A + \mathsf{L}_V(t) A(t)\right] \\
=& \tilde{\mathsf{L}}_V(t) \tilde{A}.
\end{aligned}$$

Thus, in a rotating frame of reference, a Liouville equation

$$\frac{d\tilde{A}}{dt} = \tilde{\mathbf{L}}_V \tilde{A}$$

holds, where $\tilde{\mathbf{L}}_V$ is the perturbation part of the Liouvillian expressed in the rotating frame. It is also possible to effect yet another transformation to a second frame by decomposing the time derivative $\tilde{\mathbf{L}}_V$ further:

$$\tilde{\mathbf{L}}_V = \tilde{\mathbf{L}}_1 + \tilde{\mathbf{L}}_2,$$

where $\tilde{\mathbf{L}}_1$ is the part corresponding to the RF field and $\tilde{\mathbf{L}}_2$ is the remaining "internal" part of the Liouvillian (e.g. spin-spin interactions). Consider the transformation

$$\hat{\tilde{A}} = \overrightarrow{T} \exp\left(-\int_0^t \tilde{\mathbf{L}}_1(t')dt'\right) \tilde{A},$$

where $\overrightarrow{T}$ is the Dyson time-ordering operator [8], which places the most recent events to the right. Then,

$$\frac{d\hat{\tilde{A}}(t)}{dt} = \hat{\tilde{\mathbf{L}}}_2 \hat{\tilde{A}}(t).$$

The solution is,

$$\hat{\tilde{A}}(t) = \overrightarrow{T} \exp\left(\int_0^t \hat{\tilde{\mathbf{L}}}_2(t')dt'\right) \hat{\tilde{A}}(0),$$

where

$$\hat{\tilde{\mathbf{L}}}_2 = \overrightarrow{T} \exp\left(-\int_0^t \tilde{\mathbf{L}}_1(t')dt'\right) \tilde{\mathbf{L}}_2,$$

and

$$\tilde{\mathbf{L}}_2 = \overrightarrow{T} \exp\left(-\int_0^t d\tau \mathbf{L}_0(\tau)\right) \mathbf{L}_2.$$

3. Text S3. J Coupling Stereochemical Deviations (DFT) for Amino Acids

The theoretical investigations into the NMR J couplings in various amino acids were conducted by DFT using the ORCA *ab initio* quantum chemistry software package [12]. The amino acids selected for this study are alanine, arginine, aspartic acid, cysteine, glutamic acid, glutamine, glyceraldehyde (non-amino acid), methionine, phenylalanine, serine, threonine, tyrosine and valine. Differences in NMR J couplings between (D, L) enantiomers were quantified using the J coupling stereochemical deviation, $[J(L) - J(D)]/[J(L) + J(D)]/2$. Nonzero values of this relative difference constitute evidence of chiral selectivity of the scalar coupling. J couplings between ^{1}H and ^{13}C nuclei were computed. The results are shown in Supplementary Figs. 2-13 below. The raw data is provided in the form of tables in Section "Text S4" below.

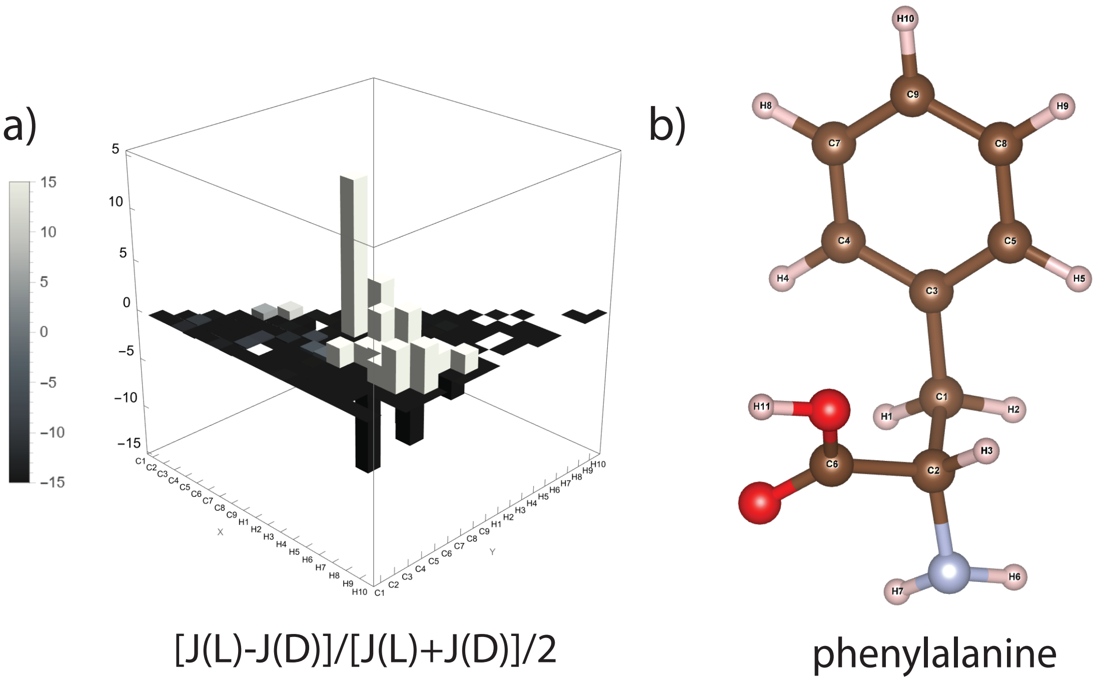

Supplementary Figure 2. J coupling stereochemical deviation, $[J(L) - J(D)]/[J(L) + J(D)]/2$ for phenylalanine. The atom labels are "C1", "C2", "C3", "C4", "C5", "C6","H1", "H2", "H3", "H4", "H5", "H6", "H7", "H8", "H9", "H10", "H11".

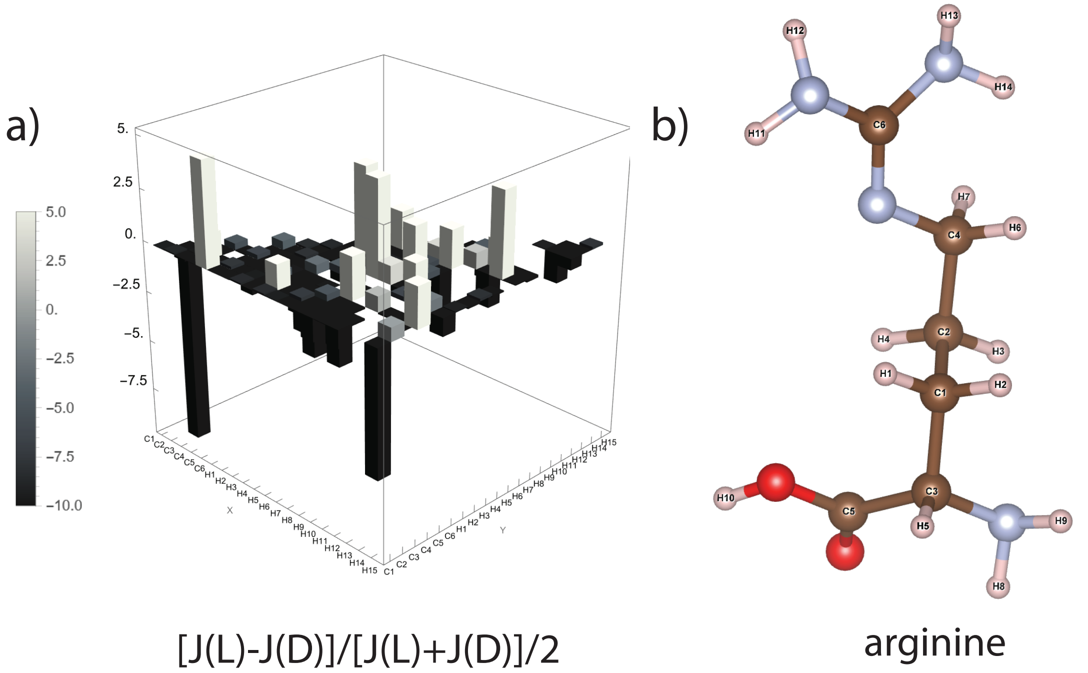

Supplementary Figure 3. J coupling stereochemical deviation, $[J(L) - J(D)]/[J(L) + J(D)]/2$ for arginine. The atom labels are "C1", "C2", "C3", "C4", "C5", "C6", "H1", "H2", "H3", "H4", "H5", "H6", "H7", "H8", "H9", "H10", "H11", "H12", "H13", "H14".

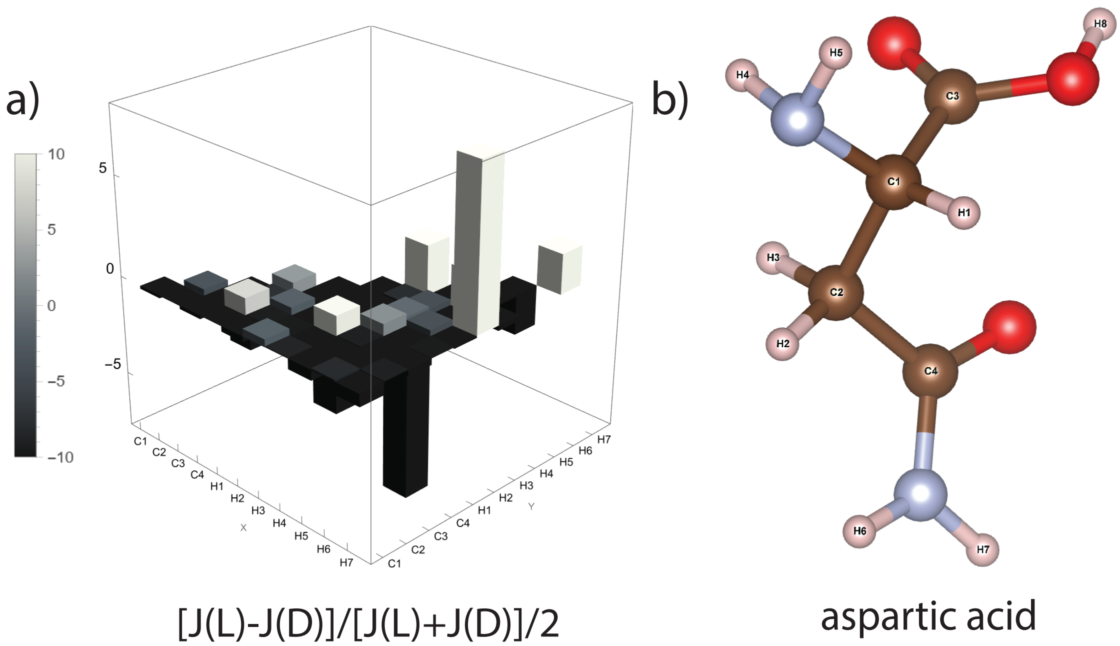

Supplementary Figure 4. J coupling stereochemical deviation, $[J(L) - J(D)]/[J(L) + J(D)]/2$ for aspartic acid. The atoms labels are "C1", "C2", "C3", "C4", "H1", "H2", "H3", "H4", "H5", "H6", "H7".

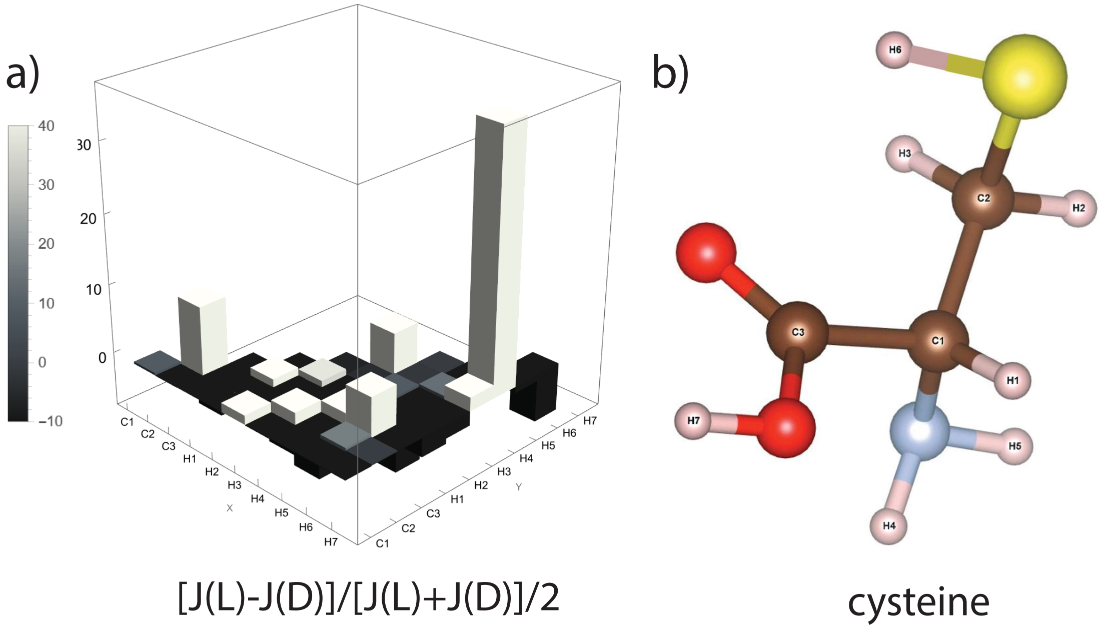

Supplementary Figure 5. J coupling stereochemical deviation, $[J(L) - J(D)]/[J(L) + J(D)]/2$ for cysteine. The atom labels are “C1”, “C2”, “C3”, “H1”, “H2”, “H3”, “H4”, “H5”, “H6”, “H7”.

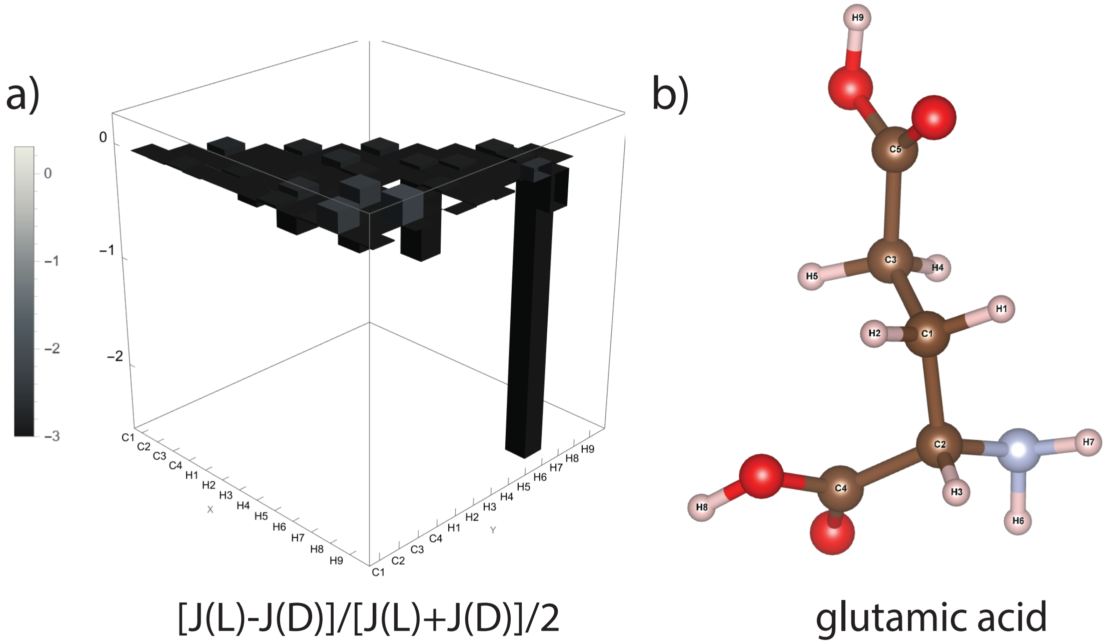

Supplementary Figure 6. J coupling stereochemical deviation, $[J(L) - J(D)]/[J(L) + J(D)]/2$ for glutamic acid. The atom labels are “C1”, “C2”, “C3”, “C4”, “C5”, “H1”, “H2”, “H3”, “H4”, “H5”, “H6”, “H7”, “H8”, “H9”.

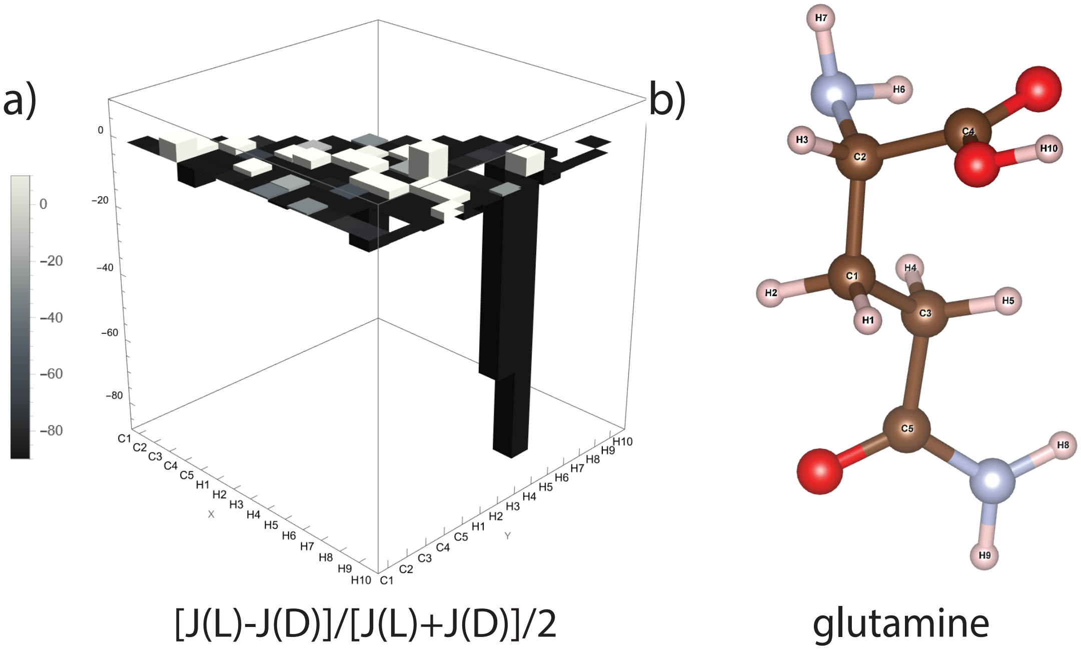

Supplementary Figure 7. J coupling stereochemical deviation, $[J(L) - J(D)]/[J(L) + J(D)]/2$ for glutamine. The atom labels are “C1”, “C2”, “C3”, “C4”, “C5”, “H1”, “H2”, “H3”, “H4”, “H5”, “H6”, “H7”, “H8”, “H9”, “H10”.

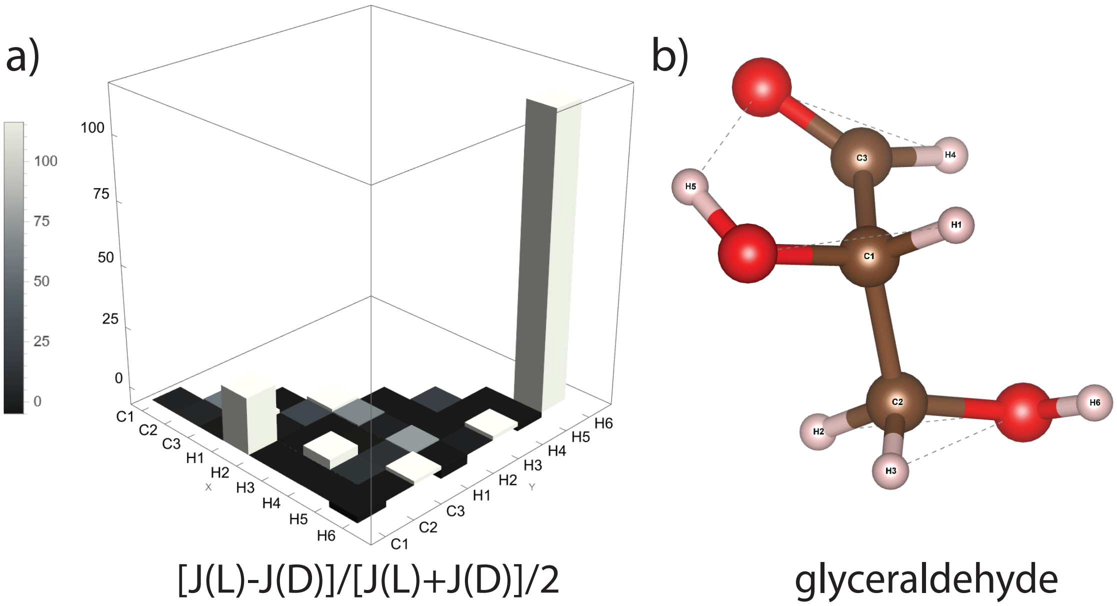

Supplementary Figure 8. J coupling stereochemical deviation, $[J(L) - J(D)]/[J(L) + J(D)]/2$ for glyceraldehyde. The atom labels are “C1”, “C2”, “C3”, “H1”, “H2”, “H3”, “H4”, “H5”, “H6”.

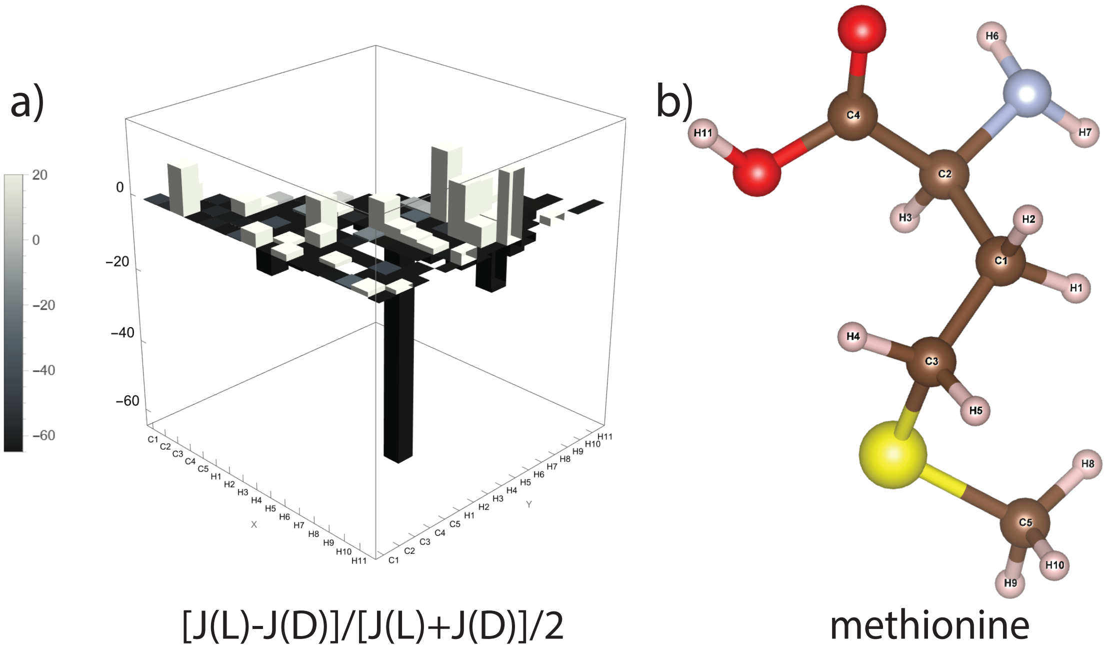

Supplementary Figure 9. J coupling stereochemical deviation, $[J(L) - J(D)]/[J(L) + J(D)]/2$ for methionine. The atom labels are “C1”, “C2”, “C3”, “C4”, “C5”, “H1”, “H2”, “H3”, “H4”, “H5”, “H6”, “H7”, “H8”, “H9”, “H10”, “H11”.

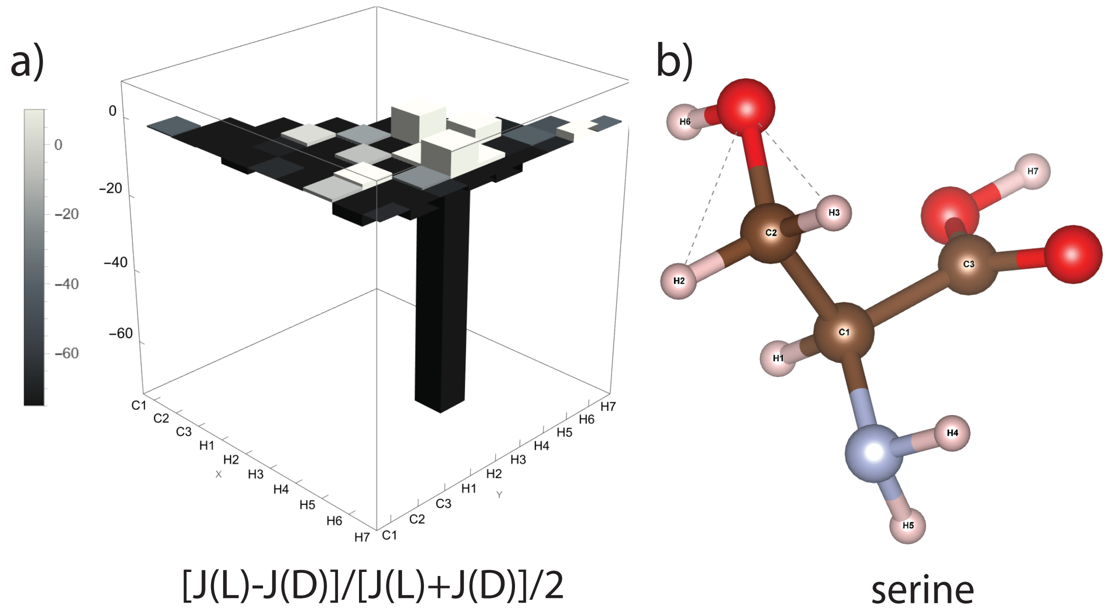

Supplementary Figure 10. J coupling stereochemical deviation, $[J(L) - J(D)]/[J(L) + J(D)]/2$ for serine. The atom labels are “C1”, “C2”, “C3”, “H1”, “H2”, “H3”, “H4”, “H5”, “H6”, “H7”.

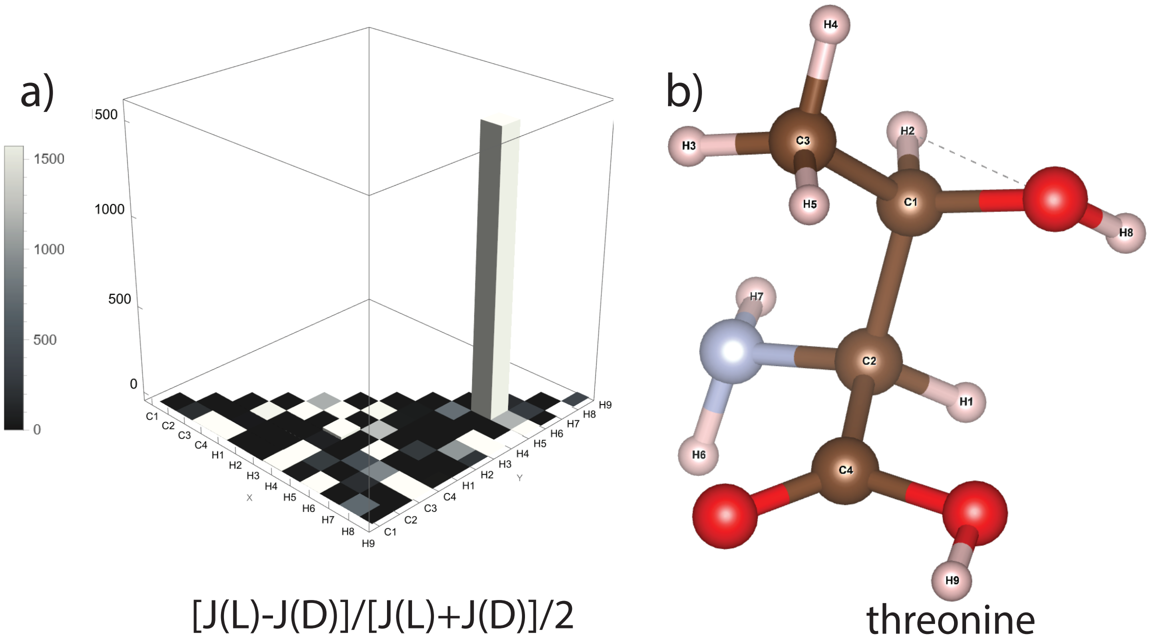

Supplementary Figure 11. J coupling stereochemical deviation, $[J(L) - J(D)]/[J(L) + J(D)]/2$ for threonine. The atom labels are "C1", "C2", "C3", "C4", "H1", "H2", "H3", "H4", "H5", "H6", "H7", "H8", "H9".

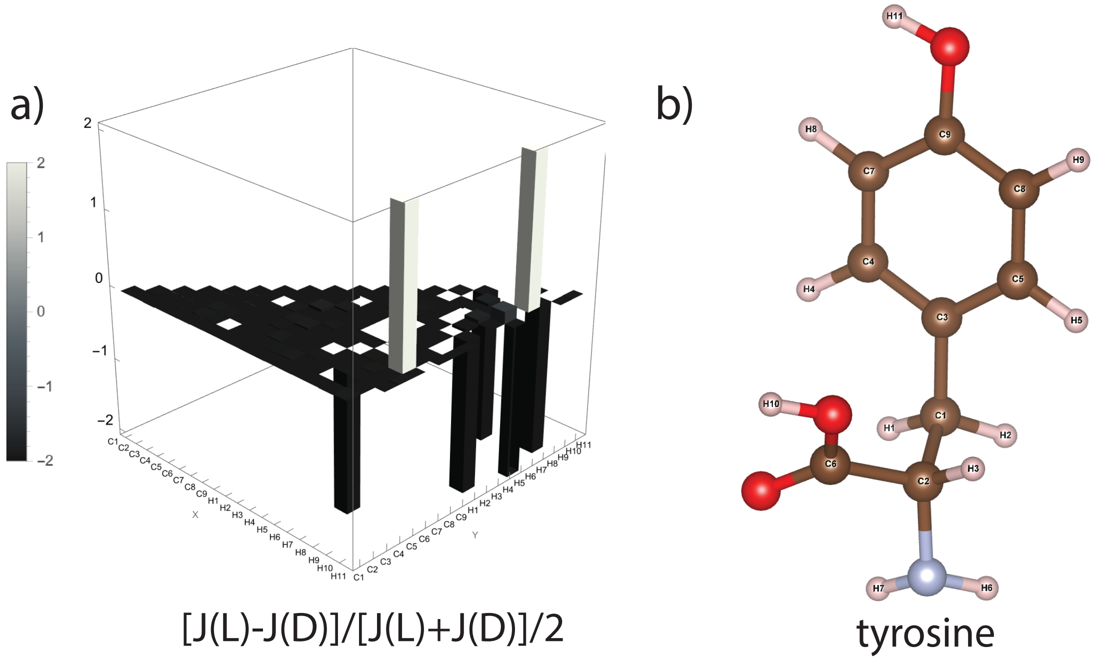

Supplementary Figure 12. J coupling stereochemical deviation, $[J(L) - J(D)]/[J(L) + J(D)]/2$ for tyrosine. The atom labels are "C1", "C2", "C3", "C4", "C5", "C6", "C7", "C8", "C9", "H1", "H2", "H3", "H4", "H5", "H6", "H7", "H8", "H9", "H10", "H11".

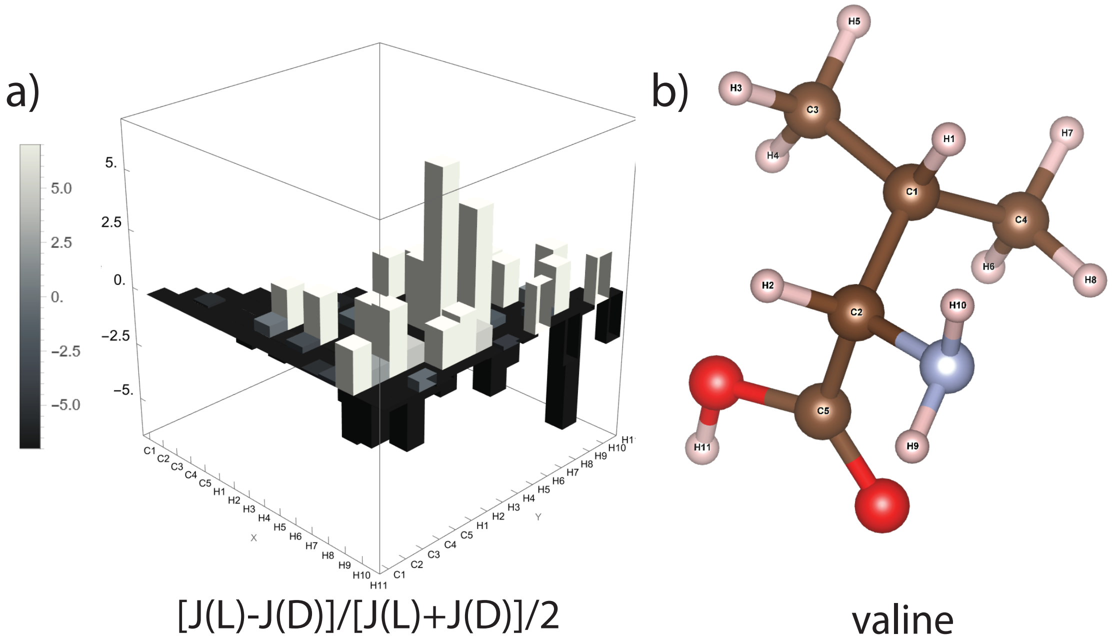

Supplementary Figure 13. J coupling stereochemical deviation, $[J(L) - J(D)]/[J(L) + J(D)]/2$ for valine. The atom labels are "C1", "C2", "C3", "C4", "C5", "H1", "H2", "H3", "H4", "H5", "H6", "H7", "H8", "H9", "H10", "H11".

4. Text S4. Raw Data for J Couplings (DFT) in Amino Acids

This section contains numerical values used to generate plots in the previous section. Raw J coupling values are also provided. Specifically, we provide numerical values for the differences in NMR J couplings between (D, L) enantiomers can be quantified using the J coupling stereochemical deviation, $[J(L) - J(D)]/[J(L) + J(D)]/2$. Nonzero values of this relative difference constitute evidence of chiral selectivity of the scalar coupling. J couplings between ^{1}H and ^{13}C nuclei were computed by DFT using ORCA for the amino acids: alanine, arginine, aspartic acid, cysteine, glutamic acid, glutamine, glyceraldehyde, methionine, phenylalanine, serine, threonine, tyrosine and valine. The results are shown[3] in Supplementary Tables 1-39.

Alanine	C1	C2	C3	H1	H2	H3	H4	H5	H6	H7
C1	0.	-0.13	0.07	0.03	-0.59	0.56	-0.08	-0.31	-0.10	0.13
C2	-0.13	0.	0.08	-0.37	-0.03	-0.01	0.02	-1.11	-5.24	-0.03
C3	0.07	0.08	0.	0.24	-1.67	1.77	-0.02	1.64	0.86	-0.01
H1	0.03	-0.37	0.24	0.	-0.84	0.50	-0.04	-1.88	-0.15	7.39
H2	-0.59	-0.03	-1.67	-0.84	0.	0.06	-0.02	2.17	0.84	-0.49
H3	0.56	-0.01	1.77	0.50	0.06	0.	-0.05	-2.42	-0.47	0.79
H4	-0.08	0.02	-0.02	-0.04	-0.02	-0.05	0.	0.5	1.17	-0.96
H5	-0.31	-1.11	1.64	-1.88	2.17	-2.42	0.5	0.	-0.19	-2.
H6	-0.10	-5.24	0.86	-0.15	0.84	-0.47	1.17	-0.19	0.	-0.70
H7	0.13	-0.03	-0.01	7.39	-0.49	0.79	-0.96	-2.	-0.70	0.

Supplementary Table 1. Numerical values of the fractional deviation $[J(L) - J(D)]/[J(L) + J(D)]/2$ for alanine.

D-alanine	C1	C2	C3	H1	H2	H3	H4	H5	H6	H7
C1	0.	127.25	166.61	169.27	-6.62	-3.6	-6.83	-5.26	-5.96	7.23
C2	127.25	0.	-9.22	-8.51	180.78	178.40	172.88	11.30	1.39	-0.45
C3	166.61	-9.22	0.	-6.96	9.89	0.59	2.30	1.12	1.56	-7.61
H1	169.27	-8.51	-6.96	0.	5.40	3.24	13.68	2.43	15.63	-0.31
H2	-6.62	180.78	9.89	5.40	0.	-13.56	-13.69	0.02	-0.27	-0.15
H3	-3.56	178.40	0.59	3.23	-13.56	0.	-13.90	3.67	-0.40	-0.02
H4	-6.83	172.88	2.30	13.68	-13.69	-13.90	0.	-0.20	-0.04	0.14
H5	-5.26	11.30	1.12	2.43	0.02	3.67	-0.20	0.	-11.71	-0.08
H6	-5.96	1.39	1.56	15.63	-0.27	-0.40	-0.04	-11.71	0.	-0.21
H7	7.23	-0.45	-7.61	-0.31	-0.15	-0.03	0.14	-0.08	-0.21	0.

Supplementary Table 2. Raw values of J couplings in D-alanine.

[3] In the tables, "0." along the diagonal indicates the absence of a J coupling between the same atom (self-coupling). Off-diagonal entries marked with 0. represent indeterminate J-couplings. These values were flagged by ORCA due to limitations in its ability to reliably calculate certain constants under specific molecular conditions. "0.00" indicates a number smaller than 0.01. Also, "−0.01" indicates a similarly small number, but negative.

L-alanine	C1	C2	C3	H1	H2	H3	H4	H5	H6	H7
C1	0.	111.56	179.38	175.18	-3.61	-6.31	-6.30	-3.85	-5.42	8.21
C2	111.56	0.	-9.94	-5.86	175.44	177.06	175.73	3.25	-0.62	-0.44
C3	179.38	-9.94	0.	-8.84	0.90	9.85	2.26	11.27	3.90	-7.56
H1	175.18	-5.86	-8.84	0.	2.22	5.41	13.11	0.08	13.42	0.54
H2	-3.61	175.44	0.90	2.22	0.	-14.38	-13.42	-0.45	-0.67	-0.09
H3	-6.31	177.06	9.85	5.41	-14.38	0.	-13.23	-0.35	-0.25	-0.06
H4	-6.30	175.73	2.26	13.11	-13.42	-13.23	0.	-0.34	-0.16	0.05
H5	-3.85	3.25	11.27	0.08	-0.45	-0.35	-0.34	0.	-9.69	0.
H6	-5.42	-0.62	3.90	13.42	-0.67	-0.25	-0.16	-9.69	0.	-0.10
H7	8.21	-0.44	-7.56	0.54	-0.09	-0.06	0.05	0.	-0.10	0.

Supplementary Table 3. Raw values of J couplings in L-alanine.

Arginine	C1	C2	C3	C4	C5	C6	H1	H2	H3	H4
C1	0.	-0.01	-0.04	-0.01	-0.12	-0.02	-0.03	0.03	0.06	0.00
C2	-0.01	0.	0.21	-0.01	0.72	-0.02	0.01	-0.08	0.01	0.00
C3	-0.04	0.21	0.	-0.27	-0.09	0.	-0.73	0.02	-1.12	0.71
C4	-0.01	-0.01	-0.27	0.	-0.45	-0.03	0.85	-0.61	0.04	-0.01
C5	-0.12	0.72	-0.09	-0.45	0.	0.	-1.11	-0.11	0.06	-1.61
C6	-0.02	-0.02	0.	-0.03	0.	0.	0.18	-0.19	-0.01	-2.
H1	-0.03	0.01	-0.73	0.85	-1.11	0.18	0.	0.04	0.01	-0.52
H2	0.03	-0.08	0.02	-0.61	-0.11	-0.19	0.04	0.	0.52	0.01
H3	0.06	0.01	-1.12	0.04	0.06	-0.01	0.01	0.52	0.	-0.01
H4	0.00	0.00	0.71	-0.01	-1.61	-2.	-0.52	0.01	-0.01	0.
H5	0.44	1.51	-0.01	-1.14	0.61	0.	-1.14	0.22	1.06	0.62
H6	0.02	-0.00	-0.28	0.00	0.	-0.03	-0.20	-0.12	-0.07	0.01
H7	0.00	-0.04	0.04	0.00	0.	-0.00	-0.37	0.77	0.05	-0.02
H8	-1.63	-2.65	0.39	5.06	-1.26	0.	0.57	0.25	4.53	9.64
H9	-0.07	-1.60	0.28	0.	20.	0.	2.31	1.59	-8.78	-1.97
H10	131.67	4.11	-0.16	0.	0.02	0.	-0.34	2.	-2.	-0.21
H11	0.5	-0.08	0.	-0.01	0.	0.02	0.	0.	0.	-0.06
H12	0.	0.	0.	-0.05	0.	0.02	0.	0.	0.	0.
H13	0.	0.	0.	-0.05	0.	-0.00	0.	0.	0.	0.
H14	-6.	-0.03	0.	-0.01	0.	-0.02	-0.06	0.	0.08	-0.09

Arginine	H5	H6	H7	H8	H9	H10	H11	H12	H13	H14
C1	0.44	0.02	0.00	-1.63	-0.07	131.67	0.5	0.	0.	-6.
C2	1.51	-0.00	-0.04	-2.65	-1.60	4.11	-0.08	0.	0.	-0.03
C3	-0.01	-0.28	0.04	0.39	0.28	-0.16	0.	0.	0.	0.
C4	-1.14	0.00	0.00	5.06	0.	0.	-0.01	-0.05	-0.05	-0.01
C5	0.61	0.	0.	-1.26	20.	0.02	0.	0.	0.	0.
C6	0.	-0.03	-0.00	0.	0.	0.	0.02	0.02	-0.00	-0.02
H1	-1.14	-0.20	-0.37	0.57	2.31	-0.34	0.	0.	0.	-0.06
H2	0.22	-0.12	0.77	0.25	1.59	2.	0.	0.	0.	0.
H3	1.06	-0.07	0.05	4.53	-8.78	-2.	0.	0.	0.	0.08
H4	0.62	0.01	-0.02	9.64	-1.97	-0.21	-0.06	0.	0.	-0.09
H5	0.	3.79	-2.	1.94	0.06	-2.32	0.	0.	0.	0.
H6	3.79	0.	0.00	-1.86	0.	0.	-0.01	0.06	0.06	-0.09
H7	-2.	0.00	0.	2.	0.	0.	-0.30	-0.02	-0.07	-0.48
H8	1.94	-1.86	2.	0.	0.21	2.	0.	0.	0.	0.
H9	0.06	0.	0.	0.21	0.	2.48	0.	0.	0.	0.
H10	-2.32	0.	0.	2.	2.48	0.	0.	0.	0.	0.
H11	0.	-0.01	-0.30	0.	0.	0.	0.	0.03	-0.02	-0.01
H12	0.	0.06	-0.02	0.	0.	0.	0.03	0.	0.01	-0.02
H13	0.	0.06	-0.07	0.	0.	0.	-0.02	0.01	0.	0.02
H14	0.	-0.09	-0.48	0.	0.	0.	-0.01	-0.02	0.02	0.

Supplementary Table 4. Numerical values of the fractional deviation $[J(L) - J(D)]/[J(L) + J(D)]/2$ for arginine.

D-arginine	C1	C2	C3	C4	C5	C6	H1	H2	H3	H4
C1	0.	132.23	119.47	-8.47	-9.08	-0.157	176.79	162.55	-6.05	-5.64
C2	132.23	0.	-5.38	143.27	0.66	8.85	-6.57	-6.32	160.57	167.97
C3	119.47	-5.38	0.	8.95	174.26	0.	-7.03	-6.27	2.70	1.11
C4	-8.47	143.27	8.95	0.	-0.131	-4.22	1.04	3.131	-3.61	-7.63
C5	-9.08	0.66	174.26	-0.131	0.	0.	2.44	10.47	-0.17	-0.14
C6	-0.16	8.85	0.	-4.22	0.	0.	0.13	0.11	1.53	-0.06
H1	176.79	-6.57	-7.03	1.04	2.44	0.13	0.	-13.48	13.32	4.58
H2	162.55	-6.32	-6.27	3.13	10.46	0.11	-13.48	0.	2.79	13.11
H3	-6.05	160.57	2.70	-3.61	-0.17	1.53	13.32	2.79	0.	-13.33
H4	-5.64	167.97	1.11	-7.63	-0.14	-0.06	4.58	13.11	-13.33	0.
H5	-4.79	1.13	170.16	-0.17	-4.12	0.	11.32	4.29	-0.15	-0.23
H6	3.59	-5.24	-0.23	153.84	0.	5.06	-0.262	-0.21	2.33	12.
H7	9.67	-4.79	0.90	162.59	0.	3.81	-0.65	-0.14	3.1	3.80
H8	2.65	-0.11	-4.08	-0.01	12.20	0.	-0.28	-0.19	0.012	-0.02
H9	10.09	1.73	-4.12	0.	-0.13	0.	-0.14	-0.03	-0.16	-0.14
H10	0.39	-0.03	8.45	0.	-7.43	0.	-0.06	0.	-0.00	-0.02
H11	0.00	0.06	0.	0.14	0.	-2.93	0.02	0.	0.	-0.02
H12	0.	0.	0.	0.90	0.	-4.68	0.	0.	0.	0.
H13	0.	0.	0.	0.86	0.	-3.60	0.	0.	0.	0.
H14	0.00	-0.12	0.	-0.57	0.	-0.98	-0.02	0.00	0.06	-0.06

D-arginine	H5	H6	H7	H8	H9	H10	H11	H12	H13	H14
C1	-4.79	3.59	9.67	2.65	10.09	0.39	0.00	0.	0.	0.00
C2	1.13	-5.24	-4.79	-0.11	1.73	-0.03	0.06	0.	0.	-0.12
C3	170.16	-0.23	0.904	-4.08	-4.12	8.45	0.	0.	0.	0.
C4	-0.17	153.84	162.59	-0.01	0.	0.	0.14	0.90	0.86	-0.57
C5	-4.12	0.	0.	12.20	-0.13	-7.43	0.	0.	0.	0.
C6	0.	5.06	3.81	0.	0.	0.	-2.93	-4.68	-3.60	-0.98
H1	11.32	-0.26	-0.65	-0.28	-0.14	-0.06	0.02	0.	0.	-0.02
H2	4.29	-0.21	-0.14	-0.19	-0.03	0.	0.	0.	0.	0.00
H3	-0.15	2.33	3.1	0.01	-0.16	-0.00	0.	0.	0.	0.06
H4	-0.23	12.	3.80	-0.02	-0.14	-0.02	-0.018	0.	0.	-0.06
H5	0.	0.013	-0.03	0.23	3.87	1.07	0.	0.	0.	0.
H6	0.01	0.	-14.29	0.03	0.	0.	-0.22	0.76	0.54	-0.44
H7	-0.03	-14.29	0.	0.	0.	0.	-0.06	2.48	0.45	0.08
H8	0.25	0.02	0.	0.	-9.65	0.	0.	0.	0.	0.
H9	3.87	0.	0.	-9.65	0.	0.01	0.	0.	0.	0.
H10	1.07	0.	0.	0.	0.01	0.	0.	0.	0.	0.
H11	0.	-0.22	-0.06	0.	0.	0.	0.	-5.09	0.37	3.71
H12	0.	0.76	2.48	0.	0.	0.	-5.09	0.	-0.43	0.24
H13	0.	0.54	0.45	0.	0.	0.	0.37	-0.43	0.	-3.47
H14	0.	-0.44	0.08	0.	0.	0.	3.71	0.24	-3.47	0.

Supplementary Table 5. Raw values of J couplings in D-arginine.

L-arginine	C1	C2	C3	C4	C5	C6	H1	H2	H3	H4
C1	0.	130.34	114.85	-8.36	-8.05	-0.15	171.75	166.93	-6.43	-5.66
C2	130.34	0.	-6.62	141.14	1.39	8.68	-6.64	-5.86	162.52	168.20
C3	114.85	-6.62	0.	6.84	158.85	0.	-3.25	-6.39	0.77	2.34
C4	-8.36	141.14	6.84	0.	-0.08	-4.08	2.57	1.67	-3.77	-7.59
C5	-8.05	1.39	158.85	-0.08	0.	0.	0.70	9.42	-0.18	-0.02
C6	-0.15	8.68	0.	-4.08	0.	0.	0.16	0.09	1.52	0.
H1	171.75	-6.64	-3.25	2.57	0.70	0.16	0.	-14.04	13.47	2.70
H2	166.93	-5.86	-6.39	1.67	9.42	0.09	-14.04	0.	4.75	13.30
H3	-6.43	162.52	0.77	-3.77	-0.18	1.52	13.47	4.75	0.	-13.19
H4	-5.66	168.20	2.34	-7.59	-0.02	0.	2.70	13.30	-13.19	0.
H5	-7.47	8.07	168.60	-0.05	-7.75	0.	3.10	5.35	-0.48	-0.43
H6	3.66	-5.22	-0.17	154.49	0.	4.90	-0.21	-0.18	2.17	12.12
H7	9.69	-4.59	0.95	162.92	0.	3.80	-0.45	-0.31	3.27	3.71
H8	0.27	0.02	-6.08	0.03	2.78	0.	-0.50	-0.25	-0.03	0.03
H9	9.39	0.19	-5.44	0.	0.154	0.	2.02	-0.28	0.1	-0.00
H10	-0.40	0.08	7.21	0.	-7.56	0.	-0.04	-0.12	0.	-0.02
H11	0.01	0.06	0.	0.142	0.	-2.98	0.02	0.	0.	-0.02
H12	0.	0.	0.	0.85	0.	-4.80	0.	0.	0.	0.
H13	0.	0.	0.	0.82	0.	-3.59	0.	0.	0.	0.
H14	-0.00	-0.12	0.	-0.561	0.	-0.95	-0.017	0.001	0.07	-0.05

L-arginine	H5	H6	H7	H8	H9	H10	H11	H12	H13	H14
C1	-7.47	3.66	9.69	0.27	9.39	-0.40	0.01	0.	0.	-0.00
C2	8.07	-5.22	-4.59	0.02	0.19	0.08	0.06	0.	0.	-0.12
C3	168.60	-0.17	0.95	-6.08	-5.44	7.21	0.	0.	0.	0.
C4	-0.05	154.49	162.92	0.03	0.	0.	0.14	0.85	0.82	-0.56
C5	-7.75	0.	0.	2.78	0.15	-7.56	0.	0.	0.	0.
C6	0.	4.90	3.80	0.	0.	0.	-2.98	-4.80	-3.59	-0.95
H1	3.10	-0.21	-0.45	-0.50	2.02	-0.04	0.02	0.	0.	-0.02
H2	5.35	-0.18	-0.31	-0.25	-0.28	-0.12	0.	0.	0.	0.00
H3	-0.48	2.17	3.27	-0.03	0.1	0.	0.	0.	0.	0.07
H4	-0.43	12.12	3.71	0.03	-0.00	-0.02	-0.02	0.	0.	-0.05
H5	0.	-0.04	0.	15.32	4.11	-0.08	0.	0.	0.	0.
H6	-0.04	0.	-14.30	0.00	0.	0.	-0.22	0.80	0.58	-0.41
H7	0.	-14.30	0.	0.04	0.	0.	-0.05	2.43	0.42	0.05
H8	15.32	0.00	0.04	0.	-11.90	-0.19	0.	0.	0.	0.
H9	4.11	0.	0.	-11.90	0.	-0.122	0.	0.	0.	0.
H10	-0.08	0.	0.	-0.19	-0.12	0.	0.	0.	0.	0.
H11	0.	-0.22	-0.05	0.	0.	0.	0.	-5.23	0.36	3.69
H12	0.	0.80	2.43	0.	0.	0.	-5.23	0.	-0.44	0.24
H13	0.	0.58	0.42	0.	0.	0.	0.36	-0.44	0.	-3.53
H14	0.	-0.41	0.05	0.	0.	0.	3.69	0.24	-3.53	0.

Supplementary Table 6. Raw values of J couplings in L-arginine.

Aspartic acid	C1	C2	C3	C4	H1	H2	H3	H4	H5	H6	H7
C1	0.	-0.07	0.03	-0.22	-0.01	3.67	2.14	-2.24	-2.23	2.08	2.
C2	-0.07	0.	-0.01	-0.01	0.25	0.08	-0.04	1.79	-2.16	-1.30	0.01
C3	0.03	-0.01	0.	-1.90	0.13	-0.91	0.98	-1.02	1.54	0.01	-0.46
C4	-0.22	-0.01	-1.90	0.	0.28	-0.44	0.30	2.46	-2.53	4.19	0.01
H1	-0.01	0.25	0.13	0.28	0.	1.18	-1.15	-2.31	1.40	0.16	-0.12
H2	-0.14	0.08	-0.91	-0.44	1.18	0.	-0.02	1.17	-3.08	2.	22.24
H3	0.17	-0.04	0.98	0.30	-1.15	-0.02	0.	3.13	-0.94	0.48	-17.04
H4	-0.05	1.79	-1.02	2.46	-2.31	1.17	3.13	0.	-0.08	-2.66	0.
H5	-0.09	-2.16	1.54	-2.53	1.40	-3.08	-0.94	-0.08	0.	-2.	0.
H6	0.14	-1.30	0.01	4.19	0.16	2.	0.48	-2.66	-2.	0.	2.
H7	-0.20	0.01	-0.46	0.01	-0.12	22.24	-17.05	0.	0.	2.	0.

Supplementary Table 7. Numerical values of the fractional deviation $[J(L) - J(D)]/[J(L) + J(D)]/2$ for aspartic acid.

D-a. acid	C1	C2	C3	C4	H1	H2	H3	H4	H5	H6	H7
C1	0.	139.48	172.68	-5.65	169.25	1.75	0.25	78.22	80.38	-0.14	0.
C2	139.48	0.	-8.33	188.81	-5.38	163.72	175.59	0.53	11.10	0.97	7.48
C3	172.68	-8.33	0.	0.12	-9.59	10.54	3.08	11.77	1.35	-7.35	-0.15
C4	-5.65	188.81	0.12	0.	1.10	-11.40	-8.46	-0.235	2.98	-0.05	-7.58
H1	169.25	-5.38	-9.589	1.10	0.	2.86	11.08	4.77	1.44	-0.66	-0.11
H2	-6.82	163.72	10.54	-11.40	2.86	0.	-17.85	-0.113	0.79	0.	-0.84
H3	-6.52	175.59	3.08	-8.46	11.08	-17.847	0.	-0.202	-0.48	-0.04	0.74
H4	-4.63	0.53	11.77	-0.24	4.77	-0.113	-0.20	0.	-10.87	-0.25	0.
H5	-4.71	11.10	1.35	2.98	1.44	0.79	-0.48	-10.87	0.	-0.2	0.
H6	6.43	0.97	-7.35	-0.05	-0.66	0.	-0.04	-0.25	-0.2	0.	0.
H7	1.70	7.48	-0.15	-7.58	-0.11	-0.84	0.74	0.	0.	0.	0.

Supplementary Table 8. Raw values of J couplings in D-aspartic acid.

L-a. acid	C1	C2	C3	C4	H1	H2	H3	H4	H5	H6	H7
C1	0.	129.84	177.80	-4.517	168.39	-5.95	-7.76	-4.41	-4.32	7.38	1.39
C2	129.84	0.	-8.26	187.36	-6.90	177.36	168.44	9.42	-0.44	0.20	7.52
C3	177.80	-8.26	0.	0.00	-10.89	3.97	8.973	3.84	10.54	-7.39	-0.09
C4	-4.52	187.36	0.003	0.	1.45	-7.29	-11.41	2.26	-0.35	0.15	-7.66
H1	168.39	-6.90	-10.89	1.45	0.	11.144	2.99	-0.35	8.14	-0.77	-0.09
H2	-5.95	177.36	3.97	-7.29	11.144	0.	-17.58	-0.43	-0.17	-0.07	1.
H3	-7.76	168.44	8.97	-11.41	2.99	-17.58	0.	0.92	-0.17	-0.06	-0.58
H4	-4.41	9.42	3.84	2.26	-0.35	-0.43	0.92	0.	-10.07	0.04	0.
H5	-4.32	-0.44	10.54	-0.35	8.14	-0.17	-0.17	-10.07	0.	0.	0.
H6	7.38	0.20	-7.391	0.147	-0.77	-0.07	-0.06	0.04	0.	0.	-0.04
H7	1.39	7.52	-0.09	-7.66	-0.09	1.	-0.58	0.	0.	-0.04	0.

Supplementary Table 9. Raw values of J couplings in L-aspartic acid.

Cysteine	C1	C2	C3	H1	H2	H3	H4	H5	H6	H7
C1	0.	0.17	-0.03	-0.03	-0.17	0.47	-0.11	0.05	-2.04	-0.28
C2	0.17	0.	-0.05	0.35	-0.03	0.00	1.75	-1.01	0.55	0.27
C3	-0.03	-0.05	0.	0.12	1.33	-0.89	-0.22	-0.02	0.30	-0.08
H1	-0.03	0.35	0.12	0.	-1.23	0.90	-1.63	0.77	6.31	-4.53
H2	-0.17	-0.03	1.33	-1.23	0.	0.07	3.44	-1.22	-0.04	-2.
H3	0.47	0.00	-0.89	0.90	0.07	0.	0.72	0.27	0.44	2.
H4	-0.11	1.75	-0.22	-1.63	3.44	0.72	0.	0.14	-2.	0.97
H5	0.05	-1.01	-0.02	0.77	-1.22	0.27	0.14	0.	-92.67	-4.21
H6	-2.04	0.55	0.30	6.31	-0.04	0.44	-2.	-92.67	0.	3.18
H7	-0.28	0.27	-0.08	-4.53	-2.	2.	0.97	-4.21	3.18	0.

Supplementary Table 10. Numerical values of the fractional deviation $[J(L) - J(D)]/[J(L) + J(D)]/2$ for cysteine.

D-cysteine	C1	C2	C3	H1	H2	H3	H4	H5	H6	H7
C1	0.	124.59	182.12	176.20	-6.93	-3.33	-5.08	-4.191	1.39	8.61
C2	124.59	0.	-7.81	-4.02	191.79	196.98	0.85	1.40	-0.78	0.84
C3	182.12	-7.81	0.	-7.46	2.35	9.22	1.56	11.69	-0.141	-8.00
H1	176.20	-4.02	-7.46	0.	11.54	3.83	15.35	2.02	0.137	1.20
H2	-6.93	191.79	2.35	11.54	0.	-13.87	-0.14	-0.24	14.57	-0.06
H3	-3.33	196.98	9.22	3.83	-13.87	0.	-0.19	-0.10	3.72	0.
H4	-5.08	0.85	1.56	15.35	-0.14	-0.19	0.	-9.07	-0.05	-0.05
H5	-4.19	1.40	11.69	2.02	-0.24	-0.10	-9.07	0.	0.07	0.64
H6	1.39	-0.78	-0.14	0.14	14.57	3.72	-0.05	0.07	0.	-0.03
H7	8.61	0.84	-8.00	1.20	-0.06	0.	-0.05	0.64	-0.03	0.

Supplementary Table 11. Raw values of J couplings in D-cysteine.

L-cysteine	C1	C2	C3	H1	H2	H3	H4	H5	H6	H7
C1	0.	147.52	176.80	170.50	-5.86	-5.40	-4.57	-4.43	-0.01	6.49
C2	147.52	0.	-7.44	-5.73	185.64	197.27	12.98	0.46	-1.37	1.10
C3	176.80	-7.44	0.	-8.38	11.61	3.56	1.248	11.42	-0.19	-7.36
H1	170.50	-5.73	-8.38	0.	2.74	10.15	1.56	4.56	-0.26	-0.46
H2	-5.86	185.64	11.61	2.74	0.	-14.93	0.51	-0.06	14.04	0.
H3	-5.40	197.27	3.56	10.15	-14.93	0.	-0.41	-0.13	5.84	-0.07
H4	-4.57	12.98	1.25	1.56	0.51	-0.41	0.	-10.47	0.	-0.15
H5	-4.43	0.46	11.42	4.56	-0.06	-0.13	-10.47	0.	-0.07	-0.23
H6	-0.01	-1.37	-0.19	-0.26	14.04	5.84	0.	-0.07	0.	0.11
H7	6.49	1.10	-7.36	-0.46	0.	-0.07	-0.15	-0.23	0.11	0.

Supplementary Table 12. Raw values of J couplings in L-cysteine.

Glutamic acid	C1	C2	C3	C4	C5	H1	H2	H3	H4	H5	H6	H7	H8	H9
C1	0.	-0.01	0.01	0.03	-0.03	0.00	0.00	0.02	0.27	-0.28	2.15	-2.14	-0.99	0.02
C2	-0.01	0.	-0.00	-0.00	-0.01	0.522823	-0.55	0.01	1.03	-1.06	0.10	-0.13	-0.00	0.
C3	0.01	-0.00	0.	0.07	0.00	0.06	-0.05	0.01	0.01	-0.01	0.25	-0.32	-3.33	-0.01
C4	0.03	-0.00	0.07	0.	-0.18	1.77	-1.80	-0.02	1.13	-0.94	-0.84	0.85	-0.01	0.
C5	-0.03	-0.01	0.00	-0.18	0.	1.07	-1.12	0.02	0.15	-0.14	-2.	2.	0.	-0.01
H1	0.00	0.52	0.06	1.77	1.07	0.	-0.01	0.54	-0.65	0.03	-16.4	-2.30	-10.07	-0.06
H2	0.00	-0.55	-0.05	-1.80	-1.12	-0.01	0.	-0.53	-0.04	0.62	2.31	13.79	8.73	0.17
H3	0.02	0.01	0.01	-0.02	0.02	0.54	-0.53	0.	0.13	-0.16	-2.19	2.19	-0.06	0.
H4	0.27	1.03	0.01	1.13	0.15	-0.65	-0.04	0.13	0.	0.01	4.14	10.	0.67	6.69
H5	-0.28	-1.06	-0.01	-0.94	-0.14	0.03	0.62	-0.16	0.01	0.	-9.33	-4.86	-1.28	-3.07
H6	2.15	0.10	0.25	-0.85	-2.	-16.4	2.31	-2.19	4.14	-9.33	0.	0.00	2.	0.
H7	-2.14	-0.13	-0.32	0.85	2.	-2.30	13.79	2.19	10.	-4.86	0.00	0.	-2.	0.
H8	-0.99	-0.00	-3.33	-0.01	0.	-10.07	8.73	-0.06	0.67	-1.28	2.	-2.	0.	0.
H9	0.02	0.	-0.01	0.	-0.01	-0.06	0.17	0.	6.69	-3.07	0.	0.	0.	0.

Supplementary Table 13. Numerical values of the fractional deviation $[J(L) - J(D)]/[J(L) + J(D)]/2$ for glutamic acid.

D-glutamic acid	C1	C2	C3	C4	C5	H1	H2	H3	H4	H5	H6	H7	H8	H9
C1	0.	122.22	141.61	-8.23	-5.45	171.17	171.25	-6.04	-5.07	-6.90	-0.28	7.81	0.13	1.71
C2	122.23	0.	-6.88	170.56	8.55	-3.85	-6.56	162.35	0.99	3.07	-3.95	-4.44	7.29	0.
C3	141.61	-6.88	0.	1.43	194.08	-6.64	-7.10	7.19	166.41	167.81	-0.20	-0.25	-0.01	7.01
C4	-8.23	170.56	1.43	0.	-0.15	0.68	11.13	-10.79	-0.04	-0.13	10.34	4.07	-7.59	0.
C5	-5.45	8.55	194.08	-0.15	0.	0.74	2.37	1.10	-9.70	-11.46	-0.02	0.	0.	-7.34
H1	171.17	-3.85	-6.64	0.68	0.74	0.	-15.16	2.76	4.70	12.97	-0.46	2.421	0.18	-0.14
H2	171.25	-6.56	-7.10	11.13	2.37	-15.16	0.	4.82	13.47	2.52	-0.171	0.34	-0.11	-0.13
H3	-6.04	162.35	7.19	-10.79	1.10	2.76	4.82	0.	-0.41	-0.47	7.38	-0.34	-0.79	0.
H4	-5.07	0.99	166.41	-0.04	-9.70	4.70	13.47	-0.41	0.	-19.84	-0.05	-0.03	-0.02	0.36
H5	-6.90	3.07	167.81	-0.13	-11.46	12.97	2.52	-0.47	-19.84	0.	0.05	0.13	-0.04	-0.76
H6	-0.28	-3.95	-0.20	10.34	-0.02	-0.46	-0.17	7.38	-0.05	0.05	0.	-9.27	0.	0.
H7	7.81	-4.44	-0.25	4.07	0.	2.42	0.34	-0.34	-0.03	0.13	-9.27	0.	0.07	0.
H8	0.13	7.29	-0.01	-7.59	0.	0.18	-0.11	-0.79	-0.02	-0.04	0.	0.069	0.	0.
H9	1.71	0.	7.01	0.	-7.34	-0.14	-0.13	0.	0.36	-0.76	0.	0.	0.	0.

Supplementary Table 14. Raw values of J couplings in D-glutamic acid.

L-glutamic acid	C1	C2	C3	C4	C5	H1	H2	H3	H4	H5	H6	H7	H8	H9
C1	0.	121.14	142.46	-8.51	-5.31	171.58	171.37	-6.19	-6.67	-5.18	7.69	-0.26	0.04	1.75
C2	121.15	0.	-6.87	169.81	8.48	-6.58	-3.75	163.40	3.08	0.95	-4.37	-3.90	7.28	0.
C3	142.46	-6.87	0.	1.53	194.15	-7.05	-6.76	7.26	168.25	165.49	-0.26	-0.18	0.00	6.96
C4	-8.51	169.81	1.53	0.	-0.12	11.07	0.59	-10.55	-0.14	-0.05	4.21	10.13	-7.52	0.
C5	-5.31	8.48	194.15	-0.12	0.	2.44	0.67	1.12	-11.31	-9.97	0.	-0.02	0.	-7.25
H1	171.58	-6.58	-7.05	11.07	2.44	0.	-15.06	4.81	2.39	13.42	0.36	-0.17	-0.12	-0.13
H2	171.37	-3.75	-6.76	0.59	0.67	-15.06	0.	2.81	12.92	4.81	2.41	-0.45	0.17	-0.15
H3	-6.19	163.40	7.26	-10.55	1.12	4.81	2.81	0.	-0.47	-0.40	-0.33	7.43	-0.74	0.
H4	-6.67	3.08	168.25	-0.14	-11.31	2.39	12.92	-0.47	0.	-20.06	0.15	0.05	-0.04	-0.67
H5	-5.18	0.95	165.49	-0.05	-9.97	13.42	4.81	-0.40	-20.06	0.	-0.03	-0.06	-0.01	0.16
H6	7.69	-4.37	-0.26	4.21	0.	0.36	2.41	-0.33	0.15	-0.03	0.	-9.31	0.07	0.
H7	-0.26	-3.90	-0.18	10.13	-0.02	-0.17	-0.45	7.43	0.05	-0.06	-9.31	0.	0.	0.
H8	0.04	7.28	0.00	-7.52	0.	-0.12	0.17	-0.74	-0.04	-0.01	0.07	0.	0.	0.
H9	1.75	0.	6.96	0.	-7.25	-0.13	-0.15	0.	-0.67	0.16	0.	0.	0.	0.

Supplementary Table 15. Raw values of J couplings in L-glutamic acid.

Glutamine	C1	C2	C3	C4	C5	H1	H2	H3	H4	H5	H6	H7	H8	H9	H10
C1	0.	0.00	-0.00	-0.00	-0.00	-0.02	0.02	0.01	-0.01	0.02	-2.12	2.12	-0.04	-0.03	0.06
C2	0.00	0.	0.00	0.00	0.01	-0.85	0.86	-0.00	-0.82	0.83	0.15	-0.11	0.	0.	0.00
C3	-0.00	0.00	0.	0.07	0.00	-0.02	0.01	0.01	0.00	0.00	-1.75	2.11	-0.07	0.00	0.21
C4	-0.00	0.00	0.07	0.	0.05	-1.76	1.77	0.02	20.86	-8.88	2.49	-2.53	0.	0.	0.00
C5	-0.00	0.01	0.00	0.05	0.	0.61	-0.55	0.03	-0.14	0.12	2.	-2.	-0.02	0.01	0.
H1	-0.02	-0.85	-0.02	-1.76	0.61	0.	-0.00	-0.72	-0.02	-0.43	0.09	2.55	-0.13	-0.10	-0.14
H2	0.02	0.86	0.01	1.76	-0.55	-0.00	0.	0.72	0.41	0.02	-2.58	-0.13	0.12	0.04	0.21
H3	0.01	-0.00	0.01	0.02	0.03	-0.72	0.72	0.	0.19	-0.19	0.85	-0.83	0.	0.	-0.48
H4	-0.01	-0.82	0.00	20.86	-0.14	-0.02	0.42	0.19	0.	-0.01	6.82	1.28	-0.66	-2.34	-2.
H5	0.02	0.83	0.00	-8.88	0.12	-0.43	0.02	-0.19	-0.01	0.	-1.17	-6.28	0.62	1.48	2.
H6	-2.12	0.15	-1.75	2.49	2.	0.09	-2.58	0.85	6.82	-1.17	0.	0.01	2.	0.	0.02
H7	2.12	-0.11	2.11	-2.53	-2.	2.55	-0.13	-0.83	1.28	-6.28	0.01	0.	-2.	0.	0.02
H8	-0.04	0.	-0.07	0.	-0.02	-0.13	0.12	0.	-0.66	0.62	2.	-2.	0.	0.02	0.
H9	-0.03	0.	0.00	0.	0.01	-0.10	0.03	0.	-2.33	1.48	0.	0.	0.02	0.	0.
H10	0.06	0.00	0.21	0.00	0.	-0.14	0.21	-0.48	-2.	2.	0.02	0.02	0.	0.	0.

Supplementary Table 16. Numerical values of the fractional deviation $[J(L) - J(D)]/[J(L) + J(D)]/2$ for glutamine.

D-glu.	C1	C2	C3	C4	C5	H1	H2	H3	H4	H5	H6	H7	H8	H9	H10
C1	0.	115.62	143.58	-8.22	-5.91	174.57	170.77	-7.46	-6.37	-6.47	8.71	-0.25	0.55	1.85	-0.41
C2	115.62	0.	-6.95	157.71	6.75	-6.62	-2.61	170.04	2.07	0.91	-5.19	-5.89	0.	0.	7.45
C3	143.58	-6.95	0.	1.20	167.80	-7.00	-6.90	8.23	161.94	161.8	0.15	-0.00	-0.56	6.76	0.05
C4	-8.22	157.71	1.20	0.	-0.17	9.85	0.61	-7.80	0.13	-0.17	-0.44	3.92	0.	0.	-7.71
C5	-5.91	6.75	167.80	-0.17	0.	1.12	2.01	0.40	-9.38	-8.61	0.	0.05	3.18	-2.77	0.
H1	174.57	-6.62	-7.00	9.85	1.12	0.	-15.26	5.93	13.98	4.73	-0.37	-0.20	-0.06	-0.15	-0.08
H2	170.77	-2.61	-6.90	0.61	2.01	-15.26	0.	2.714	3.09	13.70	1.68	-0.40	-0.05	-0.14	-0.08
H3	-7.46	170.04	8.23	-7.80	0.40	5.93	2.71	0.	-0.42	-0.50	5.65	14.01	0.	0.	0.16
H4	-6.37	2.07	161.94	0.13	-9.38	13.98	3.09	-0.42	0.	-18.06	-0.04	0.01	-0.33	-0.71	-0.02
H5	-6.47	0.91	161.8	-0.17	-8.61	4.73	13.70	-0.50	-18.06	0.	0.05	0.06	-0.20	-0.13	0.
H6	8.71	-5.19	0.15	-0.44	0.	-0.37	1.68	5.65	-0.04	0.05	0.	-11.82	0.	0.	-0.16
H7	-0.25	-5.89	-0.00	3.92	0.05	-0.20	-0.40	14.01	0.01	0.06	-11.82	0.	0.01	0.	-0.17
H8	0.55	0.	-0.56	0.	3.18	-0.06	-0.05	0.	-0.33	-0.20	0.	0.014	0.	4.81	0.
H9	1.85	0.	6.76	0.	-2.77	-0.15	-0.14	0.	-0.71	-0.13	0.	0.	4.81	0.	0.
H10	-0.41	7.45	0.05	-7.71	0.	-0.08	-0.08	0.16	-0.021	0.	-0.16	-0.17	0.	0.	0.

Supplementary Table 17. Raw values of J couplings in D-glutamine.

L-glu.	C1	C2	C3	C4	C5	H1	H2	H3	H4	H5	H6	H7	H8	H9	H10
C1	0.	115.91	143.14	-8.20	-5.89	171.51	174.04	-7.51	-6.30	-6.57	-0.26	8.68	0.53	1.80	-0.43
C2	115.91	0.	-6.97	157.75	6.81	-2.66	-6.52	169.61	0.87	2.20	-6.02	-5.26	0.	0.	7.47
C3	143.14	-6.97	0.	1.28	167.90	-6.88	-6.95	8.30	162.22	161.92	0.01	0.11	-0.52	6.77	0.06
C4	-8.20	157.75	1.28	0.	-0.18	0.62	9.73	-7.93	-0.16	0.11	4.01	-0.46	0.	0.	-7.71
C5	-5.89	6.81	167.90	-0.18	0.	2.1	1.14	0.41	-8.194	-9.69	0.05	0.	3.12	-2.78	0.
H1	171.51	-2.66	-6.88	0.62	2.1	0.	-15.22	2.79	13.65	3.07	-0.41	1.68	-0.05	-0.14	-0.07
H2	174.04	-6.52	-6.95	9.73	1.14	-15.22	0.	5.79	4.71	13.99	-0.21	-0.36	-0.06	-0.14	-0.09
H3	-7.51	169.61	8.30	-7.93	0.41	2.79	5.79	0.	-0.50	-0.41	13.98	5.76	0.	0.	0.10
H4	-6.30	0.87	162.22	-0.16	-8.19	13.65	4.71	-0.50	0.	-17.88	0.08	0.05	-0.16	0.06	0.
H5	-6.57	2.20	161.92	0.11	-9.69	3.07	13.99	-0.41	-17.88	0.	0.01	-0.03	-0.38	-0.86	-0.02
H6	-0.26	-6.02	0.01	4.01	0.05	-0.41	-0.21	13.98	0.08	0.01	0.	-11.97	0.01	0.	-0.17
H7	8.68	-5.26	0.11	-0.46	0.	1.68	-0.36	5.76	0.05	-0.03	-11.97	0.	0.	0.	-0.17
H8	0.53	0.	-0.52	0.	3.12	-0.05	-0.06	0.	-0.16	-0.38	0.01	0.	0.	4.93	0.
H9	1.80	0.	6.77	0.	-2.78	-0.14	-0.142	0.	0.06	-0.86	0.	0.	4.93	0.	0.
H10	-0.43	7.47	0.06	-7.71	0.	-0.07	-0.09	0.10	0.	-0.02	-0.17	-0.17	0.	0.	0.

Supplementary Table 18. Raw values of J couplings in L-glutamine.

Glyceraldehyde	C1	C2	C3	H1	H2	H3	H4	H5	H6
C1	0.	-0.01	0.04	0.02	1.84	-0.01	-0.06	-0.01	-4.99
C2	-0.01	0.	0.05	0.86	0.04	0.00	7.37	0.01	-0.04
C3	0.04	0.05	0.	-0.10	0.30	-0.33	0.03	0.14	1.13
H1	0.02	0.86	-0.10	0.	1.57	0.55	-0.02	0.64	-1.35
H2	1.84	0.04	0.30	1.57	0.	0.01	0.27	-2.69	-0.06
H3	-0.01	0.00	-0.33	0.55	0.01	0.	0.21	0.03	1.52
H4	-0.06	7.37	0.03	-0.02	0.27	0.21	0.	-0.03	-6.
H5	-0.01	0.01	0.14	0.64	-2.69	0.03	-0.03	0.	-4.65
H6	-4.99	-0.04	1.13	-1.35	-0.06	1.52	-6.	-4.65	0.

Supplementary Table 19. Numerical values of the fractional deviation $[J(L) - J(D)]/[J(L) + J(D)]/2$ for glyceraldehyde.

D-glyceraldeyde	C1	C2	C3	H1	H2	H3	H4	H5	H6
C1	0.	133.69	152.46	170.72	-0.19	-5.11	25.77	-3.87	1.54
C2	133.69	0.	-8.32	-3.82	177.45	187.05	0.16	3.54	-4.82
C3	152.46	-8.32	0.	-6.39	2.57	10.64	223.53	3.88	-0.07
H1	170.72	-3.82	-6.40	0.	1.37	3.84	-0.66	1.57	-0.70
H2	-0.19	177.45	2.57	1.37	0.	-13.24	-0.04	1.69	15.05
H3	-5.11	187.05	10.64	3.84	-13.24	0.	-0.35	-0.20	0.71
H4	25.77	0.16	223.53	-0.66	-0.04	-0.35	0.	1.51	-0.07
H5	-3.87	3.54	3.88	1.57	1.69	-0.20	1.511	0.	0.23
H6	1.54	-4.82	-0.07	-0.70	15.05	0.71	-0.072	0.23	0.

Supplementary Table 20. Raw values of J couplings in D-glyceraldehyde.

L-glyceraldehyde	C1	C2	C3	H1	H2	H3	H4	H5	H6
C1	0.	132.82	158.41	174.11	-4.69	-5.08	24.36	-3.84	-0.66
C2	132.82	0.	-8.78	-9.53	184.95	187.88	-0.28	3.59	-4.65
C3	158.41	-8.78	0.	-5.81	3.48	7.66	231.02	4.46	-0.25
H1	174.11	-9.53	-5.81	0.	11.40	6.73	-0.65	3.05	-0.14
H2	-4.69	184.95	3.48	11.40	0.	-13.35	-0.05	-0.25	14.18
H3	-5.08	187.88	7.66	6.73	-13.35	0.	-0.43	-0.21	5.20
H4	24.36	-0.28	231.02	-0.65	-0.05	-0.43	0.	1.47	0.04
H5	-3.84	3.59	4.46	3.05	-0.25	-0.21	1.47	0.	-0.09
H6	-0.66	-4.65	-0.25	-0.14	14.18	5.20	0.04	-0.09	0.

Supplementary Table 21. Raw values of J couplings in L-glyceraldehyde.

Methionine	C1	C2	C3	C4	C5	H1	H2	H3
C1	0.	0.21	0.00	-0.33	0.60	0.07	-0.06	0.56
C2	0.21	0.	-0.36	-0.02	0.25	0.03	0.01	-0.07
C3	0.00	-0.36	0.	1.79	-0.15	0.68	-0.63	1.11
C4	-0.33	-0.02	1.79	0.	0.	0.76	-0.69	0.23
C5	0.60	0.25	-0.15	0.	0.	0.57	0.29	-1.06
H1	0.07	0.03	0.68	0.76	0.57	0.	0.10	-1.70
H2	-0.06	0.01	-0.63	-0.69	0.29	0.10	0.	0.90
H3	0.56	-0.07	1.11	0.23	-1.06	10.68	-2.	2.
H4	0.78	-1.34	0.00	-0.31	0.67	1.28	0.38	-1.25
H5	-0.83	1.17	0.02	1.78	-1.09	0.29	-1.37	-22.77
H6	0.51	-0.13	-3.31	0.76	0.	-0.33	-0.65	-0.26
H7	0.87	0.07	6.36	-2.10	2.14	-19.80	0.90	1.72
H8	0.17	0.13	-0.35	2.	0.01	1.04	48.	-2.28
H9	-1.87	2.	-0.07	0.	0.01	2.	-2.	0.04
H10	0.53	-0.77	0.30	0.	0.00	-0.02	-2.51	2.11
H11	1.42	-0.20	2.60	-0.02	0.	2.56	-1.07	-2.36
Methionine	H4	H5	H6	H7	H8	H9	H10	H11
C1	0.78	-0.83	0.51	0.87	0.17	-1.87	0.53	1.42
C2	-1.34	1.17	-0.13	0.07	0.13	2.	-0.77	-0.20
C3	0.00	0.02	-3.31	6.36	-0.35	-0.07	0.30	2.60
C4	-0.31	1.78	0.76	-2.10	2.	0.	0.	-0.02
C5	0.67	-1.09	0.	2.14	0.01	0.01	0.00	0.
H1	1.28	0.29	-0.33	-19.80	1.04	2.	-0.02	2.56
H2	0.38	-1.37	-0.65	0.90	48.	-2.	-2.51	-1.07
H3	2.77	2.25	-2.04	1.72	-2.28	0.04	2.11	-2.36
H4	0.	-0.05	-3.36	7.78	8.86	3.14	2.52	0.75
H5	-0.05	0.	-2.	-2.	-4.55	-4.92	-16.81	-0.03
H6	-3.36	-2.	0.	0.19	2.	0.	0.	-0.03
H7	7.78	-2.	0.19	0.	2.	0.	-2.	2.
H8	8.86	-4.55	2.	2.	0.	-0.01	0.03	0.
H9	3.14	-4.92	0.	0.	-0.01	0.	-0.04	0.
H10	2.52	-16.81	0.	-2.	0.03	-0.04	0.	0.
H11	0.75	-0.03	-0.03	2.	0.	0.	0.	0.

Supplementary Table 22. Numerical values of the fractional deviation $[J(L) - J(D)]/[J(L) + J(D)]/2$ for methionine.

D-methionine	C1	C2	C3	C4	C5	H1	H2	H3
C1	0.	104.42	138.28	-8.18	0.84	167.30	172.23	-4.79
C2	104.42	0.	-7.22	174.89	-0.12	-6.04	-6.06	178.22
C3	138.28	-7.22	0.	0.08	-1.07	-3.99	-8.52	1.48
C4	-8.18	174.89	0.08	0.	0.	2.57	8.86	-8.26
C5	0.84	-0.12	-1.07	0.	0.	0.13	0.22	0.03
H1	167.30	-6.04	-3.99	2.57	0.13	0.	-14.71	11.12
H2	172.23	-6.06	-8.52	8.86	0.22	-14.71	0.	3.78
H3	-4.79	178.22	1.48	-8.26	0.03	11.12	3.78	0.
H4	-3.20	8.9	189.61	-0.31	4.28	3.31	2.86	-0.68
H5	-5.72	2.39	188.46	-0.01	8.10	2.61	14.33	-0.16
H6	-0.29	-5.61	-0.40	2.81	0.	-0.16	-0.29	14.91
H7	2.82	-4.05	-0.25	11.83	0.00	-0.27	-0.28	0.72
H8	0.34	0.15	2.5	0.	202.67	-0.04	0.05	-0.14
H9	-0.21	0.	9.93	0.	198.20	0.	-0.11	-0.11
H10	0.29	0.17	1.55	0.	203.18	0.06	-0.16	0.01
H11	-0.07	8.17	-0.08	-7.63	0.	0.02	0.18	0.84

D-methionine	H4	H5	H6	H7	H8	H9	H10	H11
C1	-3.20	-5.72	-0.29	2.82	0.34	-0.21	0.29	-0.07
C2	8.9	2.39	-5.61	-4.05	0.15	0.	0.17	8.17
C3	189.61	188.46	-0.40	-0.25	2.5	9.93	1.55	-0.08
C4	-0.31	-0.01	2.81	11.83	0.	0.	0.	-7.63
C5	4.28	8.09	0.	0.00	202.67	198.20	203.18	0.
H1	3.31	2.61	-0.16	-0.27	-0.04	0.	0.06	0.02
H2	2.86	14.33	-0.29	-0.28	0.05	-0.11	-0.16	0.18
H3	-0.68	-0.16	14.91	0.72	-0.14	-0.11	0.01	0.84
H4	0.	-15.79	-0.08	0.04	0.49	-0.30	0.05	0.08
H5	-15.79	0.	0.03	-0.00	-0.22	1.38	-0.76	0.19
H6	-0.08	0.03	0.	-9.43	0.	0.	0.	-0.09
H7	0.03	-0.00	-9.43	0.	0.	0.	0.06	0.
H8	0.49	-0.22	0.	0.	0.	-14.78	-9.90	0.
H9	-0.30	1.38	0.	0.	-14.78	0.	-14.80	0.
H10	0.05	-0.76	0.	0.06	-9.90	-14.80	0.	0.
H11	0.08	0.19	-0.09	0.	0.	0.	0.	0.

Supplementary Table 23. Raw values of J couplings in D-methionine.

L-methionine	C1	C2	C3	C4	C5	H1	H2	H3
C1	0.	128.33	138.70	-5.89	1.56	179.24	162.75	-8.54
C2	128.33	0.	-5.00	171.07	-0.16	-6.21	-6.11	165.85
C3	138.70	-5.00	0.	1.35	-0.91	-8.12	-4.42	5.16
C4	-5.89	171.07	1.35	0.	0.	5.72	4.30	-10.46
C5	1.56	-0.16	-0.91	0.	0.	0.24	0.30	0.01
H1	179.24	-6.21	-8.12	5.72	0.24	0.	-16.249	0.9
H2	162.75	-6.11	-4.42	4.30	0.30	-16.25	0.	10.03
H3	-8.54	165.85	5.16	-10.46	0.01	-16.25	0.	10.03
H4	-7.26	1.76	190.25	-0.23	8.57	14.96	4.189	-0.16
H5	-2.37	9.12	191.77	-0.11	2.37	3.49	2.69	0.14
H6	-0.48	-4.91	0.10	6.22	0.	-0.11	-0.15	11.50
H7	7.15	-4.34	0.48	-0.29	-0.03	0.22	-0.75	9.41
H8	0.41	0.17	1.75	0.06	205.70	-0.12	-0.05	0.01
H9	-0.01	-0.01	9.30	0.	200.39	-0.08	0.	-0.12
H10	0.50	0.08	2.11	0.	203.82	0.06	0.02	-0.25
H11	-0.43	6.71	0.59	-7.48	0.	-0.16	0.05	-0.07

L-methionine	H4	H5	H6	H7	H8	H9	H10	H11
C1	-7.26	-2.37	-0.48	7.15	0.41	-0.01	0.50	-0.43
C2	1.76	9.12	-4.91	-4.34	0.17	-0.01	0.08	6.71
C3	190.25	191.77	0.10	0.48	1.75	9.30	2.11	0.59
C4	-0.23	-0.11	6.22	-0.29	0.06	0.	0.	-7.48
C5	8.57	2.37	0.	-0.03	205.70	200.39	203.82	0.
H1	14.96	3.49	-0.11	0.22	-0.12	-0.08	0.06	-0.16
H2	4.19	2.69	-0.15	-0.75	-0.05	0.	0.02	0.05
H3	4.19	2.69	-0.15	9.41	0.01	-0.12	-0.25	-0.07
H4	0.	-14.97	0.02	-0.07	-0.77	1.34	-0.46	0.18
H5	-14.97	0.	0.	0.	0.08	-0.58	0.60	0.19
H6	0.02	0.	0.	-11.40	0.06	0.	0.	-0.09
H7	-0.07	0.	-11.40	0.	0.07	0.	0.	-0.20
H8	-0.77	0.08	0.06	0.07	0.	-14.64	-10.25	0.
H9	1.34	-0.58	0.	0.	-14.64	0.	-14.17	0.
H10	-0.46	0.60	0.	0.	-10.25	-14.17	0.	0.
H11	0.18	0.19	-0.09	-0.20	0.	0.	0.	0.

Supplementary Table 24. Raw values of J couplings in L-methionine.

Phenylalanine	C1	C2	C3	C4	C5	C6	C7	C8	C9	H1
C1	0.	-0.01	-0.00	-0.24	0.24	0.00	0.02	-0.02	0.	0.01
C2	-0.01	0.	0.00	0.15	-0.15	0.00	-0.06	0.06	0.	-0.04
C3	-0.00	0.00	0.	-0.00	0.00	-0.02	-0.04	0.04	0.00	-0.18
C4	-0.24	0.15	-0.00	0.	-0.01	0.53	-0.01	-0.03	0.04	0.09
C5	0.24	-0.15	0.00	-0.01	0.	-0.51	0.03	0.01	-0.04	0.16
C6	0.00	0.00	-0.02	0.53	-0.51	0.	-0.11	0.14	0.	-1.26
C7	0.02	-0.06	-0.04	-0.01	0.03	-0.11	0.	-0.00	0.00	-1.45
C8	-0.02	0.06	0.04	-0.03	0.01	0.14	-0.00	0.	-0.00	1.13
C9	0.	0.	0.00	0.04	-0.04	0.	0.00	-0.00	0.	-0.93
H1	0.01	-0.04	-0.18	0.09	0.16	-1.26	-1.45	1.13	-0.93	0.
H2	-0.01	0.03	0.18	-0.16	-0.09	1.27	-1.13	1.47	0.92	-0.00
H3	0.02	0.00	0.03	-0.13	0.12	0.00	-0.44	0.42	0.	0.72
H4	0.02	0.23	0.27	0.01	0.03	0.78	-0.07	-0.01	0.01	-0.43
H5	-0.01	-0.24	-0.27	-0.03	-0.01	-0.81	0.02	0.06	-0.01	-1.06
H6	1.54	-0.25	-0.35	0.	-0.23	1.36	0.	0.	0.	0.15
H7	-1.55	0.24	0.35	0.23	0.	-1.36	0.	0.	0.	-0.34
H8	0.05	0.	-0.00	-0.14	0.02	2.	0.	0.00	0.01	-0.53
H9	-0.05	0.	0.00	-0.02	0.15	-2.	-0.00	-0.00	-0.02	0.
H10	0.	0.	0.	-0.01	0.02	0.	0.06	-0.06	-0.00	0.
H11	-0.08	-0.00	0.2	-0.52	0.55	0.00	2.	-2.	0.	2.

Phenylalanine	H2	H3	H4	H5	H6	H7	H8	H9	H10	H11
C1	-0.01	0.02	0.02	-0.01	1.54	-1.55	0.05	-0.05	0.	-0.08
C2	0.03	0.00	0.23	-0.24	-0.25	0.24	0.	0.	0.	-0.00
C3	0.18	0.03	0.27	-0.27	-0.35	0.35	-0.00	0.00	0.	0.2
C4	-0.16	-0.13	0.01	-0.03	0.	0.23	-0.14	-0.02	-0.01	-0.52
C5	-0.09	0.12	0.03	-0.01	-0.23	0.	0.02	0.15	0.02	0.55
C6	1.27	0.00	0.78	-0.81	1.36	-1.36	2.	-2.	0.	0.00
C7	-1.13	-0.44	-0.07	0.02	0.	0.	0.	-0.00	0.06	2.
C8	1.47	0.42	-0.01	0.06	0.	0.	0.00	-0.00	-0.06	-2.
C9	0.92	0.	0.01	-0.01	0.	0.	0.01	-0.02	-0.00	0.
H1	-0.00	0.72	-0.43	-1.06	0.15	-0.34	-0.53	0.	0.	2.
H2	0.	-0.72	1.06	0.44	0.34	-0.15	0.	0.525	0.	-2.
H3	-0.72	0.	-0.42	0.36	-1.75	1.75	-2.	2.	0.	-0.01
H4	1.06	-0.42	0.	-0.00	0.	3.33	-0.00	-0.02	0.04	2.
H5	0.44	0.36	-0.00	0.	-3.	0.	0.02	0.00	-0.04	-2.
H6	0.34	-1.75	0.	-3.	0.	-0.01	0.	0.	0.	-2.
H7	-0.15	1.75	3.33	0.	-0.01	0.	0.	0.	0.	2.
H8	0.	-2.	-0.00	0.02	0.	0.	0.	0.	0.00	2.
H9	0.53	2.	-0.02	0.00	0.	0.	0.	0.	-0.00	-2.
H10	0.	0.	0.04	-0.04	0.	0.	0.00	-0.00	0.	0.
H11	-2.	-0.01	2.	-2.	-2.	2.	2.	-2.	0.	0.

Supplementary Table 25. Numerical values of the fractional deviation $[J(L) - J(D)]/[J(L) + J(D)]/2$ for phenylalanine.

D-ph.	C1	C2	C3	C4	C5	C6	C7	C8	C9	H1
C1	0.	94.48	153.16	-4.33	-3.40	-7.30	3.51	3.58	-1.17	170.41
C2	94.48	0.	-7.72	2.10	2.44	177.46	-1.40	-1.32	0.	-7.13
C3	153.16	-7.72	0.	249.36	248.63	0.54	-3.35	-3.22	8.65	-7.99
C4	-4.33	2.10	249.36	0.	-3.88	-0.05	275.77	8.16	-3.16	4.07
C5	-3.40	2.44	248.63	-3.88	0.	-0.09	7.96	273.85	-3.28	6.31
C6	-7.30	177.46	0.54	-0.05	-0.09	0.	0.04	0.03	0.	9.04
C7	3.51	-1.40	-3.35	275.77	7.96	0.04	0.	-2.20	265.94	-0.63
C8	3.58	-1.32	-3.22	8.16	273.85	0.03	-2.20	0.	267.09	0.28
C9	-1.17	0.	8.65	-3.16	-3.28	0.	265.94	267.09	0.	0.87
H1	170.41	-7.13	-7.99	4.07	6.31	9.04	-0.63	0.28	0.87	0.
H2	172.12	-6.90	-6.71	7.45	4.47	2.03	1.02	-0.09	0.32	-12.93
H3	-2.84	178.18	0.69	-0.18	-0.16	-7.68	0.12	0.08	0.	5.18
H4	3.47	-0.65	-0.84	210.66	6.52	0.04	-2.03	-1.35	7.64	-0.57
H5	3.53	-0.82	-1.12	6.69	213.73	0.09	-1.33	-1.90	7.74	-0.44
H6	0.32	-5.68	-0.40	0.	-0.05	2.31	0.	0.	0.	-0.29
H7	2.48	-4.44	-0.28	-0.04	0.	12.03	0.	0.	0.	-0.28
H8	0.62	0.	7.73	-1.71	-1.70	0.	216.41	7.88	-1.40	0.20
H9	0.65	0.	7.71	-1.73	-1.47	0.00	7.92	216.46	-1.41	0.
H10	0.	0.	-1.79	7.80	7.69	0.	-1.13	-1.20	218.17	0.
H11	0.30	8.55	-0.01	0.05	0.03	-7.83	0.	0.03	0.04	0.

D-ph.	H2	H3	H4	H5	H6	H7	H8	H9	H10	H11
C1	172.12	-2.84	3.47	3.53	0.32	2.48	0.62	0.65	0.	0.30
C2	-6.90	178.18	-0.65	-0.82	-5.68	-4.44	0.	0.	0.	8.55
C3	-6.71	0.69	-0.84	-1.12	-0.40	-0.28	7.73	7.71	-1.79	-0.01
C4	7.45	-0.18	210.66	6.69	0.	-0.04	-1.71	-1.73	7.80	0.05
C5	4.47	-0.16	6.52	213.73	-0.05	0.	-1.70	-1.47	7.69	0.03
C6	2.03	-7.68	0.04	0.09	2.31	12.03	0.	0.00	0.	-7.83
C7	1.02	0.12	-2.03	-1.33	0.	0.	216.41	7.92	-1.13	0.
C8	-0.09	0.08	-1.35	-1.90	0.	0.	7.88	216.46	-1.20	0.03
C9	0.32	0.	7.63	7.74	0.	0.	-1.40	-1.41	218.17	0.04
H1	-12.93	5.18	-0.57	-0.44	-0.29	-0.28	0.20	0.	0.	0.
H2	0.	10.97	-0.13	-0.37	-0.20	-0.34	0.	0.12	0.	-0.06
H3	10.97	0.	0.06	0.03	15.00	1.01	-0.03	0.	0.	1.16
H4	-0.13	0.06	0.	1.86	0.	0.00	6.75	0.75	1.11	0.
H5	-0.37	0.03	1.86	0.	-0.01	0.	0.74	6.74	1.16	-0.02
H6	-0.20	15.00	0.	-0.01	0.	-9.67	0.	0.	0.	-0.07
H7	-0.34	1.01	0.00	0.	-9.67	0.	0.	0.	0.	0.
H8	0.	-0.03	6.75	0.74	0.	0.	0.	1.28	6.59	0.
H9	0.12	0.00	0.75	6.74	0.00	0.	1.28	0.	6.61	0.01
H10	0.	0.	1.11	1.16	0.	0.	6.59	6.61	0.	0.
H11	-0.06	1.16	0.	-0.02	-0.07	0.	0.	0.00	0.	0.

Supplementary Table 26. Raw values of J couplings in D-phenylalanine.

L-ph.	C1	C2	C3	C4	C5	C6	C7	C8	C9	H1
C1	0.00	94.00	153.04	-3.41	-4.33	-7.33	3.58	3.51	-1.17	172.23
C2	93.99	0.00	-7.75	2.43	2.10	177.57	-1.32	-1.40	0.00	-6.86
C3	153.04	-7.75	0.00	248.85	249.40	0.53	-3.23	-3.35	8.65	-6.65
C4	-3.41	2.43	248.85	0.00	-3.85	-0.09	273.88	7.95	-3.28	4.46
C5	-4.33	2.10	249.40	-3.85	0.00	-0.06	8.16	275.73	-3.16	7.44
C6	-7.33	177.57	0.53	-0.09	-0.06	0.00	0.03	0.04	0.00	2.06
C7	3.57	-1.32	-3.23	273.88	8.16	0.03	0.00	-2.20	267.17	-0.10
C8	3.51	-1.40	-3.35	7.95	275.73	0.04	-2.20	0.00	266.00	1.02
C9	-1.17	0.00	8.65	-3.28	-3.16	0.00	267.17	266.00	0.00	0.32
H1	172.23	-6.86	-6.65	4.46	7.44	2.06	-0.10	1.02	0.32	0.00
H2	170.49	-7.14	-8.00	6.33	4.07	9.02	0.28	-0.63	0.87	-12.92
H3	-2.89	178.20	0.71	-0.16	-0.18	-7.68	0.08	0.12	0.00	10.95
H4	3.53	-0.82	-1.10	213.75	6.69	0.08	-1.89	-1.33	7.73	-0.37
H5	3.48	-0.64	-0.84	6.52	210.70	0.04	-1.35	-2.02	7.63	-0.13
H6	2.46	-4.41	-0.28	0.00	-0.04	12.03	0.00	0.00	0.00	-0.33
H7	0.31	-5.67	-0.39	-0.05	0.00	2.30	0.00	0.00	0.00	-0.20
H8	0.65	0.00	7.71	-1.48	-1.72	0.00	216.40	7.92	-1.41	0.12
H9	0.61	0.00	7.72	-1.70	-1.72	0.00	7.89	216.41	-1.39	0.00
H10	0.00	0.00	-1.78	7.69	7.80	0.00	-1.19	-1.13	218.16	0.00
H11	0.28	8.54	-0.01	0.03	0.05	-7.83	0.03	0.00	0.04	-0.05

L-ph.	H2	H3	H4	H5	H6	H7	H8	H9	H10	H11
C1	170.49	-2.89	3.53	3.48	2.46	0.31	0.65	0.61	0.00	0.28
C2	-7.14	178.20	-0.82	-0.64	-4.41	-5.67	0.00	0.00	0.00	8.54
C3	-8.00	0.71	-1.10	-0.84	-0.28	-0.39	7.71	7.72	-1.78	-0.01
C4	6.33	-0.16	213.75	6.52	0.00	-0.05	-1.48	-1.70	7.69	0.03
C5	4.07	-0.18	6.69	210.70	-0.04	0.00	-1.72	-1.72	7.80	0.05
C6	9.02	-7.68	0.08	0.03	12.03	2.30	0.00	0.00	0.00	-7.83
C7	0.28	0.08	-1.89	-1.35	0.00	0.00	216.40	7.89	-1.19	0.02
C8	-0.63	0.12	-1.33	-2.02	0.00	0.00	7.92	216.41	-1.13	0.00
C9	0.87	0.00	7.73	7.63	0.00	0.00	-1.41	-1.39	218.16	0.03
H1	-12.92	10.95	-0.37	-0.13	-0.33	-0.20	0.12	0.00	0.00	-0.06
H2	0.00	5.14	-0.43	-0.57	-0.28	-0.29	0.00	0.20	0.00	0.00
H3	5.14	0.00	0.04	0.06	1.01	15.02	0.00	-0.03	0.00	1.15
H4	-0.43	0.04	0.00	1.85	0.00	-0.00	6.73	0.73	1.15	-0.02
H5	-0.57	0.06	1.85	0.00	0.00	0.00	0.74	6.74	1.11	0.00
H6	-0.28	1.01	0.00	0.00	0.00	-9.58	0.00	0.00	0.00	0.00
H7	-0.29	15.03	-0.00	0.00	-9.58	0.00	0.00	0.00	0.00	-0.08
H8	0.00	0.00	6.73	0.74	0.00	0.00	0.00	1.29	6.61	0.00
H9	0.20	-0.03	0.73	6.74	0.00	0.00	1.29	0.00	6.59	0.00
H10	0.00	0.00	1.15	1.11	0.00	0.00	6.61	6.59	0.00	0.00
H11	0.00	1.15	-0.02	0.00	0.00	-0.08	0.00	0.00	0.00	0.00

Supplementary Table 27. Raw values of J couplings in L-phenylalanine.

Serine	C1	C2	C3	H1	H2	H3	H4	H5	H6	H7
C1	0.00	0.17	-0.06	-0.04	0.54	0.06	0.09	0.51	-1.65	0.14
C2	0.17	0.00	-0.18	-0.00	0.04	-0.01	-2.24	1.14	-0.01	-2.91
C3	-0.06	-0.18	0.00	-0.57	0.88	-1.26	-0.38	-2.04	0.27	0.08
H1	-0.04	-0.00	-0.57	0.00	-0.48	0.78	0.11	2.14	-1.45	2.13
H2	0.54	0.04	0.88	-0.48	0.00	0.03	0.23	0.74	1.61	1.83
H3	0.06	-0.01	-1.26	0.78	0.03	0.00	-0.44	-0.51	0.07	-128.00
H4	0.09	-2.24	-0.38	0.11	0.23	-0.44	0.00	0.25	-0.22	0.56
H5	0.51	1.14	-2.04	2.14	0.74	-0.51	0.25	0.00	1.34	2.00
H6	-1.65	-0.01	0.27	-1.45	1.61	0.07	-0.22	1.34	0.00	-1.21
H7	0.14	-2.91	0.08	2.13	1.83	-128.00	0.56	2.00	-1.21	0.00

Supplementary Table 28. Numerical values of the fractional deviation $[J(L) - J(D)]/[J(L) + J(D)]/2$ for serine.

D-serine	C1	C2	C3	H1	H2	H3	H4	H5	H6	H7
C1	0.00	104.06	180.41	176.84	-1.52	-5.54	-4.93	-2.84	1.24	6.61
C2	104.06	0.00	-9.32	-8.32	178.77	184.34	-1.25	3.72	-4.49	-0.44
C3	180.41	-9.32	0.00	-8.88	3.18	9.28	4.48	10.84	-0.07	-7.22
H1	176.84	-8.32	-8.88	0.00	10.24	4.99	12.81	-0.16	-0.27	-0.02
H2	-1.52	178.77	3.18	10.24	0.00	-12.13	-0.15	-0.23	0.19	0.01
H3	-5.54	184.34	9.28	4.99	-12.13	0.00	-0.20	-0.36	14.93	0.06
H4	-4.93	-1.25	4.48	12.81	-0.15	-0.20	0.00	-8.57	-0.03	-0.09
H5	-2.84	3.72	10.84	-0.16	-0.23	-0.36	-8.57	0.00	-0.04	0.00
H6	1.24	-4.49	-0.07	-0.27	0.19	14.93	-0.03	-0.04	0.00	-0.16
H7	6.61	-0.44	-7.22	-0.02	0.01	0.06	-0.09	0.00	-0.16	0.00

Supplementary Table 29. Raw values of J couplings in D-serine.

L-serine	C1	C2	C3	H1	H2	H3	H4	H5	H6	H7
C1	0.00	123.13	170.37	170.10	-2.65	-5.86	-5.42	-4.77	0.12	7.64
C2	123.13	0.00	-7.76	-8.31	186.71	182.41	0.07	13.50	-4.46	0.08
C3	170.37	-7.76	0.00	-4.93	8.17	2.11	3.06	-0.11	-0.09	-7.81
H1	170.10	-8.31	-4.93	0.00	6.25	11.44	14.34	4.69	-0.04	0.60
H2	-2.65	186.71	8.17	6.25	0.00	-12.44	-0.19	-0.51	1.80	0.32
H3	-5.86	182.41	2.11	11.44	-12.44	0.00	-0.13	-0.22	15.99	-0.06
H4	-5.42	0.07	3.06	14.34	-0.19	-0.13	0.00	-11.04	-0.02	-0.16
H5	-4.77	13.50	-0.11	4.69	-0.51	-0.22	-11.04	0.00	-0.21	-0.04
H6	0.12	-4.46	-0.09	-0.04	1.80	15.99	-0.02	-0.21	0.00	-0.04
H7	7.64	0.08	-7.81	0.60	0.32	-0.06	-0.16	-0.04	-0.04	0.00

Supplementary Table 30. Raw values of J couplings in L-serine.

Threonine	C1	C2	C3	C4	H1	H2	H3	H4	H5	H6	H7	H8	H9
C1	0.00	0.11	0.05	-2.42	-5.61	-0.02	-0.76	1.62	-0.18	3.75	1.64	-0.91	-1.55
C2	0.11	0.00	0.62	0.29	5.01	0.74	-1.69	1.98	0.85	-0.07	1.72	-1.60	0.20
C3	0.05	0.62	0.00	3.13	-0.58	0.22	0.03	0.01	-0.01	-0.36	-1.80	1.81	2.00
C4	-2.42	0.29	3.13	0.00	-8.38	3.58	-2.30	-0.95	-1.90	0.64	-1.53	-2.05	0.68
H1	-5.61	5.01	-0.58	-8.38	0.00	2.24	-2.15	-1.17	3.56	-0.53	0.93	-1.17	0.72
H2	-0.02	0.74	0.22	3.58	2.24	0.00	-1.08	1.31	0.06	-1.51	0.05	-0.87	1.43
H3	-0.76	-1.69	0.03	-2.30	-2.15	-1.08	0.00	-0.04	-0.16	3.49	3.12	2.63	2.00
H4	1.62	1.98	0.01	-0.95	-1.17	1.31	-0.04	0.00	-0.01	-1.09	3.24	-1.81	0.00
H5	-0.18	0.85	-0.01	-1.90	3.56	0.06	-0.16	-0.01	0.00	-0.95	-25.36	-1.14	2.00
H6	3.75	-0.07	-0.36	0.64	-0.53	-1.51	3.49	-1.09	-0.95	0.00	-0.09	-1.38	-1.97
H7	1.64	1.72	-1.80	-1.53	0.93	0.05	3.12	3.24	-25.36	-0.09	0.00	8.91	2.00
H8	-0.91	-1.60	1.81	-2.05	-1.17	-0.87	2.63	-1.81	-1.14	-1.38	8.91	0.00	-0.79
H9	-1.55	0.20	2.00	0.68	0.72	1.43	2.00	0.00	2.00	-1.97	2.00	-0.79	0.00

Supplementary Table 31. Numerical values of the fractional deviation $[J(L) - J(D)]/[J(L) + J(D)]/2$ for threonine.

D-threonine	C1	C2	C3	C4	H1	H2	H3	H4	H5	H6	H7	H8	H9
C1	0.00	97.40	141.88	79.49	17.04	186.12	-6.07	-0.76	-9.84	-0.47	0.42	-11.15	-2.34
C2	97.40	0.00	-3.99	129.45	-71.73	-1.28	6.63	0.05	1.24	-4.85	-0.32	6.55	5.69
C3	141.88	-3.99	0.00	-0.45	11.35	-4.19	170.84	172.58	176.55	-0.14	-0.41	0.48	0.00
C4	79.49	129.45	-0.45	0.00	13.77	-2.45	1.54	-0.53	2.34	3.31	10.45	10.95	-3.89
H1	17.04	-71.73	11.35	13.77	0.00	-0.32	2.39	4.59	0.11	10.56	3.54	-0.35	0.13
H2	186.12	-1.28	-4.19	-2.45	-0.32	0.00	5.68	1.01	11.81	-0.18	-0.34	1.92	0.03
H3	-6.07	6.63	170.84	1.54	2.39	5.68	0.00	-14.95	-15.74	-0.02	-0.05	-0.54	0.00
H4	-0.76	0.05	172.58	-0.53	4.59	1.01	-14.95	0.00	-13.96	0.08	0.03	-0.90	0.00
H5	-9.84	1.24	176.55	2.34	0.11	11.81	-15.74	-13.96	0.00	-0.14	-0.17	-0.50	0.00
H6	-0.47	-4.85	-0.14	3.31	10.56	-0.18	-0.02	0.08	-0.14	0.00	-10.99	-0.38	-0.14
H7	0.42	-0.32	-0.41	10.45	3.54	-0.34	-0.05	0.03	-0.17	-10.99	0.00	0.04	0.00
H8	-11.15	6.55	0.48	10.95	-0.35	1.92	-0.54	-0.90	-0.50	-0.38	0.04	0.00	-0.21
H9	-2.34	5.69	0.00	-3.89	0.13	0.03	0.00	0.00	0.00	-0.14	0.00	-0.21	0.00

Supplementary Table 32. Raw values of J couplings in D-threonine.

L-threonine	C1	C2	C3	C4	H1	H2	H3	H4	H5	H6	H7	H8	H9
C1	0.00	108.73	149.19	-7.48	-8.08	183.24	-2.73	-7.20	-8.21	1.54	4.27	-4.15	-0.30
C2	108.73	0.00	-7.60	172.85	166.93	-2.76	0.55	9.38	3.06	-4.54	-4.21	0.72	6.97
C3	149.19	-7.60	0.00	2.05	6.21	-5.23	175.27	174.38	174.24	-0.09	-0.02	9.74	0.06
C4	-7.48	172.85	2.05	0.00	-8.46	8.64	-0.11	-0.19	0.06	6.46	1.38	-0.14	-7.87
H1	-8.08	166.93	6.21	-8.46	0.00	5.52	-0.08	1.21	-0.39	6.13	9.69	-0.09	0.27
H2	183.24	-2.76	-5.23	8.64	5.52	0.00	1.69	4.83	12.52	-0.03	-0.35	0.76	0.17
H3	-2.73	0.55	175.27	-0.11	-0.08	1.69	0.00	-14.32	-13.45	0.08	0.24	3.96	-0.05
H4	-7.20	9.38	174.38	-0.19	1.21	4.83	-14.32	0.00	-13.79	0.02	-0.14	-0.05	0.00
H5	-8.21	3.06	174.24	0.06	-0.39	12.52	-13.45	-13.79	0.00	-0.05	0.15	-0.14	-0.02
H6	1.54	-4.54	-0.09	6.46	6.13	-0.03	0.08	0.02	-0.05	0.00	-10.00	-0.07	-0.00
H7	4.27	-4.21	-0.02	1.38	9.69	-0.35	0.24	-0.14	0.15	-10.00	0.00	-0.06	-0.14
H8	-4.15	0.72	9.74	-0.14	-0.09	0.76	3.96	-0.05	-0.14	-0.07	-0.06	0.00	-0.09
H9	-0.30	6.97	0.06	-7.87	0.27	0.17	-0.05	0.00	-0.02	-0.00	-0.14	-0.09	0.00

Supplementary Table 33. Raw values of J couplings in L-threonine.

Tyrosine	C1	C2	C3	C4	C5	C6	C7	C8	C9	H1
C1	0.00	-0.00	-0.00	-0.23	0.23	0.01	0.02	-0.02	0.00	0.01
C2	-0.00	0.00	-0.00	0.20	-0.20	-0.00	-0.10	0.09	0.00	-0.01
C3	-0.00	-0.00	0.00	0.01	-0.00	-0.03	-0.03	0.03	0.00	-0.17
C4	-0.23	0.20	0.01	0.00	-0.01	0.67	0.01	0.02	5.13	0.10
C5	0.23	-0.20	-0.00	-0.01	0.00	-0.70	-0.02	-0.01	-5.12	0.16
C6	0.01	-0.00	-0.03	0.67	-0.70	0.00	0.06	-0.05	0.00	-1.27
C7	0.02	-0.10	-0.03	0.01	-0.02	0.06	0.00	-0.00	-0.09	-1.22
C8	-0.02	0.09	0.03	0.02	-0.01	-0.05	0.00	0.00	0.09	1.03
C9	0.00	0.00	0.00	5.13	-5.11	0.00	-0.09	0.09	0.00	-0.94
H1	0.01	-0.01	-0.17	0.10	0.16	-1.27	-1.22	1.03	-0.94	0.00
H2	-0.01	0.00	0.16	-0.16	-0.10	1.27	-1.07	1.19	0.92	-0.00
H3	0.02	0.00	-0.00	-0.11	0.10	0.00	-0.36	0.36	0.00	0.73
H4	0.03	0.26	0.41	0.02	0.02	0.79	0.07	0.00	0.01	-0.42
H5	-0.02	-0.28	-0.42	-0.02	-0.01	-0.81	-0.00	-0.08	-0.01	-1.11
H6	1.38	-0.24	-0.30	0.00	-0.27	1.40	0.00	0.00	0.00	0.18
H7	-1.44	0.24	0.28	0.25	0.00	-1.38	0.00	0.00	0.00	-0.34
H8	-0.08	0.00	-0.08	0.16	-0.06	2.00	-0.04	-0.05	-0.43	-0.50
H9	0.09	0.00	0.08	0.06	-0.16	-2.00	0.05	0.04	0.43	0.00
H10	-0.09	-0.00	0.10	-0.49	0.51	-0.01	2.00	-2.00	0.00	2.00
H11	0.00	0.00	0.00	-2.09	2.09	0.00	-1.32	1.32	0.00	0.00

Tyrosine	H2	H3	H4	H5	H6	H7	H8	H9	H10	H11
C1	-0.01	0.02	0.03	-0.02	1.38	-1.44	-0.08	0.09	-0.09	0.00
C2	0.00	0.00	0.26	-0.28	-0.24	0.24	0.00	0.00	-0.00	0.00
C3	0.16	-0.00	0.41	-0.42	-0.30	0.28	-0.08	0.08	0.10	0.00
C4	-0.16	-0.11	0.02	-0.02	0.00	0.25	0.16	0.06	-0.49	-2.09
C5	-0.10	0.10	0.02	-0.01	-0.27	0.00	-0.06	-0.16	0.51	2.09
C6	1.27	0.00	0.79	-0.81	1.40	-1.38	2.00	-2.00	-0.00	0.00
C7	-1.07	-0.36	0.07	-0.00	0.00	0.00	-0.04	0.05	2.00	-1.32
C8	1.19	0.36	0.00	-0.08	0.00	0.00	-0.05	0.04	-2.00	1.32
C9	0.92	0.00	0.01	-0.01	0.00	0.00	-0.43	0.43	0.00	0.00
H1	-0.00	0.73	-0.42	-1.12	0.18	-0.34	-0.50	0.00	2.00	0.00
H2	0.00	-0.73	1.09	0.43	0.34	-0.19	0.00	0.49	-2.00	0.00
H3	-0.73	0.00	-0.32	0.32	-1.73	1.72	-2.00	2.00	-0.01	0.00
H4	1.09	-0.32	0.00	-0.00	0.00	3.33	-0.04	0.09	2.00	2.00
H5	0.44	0.32	-0.00	0.00	-3.33	0.00	-0.09	0.04	-2.00	-2.00
H6	0.34	-1.73	0.00	-3.33	0.00	-0.01	0.00	0.00	-2.00	0.00
H7	-0.19	1.72	3.33	0.00	-0.01	0.00	0.00	0.00	2.00	0.00
H8	-0.50	0.00	-2.00	-0.04	-0.09	0.00	0.00	0.00	0.00	0.87
H9	0.49	2.00	0.09	0.04	0.00	0.00	0.00	0.00	0.00	-0.90
H10	-2.00	-0.01	2.00	-2.00	-2.00	2.00	0.00	0.00	0.00	0.00
H11	0.00	0.00	2.00	-2.00	0.00	0.00	0.87	-0.90	0.00	0.00

Supplementary Table 34. Numerical values of the fractional deviation $[J(L) - J(D)]/[J(L) + J(D)]/2$ for tyrosine.

D-tyrosine	C1	C2	C3	C4	C5	C6	C7	C8	C9	H1
C1	0.	95.80	155.50	-3.92	-3.13	-7.19	3.78	3.85	-1.20	169.93
C2	95.80	0.	-7.67	2.04	2.48	177.62	-1.39	-1.26	0.	-6.94
C3	155.50	-7.67	0.	250.04	251.19	0.58	-3.69	-3.59	8.58	-8.04
C4	-3.92	2.04	250.04	0.	-3.26	-0.06	289.06	5.78	0.79	4.01
C5	-3.13	2.48	251.19	-3.26	0.	-0.12	5.90	291.00	-1.82	6.38
C6	-7.19	177.62	0.58	-0.06	-0.12	0.	0.04	0.04	0.	9.14
C7	3.78	-1.39	-3.69	289.06	5.90	0.04	0.	3.27	286.75	-0.66
C8	3.85	-1.26	-3.59	5.78	291.00	0.04	3.27	0.	262.26	0.31
C9	-1.20	0.	8.58	0.79	-1.82	0.	286.75	262.26	0.	0.80
H1	169.93	-6.94	-8.04	4.01	6.38	9.14	-0.66	0.31	0.80	0.
H2	171.68	-6.92	-6.84	7.50	4.42	2.04	0.95	-0.17	0.30	-13.25
H3	-2.73	177.65	0.71	-0.18	-0.16	-7.62	0.12	0.08	0.	5.08
H4	3.29	-0.65	-1.07	209.42	7.43	0.04	-1.93	-1.59	9.50	-0.55
H5	3.37	-0.85	-1.62	7.62	212.69	0.09	-1.60	-2.07	9.60	-0.44
H6	0.45	-5.62	-0.41	0.	-0.05	2.14	0.	0.	0.	-0.28
H7	2.41	-4.43	-0.30	-0.04	0.	12.06	0.	0.	0.	-0.27
H8	0.46	0.	7.79	-2.82	-1.14	0.	219.59	4.75	-4.10	0.21
H9	0.42	0.	7.20	-1.07	-3.30	0.00	4.51	210.91	-2.64	0.
H10	0.36	8.47	-0.01	0.05	0.03	-7.86	0.	0.02	0.	0.
H11	0.	0.	-0.40	1.88	-0.04	0.	9.13	1.88	-4.70	0.

D-tyrosine	H2	H3	H4	H5	H6	H7	H8	H9	H10	H11
C1	171.68	-2.73	3.29	3.37	0.45	2.41	0.46	0.42	0.36	0.
C2	-6.92	177.65	-0.65	-0.85	-5.62	-4.43	0.	0.	8.47	0.
C3	-6.84	0.71	-1.07	-1.62	-0.41	-0.30	7.79	7.20	-0.01	-0.40
C4	7.50	-0.18	209.42	7.62	0.	-0.04	-2.81	-1.07	0.05	1.88
C5	4.42	-0.16	7.43	212.69	-0.05	0.	-1.14	-3.30	0.03	-0.04
C6	2.04	-7.62	0.04	0.09	2.14	12.06	0.	0.00	-7.86	0.
C7	0.95	0.12	-1.93	-1.60	0.	0.	219.59	4.51	0.	9.13
C8	-0.17	0.08	-1.59	-2.07	0.	0.	4.75	210.91	0.02	1.88
C9	0.30	0.	9.50	9.60	0.	0.	-4.10	-2.64	0.	-4.69
H1	-13.25	5.08	-0.55	-0.44	-0.28	-0.27	0.21	0.	0.	0.
H2	0.	11.03	-0.13	-0.36	-0.19	-0.34	0.	0.13	-0.06	0.
H3	11.03	0.	0.05	0.04	15.09	1.14	-0.03	0.	1.19	0.
H4	-0.13	0.05	0.	2.14	0.	0.00	7.44	0.52	0.	0.
H5	-0.36	0.04	2.14	0.	-0.00	0.	0.57	7.15	-0.02	0.11
H6	-0.19	15.09	0.	-0.00	0.	-9.81	0.	0.	-0.07	0.
H7	-0.34	1.14	0.00	0.	-9.81	0.	0.	0.	0.	0.
H8	0.	-0.03	7.44	0.57	0.	0.	0.	2.89	0.	0.03
H9	0.13	0.	0.52	7.15	0.	0.	2.89	0.	0.	0.08
H10	-0.06	1.19	0.	-0.02	-0.07	0.	0.	0.	0.	0.
H11	0.	0.	0.	0.11	0.	0.	0.03	0.08	0.	0.

Supplementary Table 35. Raw values of J couplings in D-tyrosine.

L-tyrosine	C1	C2	C3	C4	C5	C6	C7	C8	C9	H1
C1	0.	95.34	155.42	-3.11	-3.93	-7.23	3.85	3.79	-1.20	171.64
C2	95.34	0.	-7.64	2.49	2.03	177.59	-1.26	-1.38	0.	-6.88
C3	155.42	-7.64	0.	251.47	250.07	0.57	-3.59	-3.69	8.59	-6.80
C4	-3.11	2.49	251.47	0.	-3.24	-0.12	290.91	5.90	-1.81	4.44
C5	-3.93	2.03	250.07	-3.24	0.	-0.06	5.79	289.08	0.80	7.50
C6	-7.23	177.59	0.57	-0.12	-0.06	0.	0.04	0.04	0.	2.05
C7	3.85	-1.26	-3.59	290.91	5.79	0.04	0.	3.27	262.31	-0.16
C8	3.79	-1.38	-3.69	5.90	289.08	0.04	3.27	0.	286.84	0.96
C9	-1.20	0.	8.59	-1.81	0.80	0.	262.31	286.84	0.	0.29
H1	171.64	-6.88	-6.80	4.44	7.50	2.05	-0.16	0.96	0.29	0.
H2	170.02	-6.94	-8.06	6.35	3.98	9.12	0.29	-0.68	0.82	-13.24
H3	-2.78	177.79	0.71	-0.16	-0.17	-7.62	0.08	0.12	0.	11.01
H4	3.39	-0.84	-1.61	212.79	7.61	0.09	-2.06	-1.59	9.59	-0.36
H5	3.30	-0.64	-1.06	7.44	209.58	0.04	-1.59	-1.92	9.50	-0.12
H6	2.46	-4.41	-0.30	0.	-0.04	12.05	0.	0.	0.	-0.34
H7	0.39	-5.62	-0.41	-0.05	0.	2.21	0.	0.	0.	-0.19
H8	0.42	0.	7.20	-3.31	-1.07	0.00	210.84	4.51	-2.64	0.12
H9	0.46	0.	7.79	-1.14	-2.82	0.	4.76	219.54	-4.10	0.
H10	0.33	8.46	-0.01	0.03	0.05	-7.85	0.03	0.	0.	-0.06
H11	0.	0.	-0.39	-0.04	1.88	0.	1.88	9.13	-4.70	0.

L-tyrosine	H2	H3	H4	H5	H6	H7	H8	H9	H10	H11
C1	170.02	-2.78	3.39	3.30	2.46	0.39	0.42	0.46	0.33	0.
C2	-6.94	177.79	-0.84	-0.64	-4.41	-5.62	0.	0.	8.46	0.
C3	-8.06	0.71	-1.61	-1.06	-0.30	-0.41	7.20	7.79	-0.01	-0.39
C4	6.35	-0.16	212.79	7.44	0.	-0.05	-3.31	-1.14	0.03	-0.04
C5	3.98	-0.17	7.61	209.58	-0.04	0.	-1.07	-2.82	0.05	1.88
C6	9.12	-7.62	0.09	0.04	12.05	2.21	0.00	0.	-7.85	0.
C7	0.29	0.08	-2.06	-1.59	0.	0.	210.84	4.76	0.03	1.88
C8	-0.68	0.12	-1.59	-1.92	0.	0.	4.51	219.54	0.	9.13
C9	0.82	0.	9.59	9.50	0.	0.	-2.64	-4.10	0.	-4.70
H1	-13.24	11.01	-0.36	-0.12	-0.34	-0.19	0.12	0.	-0.06	0.
H2	0.	5.11	-0.45	-0.56	-0.28	-0.28	0.	0.21	0.	0.
H3	5.11	0.	0.04	0.05	1.08	15.04	0.	-0.03	1.18	0.
H4	-0.45	0.04	0.	2.13	0.	-0.00	7.13	0.57	-0.02	0.11
H5	-0.56	0.05	2.13	0.	0.00	0.	0.52	7.43	0.	0.
H6	-0.28	1.08	0.	0.00	0.	-9.73	0.	0.	0.	0.
H7	-0.28	15.04	-0.00	0.	-9.73	0.	0.	0.	-0.07	0.
H8	0.	0.	7.13	0.52	0.	0.	0.	2.88	0.	0.08
H9	0.21	-0.03	0.57	7.43	0.	0.	2.88	0.	0.	0.03
H10	0.	1.18	-0.02	0.	0.	-0.07	0.	0.	0.	0.
H11	0.	0.	0.11	0.	0.	0.	0.08	0.03	0.	0.

Supplementary Table 36. Raw values of J couplings in L-tyrosine.

Valine	C1	C2	C3	C4	C5	H1	H2	H3	H4	H5	H6	H7	H8	H9	H10	H11
C1	0.	-0.00	-0.03	0.03	-0.00	0.00	0.00	-0.12	0.09	-0.05	-0.10	0.05	0.12	2.17	-2.11	0.03
C2	-0.00	0.	-0.29	0.30	-0.00	0.00	0.00	0.35	-0.67	0.14	0.66	-0.14	-0.34	0.16	-0.16	0.00
C3	-0.03	-0.29	0.	-0.00	-2.22	-0.05	-1.05	-0.05	0.05	0.00	-0.03	-0.01	0.05	2.33	-0.20	1.62
C4	0.03	0.30	-0.00	0.	2.21	0.04	1.05	-0.05	0.04	0.01	-0.05	-0.00	0.05	0.19	-2.29	-1.59
C5	-0.00	-0.00	-2.22	2.21	0.	-0.00	-0.00	0.72	1.49	0.61	-1.61	-0.57	-0.69	-0.48	0.47	0.00
H1	0.00	0.00	-0.05	0.04	-0.00	0.	0.01	0.02	0.07	0.10	-0.07	-0.10	-0.02	4.32	-4.22	-0.04
H2	0.00	0.00	-1.05	1.05	-0.00	0.01	0.	-0.31	-0.61	-1.53	0.65	1.50	0.37	-1.88	1.96	-0.09
H3	-0.12	0.35	-0.05	-0.05	0.72	0.02	-0.31	0.	0.05	-0.01	0.06	-0.88	0.00	1.08	-2.81	0.64
H4	0.09	-0.67	0.05	0.04	1.50	0.07	-0.61	0.05	0.	-0.01	0.02	0.11	-0.04	13.73	-3.15	2.37
H5	-0.05	0.14	0.00	0.01	0.61	0.10	-1.53	-0.01	-0.01	0.	-0.09	0.06	0.67	-2.00	-4.62	2.18
H6	-0.10	0.66	-0.03	-0.05	-1.61	-0.07	0.65	0.06	0.02	-0.09	0.	0.01	-0.05	3.45	-38.00	-2.37
H7	0.05	-0.14	-0.01	-0.00	-0.57	-0.10	1.50	-0.88	0.11	0.06	0.01	0.	0.01	5.58	2.00	-2.18
H8	0.12	-0.34	0.05	0.05	-0.69	-0.02	0.37	0.00	-0.04	0.67	-0.05	0.01	0.	2.44	-1.03	-0.65
H9	2.17	0.16	2.33	0.19	-0.48	4.32	-1.89	1.08	13.73	-2.00	3.45	5.58	2.44	0.	0.01	2.00
H10	-2.11	-0.16	-0.20	-2.29	0.47	-4.22	1.96	-2.81	-3.15	-4.62	-38.00	2.00	-1.03	0.01	0.	-2.00
H11	0.03	0.00	1.62	-1.59	0.00	-0.04	-0.09	0.64	2.37	2.18	-2.37	-2.18	-0.65	2.00	-2.00	0.

Supplementary Table 37. Numerical values of the fractional deviation $[J(L) - J(D)]/[J(L) + J(D)]/2$ for valine.

D-valine	C1	C2	C3	C4	C5	H1	H2	H3	H4	H5	H6	H7	H8	H9	H10	H11
C1	0.	108.01	127.74	124.28	-7.34	165.19	-6.18	-5.72	-6.30	-5.07	-6.92	-4.82	-5.08	-0.26	6.78	-0.41
C2	108.01	0.	-6.81	-5.04	169.87	-7.25	164.59	2.01	2.00	10.36	1.00	11.88	2.86	-3.58	-4.21	7.03
C3	127.74	-6.81	0.	-7.76	1.81	-7.22	6.10	177.56	169.34	170.24	2.69	1.22	10.72	-0.09	-0.29	0.03
C4	124.28	-5.04	-7.76	0.	-0.09	-6.91	1.90	11.25	2.59	1.21	177.83	170.78	169.63	-0.24	1.26	0.36
C5	-7.34	169.87	1.81	-0.09	0.	9.52	-9.76	-0.09	-0.03	-0.11	-0.19	-0.21	-0.18	8.34	4.92	-7.62
H1	165.19	-7.25	-7.22	-6.91	9.52	0.	3.23	2.65	13.23	4.31	14.17	4.79	2.70	-0.25	0.72	-0.10
H2	-6.18	164.59	6.10	1.90	-9.76	3.23	0.	-0.24	-0.31	1.45	-0.16	0.20	-0.16	8.32	0.08	-0.45
H3	-5.72	2.01	177.56	11.25	-0.09	2.65	-0.24	0.	-13.06	-14.21	-0.15	0.08	3.21	0.04	0.09	-0.04
H4	-6.30	2.00	169.34	2.59	-0.03	13.23	-0.31	-13.06	0.	-14.10	-0.17	-0.23	-0.15	0.04	-0.07	-0.02
H5	-5.07	10.36	170.24	1.21	-0.11	4.31	1.45	-14.21	-14.10	0.	-0.25	-0.10	0.03	-0.08	0.09	-0.01
H6	-6.92	1.00	2.69	177.83	-0.19	14.17	-0.16	-0.15	-0.17	-0.25	0.	-13.95	-13.68	0.02	-0.06	0.28
H7	-4.82	11.88	1.22	170.78	-0.21	4.79	0.20	0.08	-0.23	-0.10	-13.95	0.	-14.07	-0.04	0.	0.32
H8	-5.08	2.85	10.72	169.63	-0.18	2.70	-0.16	3.21	-0.15	0.03	-13.68	-14.07	0.	-0.01	0.13	-0.07
H9	-0.26	-3.58	-0.09	-0.24	8.34	-0.25	8.32	0.04	0.04	-0.08	0.02	-0.04	-0.01	0.	-9.10	0.
H10	6.78	-4.21	-0.29	1.26	4.92	0.72	0.08	0.09	-0.07	0.09	-0.06	0.	0.13	-9.10	0.	0.06
H11	-0.41	7.03	0.03	0.36	-7.62	-0.10	-0.45	-0.04	-0.02	-0.01	0.28	0.32	-0.07	0.	0.06	0.

Supplementary Table 38. Raw values of J couplings in D-valine.

L-val.	C1	C2	C3	C4	C5	H1	H2	H3	H4	H5	H6	H7	H8	H9	H10	H11
C1	0.	107.75	124.48	127.99	-7.32	165.43	-6.20	-5.10	-6.92	-4.82	-6.29	-5.07	-5.74	6.48	-0.18	-0.42
C2	107.75	0.	-5.11	-6.84	169.87	-7.26	164.81	2.86	1.00	11.91	2.00	10.37	2.02	-4.19	-3.58	7.03
C3	124.48	-5.11	0.	-7.75	-0.10	-6.89	1.90	169.67	177.71	170.81	2.60	1.22	11.26	1.23	-0.24	0.35
C4	127.99	-6.84	-7.75	0.	1.87	-7.22	6.15	10.73	2.70	1.23	169.34	170.34	177.63	-0.29	-0.09	0.04
C5	-7.32	169.87	-0.10	1.87	0.	9.47	-9.75	-0.19	-0.19	-0.21	-0.02	-0.12	-0.09	5.10	7.98	-7.62
H1	165.43	-7.26	-6.89	-7.22	9.47	0.	3.26	2.70	14.19	4.78	13.26	4.32	2.66	0.69	-0.26	-0.09
H2	-6.20	164.81	1.90	6.15	-9.75	3.26	0.	-0.18	-0.16	0.19	-0.32	1.45	-0.24	0.25	8.52	-0.41
H3	-5.10	2.86	169.67	10.73	-0.19	2.70	-0.18	0.	-13.68	-14.09	-0.16	0.03	3.21	0.13	-0.02	-0.07
H4	-6.92	1.00	177.71	2.70	-0.19	14.19	-0.16	-13.68	0.	-13.95	-0.18	-0.26	-0.15	-0.06	0.01	0.29
H5	-4.82	11.91	170.81	1.23	-0.21	4.78	0.19	-14.09	-13.95	0.	-0.23	-0.11	0.08	0.	-0.03	0.33
H6	-6.29	2.00	2.60	169.34	-0.02	13.26	-0.32	-0.16	-0.18	-0.23	0.	-14.08	-13.08	-0.06	0.05	-0.02
H7	-5.07	10.37	1.22	170.34	-0.12	4.32	1.45	0.03	-0.26	-0.11	-14.08	0.	-14.24	0.09	-0.08	-0.01
H8	-5.74	2.02	11.26	177.63	-0.09	2.66	-0.24	3.21	-0.15	0.08	-13.08	-14.24	0.	0.1	0.04	-0.04
H9	6.48	-4.19	1.23	-0.29	5.10	0.69	0.25	0.13	-0.06	0.	-0.06	0.09	0.1	0.	-9.16	0.07
H10	-0.18	-3.58	-0.24	-0.09	7.98	-0.26	8.52	-0.02	0.01	-0.03	0.05	-0.08	0.04	-9.16	0.	0.
H11	-0.42	7.03	0.35	0.04	-7.62	-0.09	-0.41	-0.07	0.29	0.33	-0.02	-0.01	-0.04	0.07	0.	0.

Supplementary Table 39. Raw values of J couplings in L-valine.

References

[1] Löwdin, P.-O. A note on the quantum-mechanical perturbation theory. *The Journal of Chemical Physics* **19**, 1396–1401 (1951).

[2] Slichter, C. *Principles of magnetic resonance, Corrected 3rd printing*, vol. 1 of *Springer Series in Solid-State Sciences* (Springer, 1996).

[3] Varela, S., Mujica, V. & Medina, E. Effective spin-orbit couplings in an analytical tight-binding model of dna: Spin filtering and chiral spin transport. *Physical Review B* **93**, 155436 (2016).

[4] López, A., Varela, S. & Medina, E. Radiation modulated spin coupling in a double-stranded dna model. *Journal of Physics: Condensed Matter* **34**, 135301 (2022).

[5] Santos, J. *et al.* Chirality-induced electron spin polarization and enantiospecific response in solid-state cross-polarization nuclear magnetic resonance. *ACS nano* **12**, 11426–11433 (2018).

[6] San Sebastian, E. *et al.* Enantiospecific response in cross-polarization solid-state nuclear magnetic resonance of optically active metal organic frameworks. *Journal of the American Chemical Society* **142**, 17989–17996 (2020).

[7] Wang, K. DNA-based single-molecule electronics: from concept to function. *Journal of functional biomaterials* **9**, 8, 31pp (2018).

[8] Haeberlen, U. *High Resolution NMR in Solids: Selective Averaging* (Academic Press, New York, 1976).

[9] Abragam, A. *The principles of nuclear magnetism* (Oxford university press, 1961).

[10] Michel, D. & Engelke, F. Cross-polarization, relaxation times and spin-diffusion in rotating solids. *Solid-State NMR III Organic Matter* **32**, 69–125 (1994).

[11] Stejskal, E. & Memory, J. High resolution NMR in the solid state: fundamentals of CP/MAS, 1st edition. *Oxford University Press* (1994).

[12] Cremer, D. & Gräfenstein, J. Calculation and analysis of nmr spin–spin coupling constants. *Phys. Chem. Chem. Phys.* **9**, 2791–2816 (2007). URL `http://dx.doi.org/10.1039/B700737J`.
