# Supplementary material for: Enantiospecificity in NMR enabled by chirality-induced spin selectivity
Source: Nat Commun. 2024 Aug 27;15:7367. doi: 10.1038/s41467-024-49966-8 (PMC11349874; doi:10.1038/s41467-024-49966-8)
Supplement: Supplementary file 3 — Description of Additional Supplementary Files [file 41467_2024_49966_MOESM3_ESM.pdf]

Description of Additional Supplementary Files

File Name: Supplementary Data 1
Description: ZIP file containing text files with the atomic coordinates used in this study.
